# Bayesian Confidence in Optimal Decisions

**DOI:** 10.1037/rev0000472

**Published:** 2024-07-18

**Authors:** Joshua Calder-Travis, Lucie Charles, Rafal Bogacz, Nick Yeung

**Affiliations:** 1 Department of Experimental Psychology, University of Oxford; 2 Institute of Neurophysiology and Pathophysiology, Universitätsklinikum Hamburg-Eppendorf; 3 Institute of Cognitive Neuroscience, University College London; 4 Nuffield Department of Clinical Neurosciences, Medical Research Council Brain Network Dynamics Unit, University of Oxford

**Keywords:** perceptual decisions, confidence, drift diffusion model, normative, Bayesian

## Abstract

The optimal way to make decisions in many circumstances is to track the difference in evidence collected in favor of the options. The drift diffusion model (DDM) implements this approach and provides an excellent account of decisions and response times. However, existing DDM-based models of confidence exhibit certain deficits, and many theories of confidence have used alternative, nonoptimal models of decisions. Motivated by the historical success of the DDM, we ask whether simple extensions to this framework might allow it to better account for confidence. Motivated by the idea that the brain will not duplicate representations of evidence, in all model variants decisions and confidence are based on the same evidence accumulation process. We compare the models to benchmark results, and successfully apply four qualitative tests concerning the relationships between confidence, evidence, and time, in a new preregistered study. Using computationally cheap expressions to model confidence on a trial-by-trial basis, we find that a subset of model variants also provide a very good to excellent account of precise quantitative effects observed in confidence data. Specifically, our results favor the hypothesis that confidence reflects the strength of accumulated evidence penalized by the time taken to reach the decision (Bayesian readout), with the penalty applied not perfectly calibrated to the specific task context. These results suggest there is no need to abandon the DDM or single accumulator models to successfully account for confidence reports.

Can the normative and empirically successful drift diffusion model (DDM) of decisions and response times (RTs) also account for confidence reports? We address this question—which we unpack in the proceeding paragraphs—using a range of approaches, including trial-by-trial modeling of confidence reports.

Computing confidence, a sense of the probability of being correct, is potentially highly beneficial in a wide range of situations. Consider an owl flying after a glimpse of a gray object in the grass. The energy it exerts in the chase would sensibly be moderated by the probability it saw a mouse. There is strong evidence that human confidence is reliably related to objective performance, and that humans do indeed use confidence to regulate their decisions and learning in a wide variety of situations (Bahrami et al., 2010; Balsdon et al., 2020; Boldt et al., 2019; Carlebach & Yeung, 2020; Desender et al., 2018, 2019; Drugowitsch et al., 2019; Harun et al., 2020; Kepecs & Mainen, 2012; Sanders et al., 2016; van den Berg, Zylberberg, et al., 2016). Changes in the strength of the relationship between confidence and objective performance have been linked to psychological disorders (Hauser et al., 2017; Rouault et al., 2018). Additionally, an understanding of the mechanisms responsible for confidence may illuminate how the brain represents evidence, probability, and probability distributions. Because of this importance, there is growing interest in understanding the computations responsible for confidence. A great deal of work has already gone into building computational models of confidence (e.g., Balsdon et al., 2020; Kiani et al., 2014; Pleskac & Busemeyer, 2010; van den Berg, Anandalingam, et al., 2016). However, models of confidence have often posited different mechanisms to those found in the normative and empirically successful DDM of perceptual decisions (discussed in detail below; Ratcliff & McKoon, 2008; e.g., De Martino et al., 2013; Kiani et al., 2014; van den Berg, Anandalingam, et al., 2016; Zylberberg et al., 2012). The aim of this article is a unifying one: We explore whether confidence reports can also be explained within the framework of a normative and successful model of perceptual decisions.

Therefore, we begin this exploration by considering normative models for the integration of noisy evidence into categorical decisions. We use “normative model” and “optimal model” to refer to models in which the observer achieves the maximum possible reward given the information that is assumed to be available to them (Rahnev & Denison, 2018). The fact that human behavior is variable given identical perceptual stimuli suggests that measurements of perceptual evidence are corrupted by noise (Drugowitsch et al., 2016). Humans can trade speed for accuracy (Garrett, 1922; Heitz, 2014; Wickelgren, 1977), suggesting that a stream of evidence samples are received over time and can be averaged to reduce noise and increase performance (Bogacz et al., 2006; Ratcliff & McKoon, 2008). It is often assumed that given two options, two streams of evidence samples are generated. For example, in a binary choice regarding which of two arrays contains most dots, the observer might receive two sets of noisy evidence samples, one corresponding to the number of dots in the left array and one corresponding to the number of dots in the right array. Under certain common assumptions, the algorithm that leads to maximal reward involves comparing evidence samples for the two alternatives and accumulating this difference over time (Bogacz et al., 2006; Gold & Shadlen, 2001; Tajima et al., 2019). This procedure makes intuitive sense: If we have two alternatives, evidence for one option should automatically be evidence against the other option. When the strengths of the evidence signals for the correct and incorrect option are also constant across decisions or are known for a specific decision, the optimal stopping rule involves waiting until a specific accumulated difference in evidence is reached (Bogacz et al., 2006; Gold & Shadlen, 2007; Moran, 2015; Wald & Wolfowitz, 1948).

One model in which observers compare evidence samples for two alternatives and accumulate the difference is the DDM (Figure 1; Bogacz et al., 2006; Ratcliff & McKoon, 2008). The average rate of accumulation is called the drift rate. The drift rate is determined by the difference in mean evidence signals for the two alternatives (rather than the noisy samples of those signals). Whilst the drift rate determines the average path of the accumulator, noise in evidence samples causes the accumulator to fluctuate randomly around this path. The accumulator begins near zero, and a decision is triggered when the accumulator reaches either a positive or negative threshold. The threshold reached determines the choice, and the time taken to reach it determines the reaction time.

There is good reason to think that brains will use decision algorithms with optimal properties, such as the DDM: From judging car speed and crossing a road to deciding which animal is on the horizon, converting continuous sensory variables into discrete decisions is, and always has been, a foundational cognitive ability of animals and humans (Green & Swets, 1966). This reasoning is a central motivation of our aim to examine whether the DDM can be used to also account for confidence. Note we do not claim that perceptual decisions of animals and humans are always (or even often) optimal relative to the statistics of a specific task. For example, we are agnostic regarding the question of whether humans set decision thresholds with a height that is optimal for the task at hand (e.g., Evans & Brown, 2017; Malhotra et al., 2017). Indeed, humans will certainly not be optimal if they have not had sufficient time to learn the statistics of the task, or have not received sufficient feedback to calibrate their decision making (Evans & Brown, 2017; Evans et al., 2020). Instead, we only claim that there are strong theoretical reasons to think the decision machinery of the brain will have evolved to use the evidence accumulation mechanism, exemplified by the DDM, that is normative for typical perceptual decision-making situations. This is what motivates our investigation of DDM-based accounts of confidence. For confidence itself, as a less foundational cognitive ability, the evolutionary push toward optimality has presumably been weaker. Furthermore, whereas for decisions there is a clear “reward” to be maximized, for confidence—that is, evaluations of decisions to take certain actions—it is less clear what reward should be maximized. Accordingly, we consider a range of possible confidence readout mechanisms. In any case, we do not claim that confidence will necessarily be optimal for the statistics of a specific task (a point we expand further below).

Aside from theoretical support, the DDM also enjoys 50 years of empirical support (Ratcliff et al., 2016; although debates over the extent of this success continue; Kirkpatrick et al., 2021; Rafiei & Rahnev, 2020; Ratcliff & Kang, 2021). The model and extensions account well for the shape of response time distributions, how accuracy changes with task difficulty, and how response time changes with task difficulty (Ratcliff, 1978; Ratcliff & McKoon, 2008). Assuming trial-to-trial variability in drift rate, and the starting point of the accumulator, the model can also explain why in some cases error responses are faster than correct responses but in other cases correct responses are faster than errors (Ratcliff et al., 1999; Ratcliff & McKoon, 2008). It has proved a unifying framework, explaining data in perceptual decisions (Ratcliff & McKoon, 2008), value-based decisions (Lee & Usher, 2021; Milosavljevic et al., 2010), and memory tasks (Ratcliff, 1978). Hence, there are additionally strong empirical reasons for exploring whether the DDM can account for confidence reports.

Unfortunately, it turns out that for all the success of the DDM explaining patterns in choices and response times, the model cannot straightforwardly account for confidence reports (Rosenbaum et al., 2022; Yeung & Summerfield, 2014). The reason is that a decision is triggered when the accumulator crosses a fixed threshold. Therefore, at the time someone commits to a decision, they will always have the same accumulated difference in evidence favoring the selected option (Figure 1). This apparently leads to the conclusion that people and animals should have equal confidence in all attempts at a task, if we adopt the reasonable assumption that a decision maker’s confidence should reflect the strength of evidence in favor of their chosen option. In stark contrast, confidence data show a rich set of context-dependent patterns (detailed in Models section; Pleskac & Busemeyer, 2010), such as a strong negative relationship between confidence and response time in certain settings (Pleskac & Busemeyer, 2010; Rosenbaum et al., 2022; Vickers & Packer, 1982).

One successful approach to accounting for confidence within the DDM has focused on the idea that people may continue to accumulate evidence following a decision (Moran et al., 2015; Pleskac & Busemeyer, 2010). These extra evidence samples can drive variability in confidence reports even when task difficulty is fixed. Importantly, on trials in which the observer was correct, extra samples will tend to support their decision. As a result, people will have greater confidence in correct responses than in errors. Research on changes of mind, and work exploring how confidence is influenced by evidence at different points in time, strongly suggests that evidence accumulation does indeed continue following a decision (Charles & Yeung, 2019; Moran et al., 2015; Resulaj et al., 2009; Rosenbaum et al., 2022; van den Berg, Anandalingam, et al., 2016). Nevertheless, alone, this extension does not immediately generate a relationship between confidence and response time.

Pleskac and Busemeyer (2010) included the idea of trial-to-trial variability in drift rate in their DDM-based model of decisions and confidence, the two-stage dynamic signal detection theory (2DSD). Drift-rate variability allows the model to explain why confidence decreases with response time. On a trial in which the drift rate is high, the threshold will be reached quickly triggering a quick response, but this high drift rate will also drive rapid accumulation following a decision. The accumulator will reach a large value, generating high confidence. Note that it does not matter whether this variability in drift rate is produced because evidence strength provided by the stimulus varies or because the internal representation of constant evidence strength stimuli varies from trial-to-trial (Pleskac & Busemeyer, 2010; Ratcliff et al., 1999, 2016). In either case, confidence will now decrease with response time. This means that the basic DDM model, coupled with evidence accumulation following a decision, can easily account for a relationship between confidence and time in contexts where stimulus evidence strength varies from trial-to-trial. More challenging is the case in which stimulus evidence strength does not vary on a trial-by-trial basis (but confidence and response time remain related; e.g., Baranski & Petrusic, 1998). Unless otherwise noted, we will only consider this more challenging case throughout. In this situation, 2DSD can still account for a relationship between confidence and response time by assuming that there is trial-to-trial variability in drift rate, specifically because of variability in how constant evidence strength stimuli are processed (Pleskac & Busemeyer, 2010).

Although the 2DSD model explains an impressive number of patterns in confidence, Pleskac and Busemeyer (2010) noted that it could only provide a partial explanation for the relationship observed between confidence and response time in data they collected. Specifically, 2DSD could not account for the strength of this relationship, predicting a weaker relationship than was observed (Pleskac & Busemeyer, 2010, p.881). We will see that this is also the case in data collected here. Later work on a closely related model did not address this quantitative deficiency either (Moran et al., 2015). Moreover, because 2DSD relies on evidence accumulation following a decision to account for variability in confidence reports, the model struggles to explain such variability when confidence reports are given simultaneously with a decision (e.g., Kiani et al., 2014). Additional assumptions are required, for example, the assumption that observers make covert choices without immediately responding (Ratcliff, 2006). Similar considerations apply to the closely related model put forward by Moran et al. (2015).

Thus, although substantial progress has been made in developing DDM-based models of confidence, important limitations remain. Many researchers have therefore considered models that provide clearer explanations of confidence, but that rely on alternative decision-making mechanisms to the empirically successful and normative DDM (De Martino et al., 2013; Kepecs et al., 2008; Kiani et al., 2014; Moreno-Bote, 2010; van den Berg, Anandalingam, et al., 2016; Vickers, 1979; Vickers & Packer, 1982; Yeung & Summerfield, 2014; Zylberberg et al., 2012). Examples of such alternatives are the race model and the partially correlated accumulators model, in which two evidence accumulators simultaneously accumulate (at least partially) distinct information (Kiani et al., 2014; Moreno-Bote, 2010; Ratcliff & Smith, 2004; Smith & Ratcliff, 2009; Teodorescu & Usher, 2013; Vickers, 1970). If a decision is triggered by either one of the two accumulators crossing a threshold, then the difference in evidence between the alternatives—which can only be calculated by taking into account the value of both accumulators—is no longer constrained to a fixed value at decision time (contrasting with the DDM) and can naturally explain confidence reports (Vickers & Packer, 1982; Yeung & Summerfield, 2014). Although the standard race model itself (one independent accumulator for each stimulus; van Ravenzwaaij et al., 2020) exhibits deficits in its account of choice and response time data (Kirkpatrick et al., 2021; Miller & Ulrich, 2003; Ratcliff & Smith, 2004; Teodorescu & Usher, 2013), variants of the standard race model have performed well on choice and response time data (Kirkpatrick et al., 2021; Usher & McClelland, 2001; van Ravenzwaaij et al., 2020). Another line of research has explored models with multiple simultaneous evidence accumulators, one for each possible combination of choice and confidence, with the winning accumulator determining both (Ratcliff & Starns, 2009, 2013). Again, such a framework naturally generates variability in confidence reports, and such models have provided a good account of responses and response times for tasks where choice and confidence are reported simultaneously. Nevertheless, all such models remain nonnormative with regards to the two-alternative perceptual decision itself: As discussed, a single accumulation of the difference in evidence is optimal under common assumptions (Bogacz et al., 2006; Gold & Shadlen, 2001; Tajima et al., 2019).

A further alternative, arguably nonnormative approach, is to consider models in which separate evidence accumulation mechanisms are responsible for decisions and for confidence (Fleming & Daw, 2017). For example, such models may postulate one evidence accumulation for decisions and a different evidence accumulation for confidence (Balsdon et al., 2020; Ganupuru et al., 2019; Jang et al., 2012). Even if each accumulation individually uses the DDM, there is a clear sense in which two separate accumulators are nonnormative: The brain would then effectively have two perceptual systems, both accumulating evidence about the same thing, while costing twice as much in terms of energy consumption (Lennie, 2003). Moreover, if the observer did have two noisy versions of an accumulator, the observer could improve performance by averaging the information in these two accumulators, leading to a single accumulator with reduced noise.

In this context of a range of successful confidence models relying on nonnormative decision mechanisms, the present research was motivated by a desire to build on the work of Pleskac and Busemeyer (2010) and Moran et al. (2015), to explore how far we can account for qualitative and precise quantitative patterns in confidence data using normative perceptual decision mechanisms. Specifically, we aim to explore single accumulator models—that is, models in which both decisions and confidence are generated by the same evidence accumulator—featuring the DDM’s accumulation mechanism, that is known to be optimal under certain common assumptions (Bogacz et al., 2006; Tajima et al., 2019). In comparison to previous work, we attempt a more systematic and broader exploration of the performance of such models.

Motivated by the strong relationship between confidence and response time, and by the struggle of previous DDM approaches to account for the strength of this relationship, we consider two key ways in which confidence might depend on decision times: first, if the threshold for committing to a choice varied over time and, second, if decision time itself was directly factored into the computation of confidence. Before turning to these ideas, which can both be motivated on normative grounds, we briefly address a simpler possibility: As part of a heuristic approach to producing confidence reports, observers use time as a cue for confidence (Audley, 1960; Pleskac & Busemeyer, 2010; Ratcliff, 1978; Zakay & Tuvia, 1998). While this could clearly generate a strong relationship between confidence and time, the relationship between confidence and time is more complex than that which would be predicted by a simple heuristic model (Pleskac & Busemeyer, 2010). As we will see later, the direction of this relationship is known to reverse depending on the context. Furthermore, confidence varies with other factors over and above response time (Kiani et al., 2014). These considerations do not rule out a sophisticated heuristic observer who flexibly combines cues depending on the context, and we return to this possibility in the discussion.

If decision thresholds decrease over time, meaning a smaller value of accumulated evidence is required to trigger a decision for later versus earlier decisions (Drugowitsch et al., 2012; Malhotra et al., 2017), then a clear relationship between confidence and response time would arise: Later decisions will be made with a smaller balance of evidence in their favor, and so confidence will be lower. Within the DDM framework, decreasing decision thresholds can be optimal when the difficulty of the task is unknown to the observer, whether this is due to external factors such as variability in stimuli across trials or internal factors such as variability in the processing of constant evidence strength stimuli, generating trial-to-trial drift-rate variability (Drugowitsch et al., 2012; Malhotra et al., 2018; Moran, 2015; Tajima et al., 2016, 2019). The intuition here is that if little evidence has been accumulated after a lengthy period of deliberation, the task is likely to be very difficult (Malhotra et al., 2018). When a task is very difficult, there is little to gain from accumulating evidence, and doing so would cost the observer time. There is some evidence that decision thresholds do in fact decrease in some situations (Glickman et al., 2019; Glickman & Usher, 2019; Malhotra et al., 2017; Palestro et al., 2018), although there is also conflicting evidence (Hawkins et al., 2015; Pardo-Vazquez et al., 2019; Voskuilen et al., 2016) and mixed results (Evans et al., 2020).

While a subtlety, it is very important to distinguish two effects of time on confidence: within and between conditions (Vickers & Packer, 1982). Within a condition, decreasing thresholds will mean that the balance of evidence supporting decisions decreases with decision time, and therefore confidence may also follow this pattern. This contrasts with the relationship expected when comparing two conditions with different emphasis on speed versus accuracy. Consider a new experimental condition in which we ask participants to emphasize accuracy over speed. From the perspective of the model, participants can achieve slower and more accurate responses by shifting their decision thresholds outward, requiring more evidence to be gathered before a response is triggered (Ratcliff & McKoon, 2008). Consequently, participants will be more confident in their choices, because they will have gathered more evidence for them. The overall effect is that in this new condition, on average, response times are slower, while accuracy and confidence are greater (Pleskac & Busemeyer, 2010; Vickers & Packer, 1982). This example shows that a condition associated with slower response times may generate higher confidence. Only within a single condition do we expect decreasing thresholds to generate a strong negative relationship between response time and confidence.

A clear relationship between confidence and response time could also arise if confidence reflects a Bayesian readout of the probability of being correct, based on the final state of the DDM accumulator and total deliberation time (Aitchison et al., 2015; Kiani et al., 2014; Meyniel et al., 2015; Pew, 1969; Sanders et al., 2016; Vickers & Packer, 1982; under standard assumptions, only time and the final state of the DDM accumulator are relevant, and the accumulator’s path up to that point adds no relevant information; Moreno-Bote, 2010). If the difficulty of the task is unknown to the observer, and if we consider a fixed value of the accumulator, then in a range of settings Bayesian confidence decreases with the time spent accumulating that evidence (Moran, 2015; Moreno-Bote, 2010). An intuition for this effect is the following. The average rate at which you have accumulated evidence gives you information about the difficulty of a trial. If you accumulate 10 units of evidence in 1 s you are accumulating faster than if you accumulate 10 units in 2 s. Hence, in the first case you are more likely to be on an easier trial than in the second case. As a result, you can be more confident in your response in the first case. This line of reasoning applies equally to a situation in which the observer is free to respond when they like, and a situation in which response time is determined by the researcher: In either case, beyond the quantity of accumulated evidence, the amount of time it took to accumulate that evidence is also relevant (Moreno-Bote, 2010). If, in a free response situation, response time determines the amount of time the observer spends accumulating evidence, then confidence will also decrease with response time. We refer to the effect of time spent accumulating evidence as the “time penalty for confidence.” An important point is that difficulty can be unknown to the observer—and hence the time penalty for confidence applied—either because the evidence strength provided by the stimulus varies on a trial-by-trial basis or because the quality of information extracted from constant evidence strength stimuli varies on a trial-by-trial basis (Moran, 2015; Ratcliff et al., 1999, 2016). As discussed above, throughout we only consider the more challenging case for confidence models, namely the case of constant evidence strength stimuli. Nevertheless, it is worth noting that either case generates trial-by-trial variability in drift rate, and a Bayesian readout for confidence that depends not just on evidence accumulated but also time.

We take these ideas, motivated theoretically and empirically and combine them to build a number of variants of a core DDM model that we then compare against new experimental data. Building on the work of Pleskac and Busemeyer (2010) and van den Berg, Anandalingam, et al. (2016) showing that postdecision evidence has an important role to play, we include postdecision evidence accumulation in all model variants. Other features are only included in subsets of the model variants. First, we consider the possibility that, as in 2DSD, drift-rate variability helps account for the relationship between confidence and time to some extent (Pleskac & Busemeyer, 2010). Second, we consider the possibility that the decision threshold used to trigger a decision may decrease over time (Drugowitsch et al., 2012; Malhotra et al., 2017). Finally, we consider different ways confidence could be read out: Confidence could reflect only the final state of the accumulator (Pleskac & Busemeyer, 2010), or it could also feature a time penalty for confidence. Bayes rule prescribes a specific relationship between evidence, time, and confidence. This relationship is sensitive to the strength of various sources of variability in the evidence accumulation. The time penalty could match that used by a calibrated Bayesian observer who has perfect knowledge of the statistics of the task (such a time penalty could possibly be learnt by association; Kiani et al., 2014; Pew, 1969; Vickers & Packer, 1982). Alternatively, the time penalty could reflect a miscalibrated Bayesian readout (Drugowitsch et al., 2014), a Bayesian readout of the probability of being correct, but one based on imperfect estimates for the strength of the various sources of variability.

Our goal is to determine (a) which DDM model variant fits confidence data best and (b) whether any of the models can explain the range of qualitative and quantitative effects observed in confidence data. In this way, we aim to answer the overarching question that we posed at the outset, of whether a normative and successful model of decisions, the DDM, can also account for confidence reports, specifically in relation to previously described empirical challenges that have led many models of confidence to depart from this normative framework. Of particular interest will be whether any of the variants can overcome the limitation of 2DSD in accounting for the strength of the relationship between confidence and decision time. One of the variants we consider is closely related to the 2DSD model. To test the power of the models, we first consider how the models fare against benchmark findings and whether they can account for findings that have previously been difficult to explain using a single framework. Next, we note qualitative predictions of the model variants, which we test in a preregistered study. We then use newly developed, computationally cheap expressions to fit the models using trial-by-trial predictions for confidence (Calder-Travis et al., 2023). This approach allows us to examine whether any DDM model can account for the precise quantitative effects observed in confidence data. If one of the DDM models considered can provide a better account of the patterns observed than previous DDM-based models of confidence, it will strengthen support for the view that one of the most basic cognitive functions, perceptual decision making, is generated through a mechanism with optimal properties. A corollary would be that we do not need to abandon the idea that animals and humans use decision mechanisms with normative properties for the sake of accounting for confidence reports. While this is the main goal, we will end with a parsimonious account of decisions and confidence that will provide a unified explanation for a large range of empirically observed effects.

Although our assessment of DDM models of confidence will be multifaceted, encompassing consideration of previous findings and benchmarks, qualitative predictions, and quantitative modeling, we do not attempt to simultaneously model confidence, choices, and response times. Instead, our approach is to model confidence given choice and response time. By not modeling choices and response times directly, trial-by-trial modeling of time-varying stimuli and time-varying decision thresholds becomes possible using recently derived expressions (Calder-Travis et al., 2023). Modeling choices, response times, and confidence simultaneously on a trial-by-trial basis is an important ultimate goal. However, such an approach is currently not feasible due to the excessive computational cost of producing trial-by-trial predictions for response and response times (some existing approaches are given by Ratcliff, 1980; Shinn et al., 2020; Smith, 2000; Smith & Ratcliff, 2022). Only limited cases are covered by existent fast mathematical solutions that make trial-by-trial modeling possible, such as the case where decision thresholds and evidence strength are fixed throughout a trial (Navarro & Fuss, 2009), or if evidence accumulation only occurs at a limited number of time points (Park et al., 2016). Perhaps due to these limitations in the kinds of trial-by-trial modeling of decisions and response times that are possible, previous studies of confidence in evidence accumulation models have either ignored trial-by-trial data or used stimuli without such richness and have instead modeled data on a condition-by-condition basis (e.g., one likelihood function is produced for high stimulus evidence trials and a second likelihood function is produced for low stimulus evidence trials), approximating all trials from the same condition as the same (Moran et al., 2015; Pleskac & Busemeyer, 2010; Ratcliff & Starns, 2013; van den Berg, Anandalingam, et al., 2016).

Here, our goal was to exploit the rich information available in single-trial data to adjudicate between competing models of confidence. Thus, our analysis allows for complexities such as time-dependent thresholds and fluctuating stimuli that provide evidence that varies within trials, fitting models to trial-by-trial varying individual confidence reports (more than 30,000 in our case), rather than the conventional alternative of condition-by-condition modeling of choices, response times and confidence. In this regard, we chose to limit the breadth of our modeling approach (focusing on confidence, rather than fitting also choices and response times), so that we could make a substantial advance in the depth of our modeling, by modeling DDM-based confidence in a fluctuating-stimulus task on a trial-by-trial basis. This takes us a step closer to the ideal of broadly applicable trial-by-trial modeling of choices, response times, and confidence, and allows us to model confidence to a degree of precision that would not otherwise be possible.

This approach represents a methodological advance that we wish to highlight. Nevertheless, our ultimate aim is to explore whether a DDM-based model can also account for confidence in addition to choices and response times, not instead of choices and response times. To some extent we take for granted the extensive body of evidence showing that the DDM is a good model of both decisions and response times (Lee & Usher, 2021; Milosavljevic et al., 2010; Ratcliff, 1978; Ratcliff et al., 1999, 2016; Ratcliff & McKoon, 2008). Indeed, the success of the DDM in accounting for decisions and response times is a major motivation for this work exploring DDM-based models of confidence. Furthermore, we do not aim to answer the question “what is the best combined model of decisions and confidence?” but rather we work from the perspective that the DDM is a theoretically and empirically important model and aim to answer the currently open question of whether it can be extended to account for confidence reports. Notwithstanding these considerations, a DDM variant that can only account for confidence reports in a parameter range that produces poor predictions for choices and response times would not be a DDM model that can also account for confidence. Hence, once we have completed our trial-by-trial fitting to confidence, we will assess whether the parameters from fitting to confidence lead to reasonable predictions for choices and response times. In the course of this assessment, we will perform a form of hybrid fitting, fitting to confidence on a trial-by-trial basis, but additionally fitting to some aspects of choices and response times. This will produce a DDM variant that fits well to choices and response times, with almost no compromise in the ability of the model to fit confidence. We will return to this point throughout, and in General Discussion section. In General Discussion section, we also discuss how our field could work toward simultaneous trial-by-trial modeling of choices, response times and confidence in rich, dynamic-stimuli tasks, and where our work fits in to progress toward that goal.

## Models

We start by setting out in detail the models that we will investigate. We then assess whether the models can explain benchmark findings and account for results that have previously been difficult to explain. We finish the section by describing four qualitative tests of the models that we will apply in the next sections.

Prior to setting out the models, we will describe two different contexts that we will apply the models to. These contexts are termed the “free response” (also known as “information controlled”) and “interrogation” (also known as “time controlled” or “response signal”) conditions (Bogacz et al., 2006; Moran, 2015; Ratcliff, 1978, 1980, 2006). Under “free response,” an observer is free to set the time of response, while in the “interrogation” condition the observer does not have control over the time of the response or the duration of stimulus viewing. As implemented here, in the interrogation condition the observer must monitor the stimulus until it clears, at which point the observer can respond. The interrogation condition and variants have been extensively used in the study of evidence accumulation and decision-making mechanisms, including through the modeling of choices (e.g., Dosher, 1976, 1982; McElree & Dosher, 1989; Meyer et al., 1988; Ratcliff, 1980, 1988, 2006; Reed, 1973; Schouten & Bekker, 1967; Usher & McClelland, 2001; Wickelgren, 1977). Here, we use this condition to explore the mechanisms responsible for, and aim to model, confidence judgments (Rosenbaum et al., 2022). Note that we define “response time” in the interrogation condition to be the time from the onset of the stimulus to the response (Meyer et al., 1988).

We consider 10 models that are all variants of a core model. The core model has as its central assumption the idea from the DDM (Ratcliff & McKoon, 2008) that observers track the total difference in evidence between alternatives. In the interrogation condition, the observer does not need to set a criterion for stopping evidence accumulation and responding, as the end of the trial is determined for them. Performance can be maximized by observing and using all information presented: The observer should simply pick the option favored by all presented evidence (Figure 2A; Bogacz et al., 2006). To be precise, we assume that evidence accumulation lasts for precisely the same amount of time as the stimulus is presented for, with the final accumulator state both determining responses and being used for the readout of confidence (described below).

**Figure 1:**
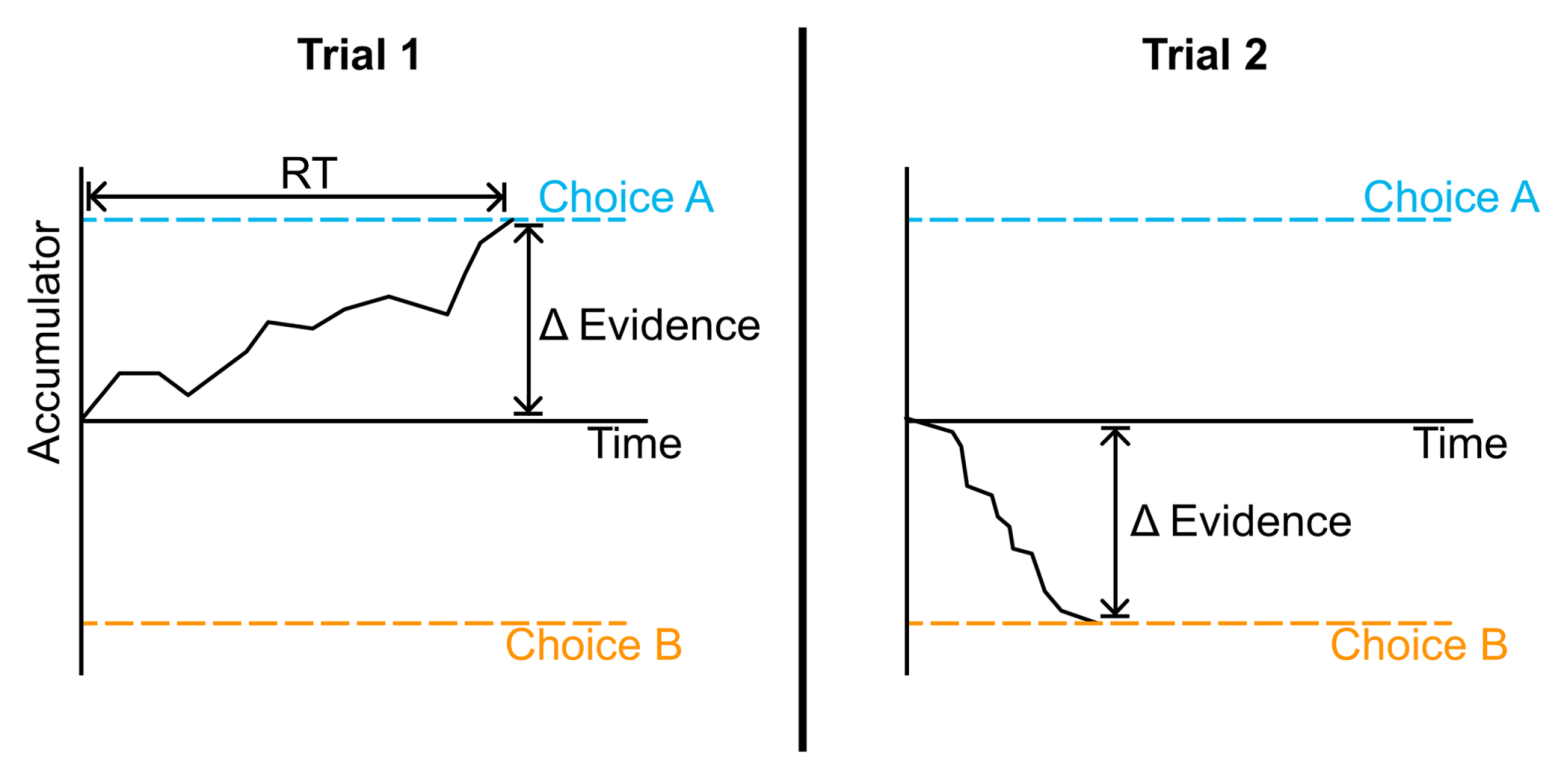
In the Drift Diffusion Model (Ratcliff & McKoon, 2008), the Observer Accumulates the Difference in Evidence Samples for Two Options *Note*. Two example trials are shown (“Trial 1” and “Trial 2”). When the difference in evidence reaches a threshold value, a response is triggered. Due to the criterion used for triggering a response, observers end every trial with the same difference in evidence between the chosen and unchosen alternative (Yeung & Summerfield, 2014). RT = response time. See the online article for the color version of this figure.

**Figure 2:**
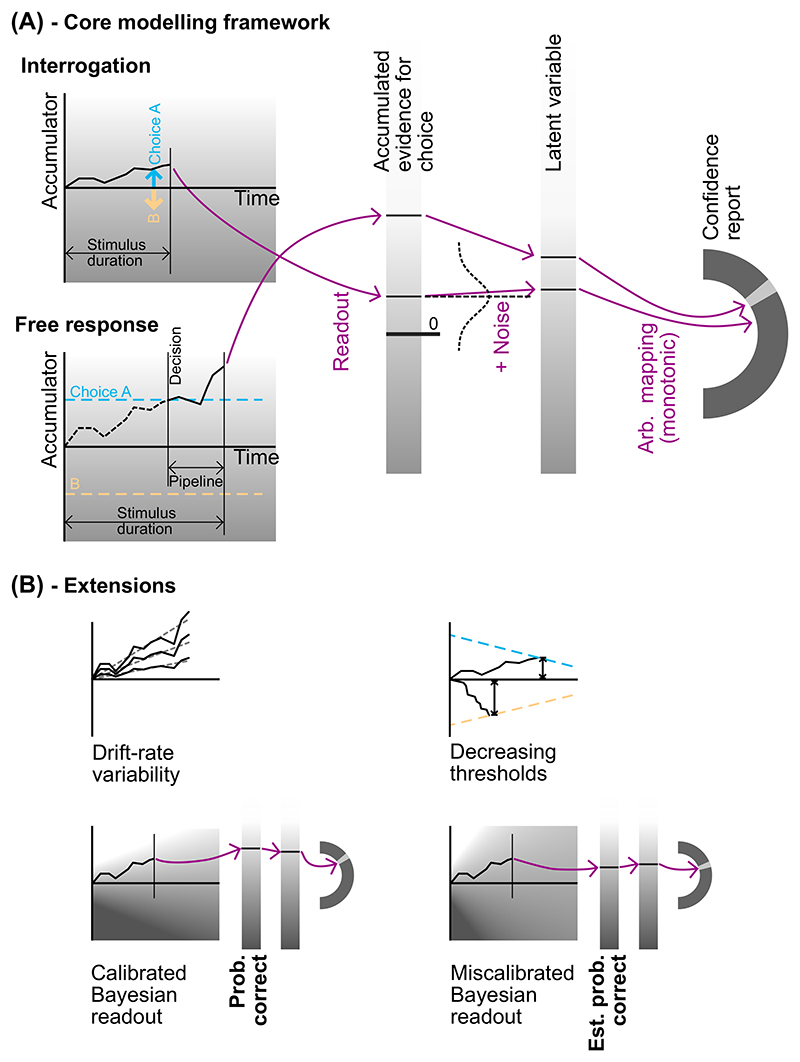
The Core Modelling Framework and Considered Extensions *Note*. (A) All models considered are built from a core modeling framework in which observers track the total difference in evidence between alternatives (Bogacz et al., 2006). When the researcher sets the time of the response (interrogation condition), observers accumulate evidence until all information from the stimulus is processed (Bogacz et al., 2006). When observers set the time of response (free response condition), observers accumulate evidence until the accumulator reaches one of two decision thresholds (Ratcliff & McKoon, 2008). Evidence accumulation continues for a short time after a decision, as sensory and motor processing pipelines mean there will be additional information that did not contribute to the decision (Resulaj et al., 2009). (B) Model variants are constructed by adding combinations of possible extensions to the core model. Bayesian confidence—that is, the probability of being correct—is a function of time spent accumulating evidence and the amount of evidence accumulated (as represented by the shading). To be precise, in the Bayesian confidence models, the observer does not read out probability correct, but a monotonic function of this, as described in the main text. The function of time and evidence used by a miscalibrated Bayesian observer to estimate the probability of being correct differs from the function that would be used by a calibrated Bayesian observer. arb. = arbitrary; prob. = probability; est. = estimated. See the online article for the color version of this figure.

In the free response condition, the observer decides themselves when to respond. Given this flexibility, they must use some policy to determine when to stop accumulating evidence and make a decision. Following the DDM, we assume the observer sets thresholds on the accumulated evidence, one for each response option (Figure 1). A response is triggered when the accumulator reaches either threshold. There is some lag between stimulus presentation and the time that the corresponding information enters the observer’s evidence accumulation (Resulaj et al., 2009). This corresponds to the time that sensory processing of incoming information takes. Similarly, there is some lag between the decision at the time of threshold crossing and the motor response to indicate the decision (Resulaj et al., 2009). This corresponds to the time that motor processing takes. Due to sensory processing delays, at the time of the decision some information that has already been presented in the stimulus is undergoing sensory processing and has not yet contributed to the evidence accumulation (Resulaj et al., 2009). Similarly, due to motor processing delays, additional sensory information will be received in the time between commitment to a decision and the motor response. We use the term “pipeline” evidence to refer to all information that is either undergoing stimulus processing at the time of the decision, or which is received during the motor processing delay. “Pre-decision” evidence refers to evidence that has entered the accumulation prior to the time of the decision (and therefore contributed to that decision). Although pipeline evidence will not contribute to the initial decision (Ratcliff & McKoon, 2008; Resulaj et al., 2009; van den Berg, Anandalingam, et al., 2016), it will be processed immediately following the decision and can therefore inform any subsequent confidence report that is given (Figure 2A; Moran et al., 2015; van den Berg, Anandalingam, et al., 2016). (We will use the fact that pipeline evidence does not influence the decision to estimate the duration of the sensory and motor processing delays, i.e., the duration of the “pipeline,” and from this we will be able to divide up the evidence presented in the stimulus into predecision and pipeline evidence; Experimental Method section.) Note that even if the stimulus terminates at the time of the response, if confidence reports are collected later, confidence will be informed by more information than the response. Namely it will be additionally informed by the pipeline evidence.

In neither of the conditions that we consider is there any time pressure on the confidence report: Participants have as long as they wish to report their confidence. Furthermore, in the free response condition, the stimulus is cleared at the time of a response, whereas in the interrogation condition, the stimulus clears before the participant is permitted to respond. Hence in both cases, the stimulus information available to the participant to inform their confidence report is fixed while confidence is being reported. In this situation, and with no time pressure on the confidence report, the normative strategy is to fully process all information that was received prior to the end of the stimulus (including all pipeline information; Bogacz et al., 2006; Moran et al., 2015) and base the confidence report on this fixed pool of information. This means that in the free response condition, just as in the interrogation condition, evidence accumulation lasts for exactly the same amount of time as the stimulus duration, with the final state of the accumulator being used to determine confidence. For example, if it takes 400 ms for information to pass through sensory and motor processing pipelines, and the stimulus clears on response, information presented in the stimulus in the 400 ms prior to response will not have been processed. Therefore, in the free response condition, evidence accumulation continues for 400 ms following the crossing of the decision threshold, before confidence is read out (Figure 2A).

On the basis of this parsimonious assumption, which requires no further parameters to be added governing the postdecision accumulation process, we are already able to make completely precise predictions for the confidence reports themselves. The time spent accumulating evidence following a decision and prior to a confidence report has itself been investigated (Baranski & Petrusic, 1998; Chen & Rahnev, 2023; Moran et al., 2015; Pleskac & Busemeyer, 2010), however, here we focus solely on the confidence reports rather than the time taken to make them. Our question is whether the DDM can be extended to account for detailed quantitative and qualitative patterns in confidence reports. This focus was motivated by a number of factors, that we detail in General Discussion section.

In the core model, confidence is based on a readout of the final state of the accumulator that tracks the difference in evidence between the two choices (Pleskac & Busemeyer, 2010; Vickers, 1979). In all models, we allow the possibility that the readout is corrupted by metacognitive noise (Bang et al., 2019; De Martino et al., 2013; Maniscalco & Lau, 2012; van den Berg et al., 2017). We assume normally distributed metacognitive noise corrupts the value of the accumulator as it is read out (note that normally distributed metacognitive noise is likely to only approximate the true form of metacognitive noise; Shekhar & Rahnev, 2021). Additionally, a subset of confidence reports are assumed to result from “lapses.” That is, instead of being generated by the usual mechanism, a random confidence report is given (further details in Modeling Method section).

In all analyses, we treat confidence as an ordinal variable (Yu et al., 2015), and only study the ordering within-participants. The great strength in this approach is that we do not have to make any assumptions about how observers use confidence reporting scales, or whether they scale, shift, or stretch their readout before reporting confidence (Ais et al., 2016; Aitchison et al., 2015; Festinger, 1943; Vickers & Packer, 1982), which seems to vary from person to person and from context to context (Ais et al., 2016; Vickers & Packer, 1982). Instead, we simply assume that the noisy internal confidence readout is mapped monotonically to the confidence report given (Figure 2; Aitchison et al., 2015). No further assumptions about the form of this mapping need to be made. While this is certainly a strength, it does mean that we will not be able to study the mapping between observers’ estimates of probability, and objective probability (Lichtenstein & Fischhoff, 1977). Furthermore, by definition, we are side-stepping important but complex questions regarding between-participant differences in average confidence (Ais et al., 2016; Pallier et al., 2002), the role of confidence in social contexts (Hertz et al., 2017), and how observers set confidence criteria (Charles et al., 2020). Instead, by studying only the ordering of confidence reports, we can focus in great detail on the perceptual component of confidence.

The core model, based on the DDM, struggles to account for confidence reports for the reasons described above. Principally, with a drift rate of fixed magnitude (but unknown sign), flat decision thresholds, and a confidence readout that directly reflects the strength of accumulated evidence, the model has no way of accounting for the relationship between confidence and response time (Pleskac & Busemeyer, 2010). We consider three features that could be added to the core model to better account for observed patterns in human confidence reports (Figure 2B): drift-rate variability (as in the 2DSD model), decreasing decision thresholds, and time penalties for confidence.

The first feature is drift-rate variability (Introduction section; Pleskac & Busemeyer, 2010; Ratcliff et al., 1999). A subtle but important point mentioned in Introduction section is that drift-rate variability can arise either because stimulus evidence strength varies from trial-to-trial or because there is trial-to-trial variability in the quality of processing constant evidence strength stimuli (Moran, 2015; Ratcliff et al., 1999, 2016). Throughout, we focus on the case in which stimulus evidence strength does not vary on a trial-by-trial basis, because this provides the greatest challenge for models of confidence (Introduction section). In this situation the only possible source of drift-rate variability is trial-to-trial variability in the quality of information processing. To capture the idea that trial-to-trial quality of information processing varies, such that the effect of presented evidence on the accumulator varies, we included a variable that we term “drift-rate scaling.” This variable changes from trial-to-trial and has a multiplicative effect on the strength of the relationship between evidence presented and changes in the accumulator (Appendix A and Calder-Travis et al., 2023). The driftrate scaling follows a normal distribution. When the drift-rate scaling is 1 (mean value), the effect of evidence on the accumulator is at its average level. When the drift-rate scaling is higher (or lower), evidence presented in the stimulus has a larger (or smaller) effect on the evidence accumulation.

The second feature that we used to extend the core model is decision thresholds that are not constant but rather decrease over time (Figure 2B; Drugowitsch et al., 2012). Decreasing decision thresholds can generate clear relationships between confidence and time for the reasons discussed in Introduction section.

The third feature considered is that, instead of confidence reflecting simply the state of the accumulator, confidence is a Bayesian readout of the probability of being correct (Figure 2B; Kiani et al., 2014; Pew, 1969; Vickers & Packer, 1982). That is, using all the information they have gathered, the observer infers the probability that they made the correct response and uses this to determine their level of confidence. As discussed in Introduction section, a Bayesian readout prescribes a specific relationship between time, evidence, and confidence and specifically features a time penalty for confidence when the difficulty of the task is unknown to the observer (Moran, 2015; Moreno-Bote, 2010). Time is taken into account because the average rate at which evidence has accumulated conveys information about the difficulty of the task. For the task we consider below, the readout takes the following form (Appendix A and Calder-Travis et al., 2023):

(1) $$\frac{X}{1-\gamma +\gamma t}.$$

This quantity is monotonically related to the probability of being correct and hence completely determines the ordering of confidence reports. Here *t* is time spent accumulating evidence, while *X* represents the evidence accumulated for the choice made. γ is a parameter that determines to what extent decision time reduces confidence. γ takes a value between 0 and 1 that is determined by Bayes rule and reflects the observer’s estimate of the magnitude of various sources of variability (Appendix A). In particular, it reflects the level of drift-rate variability compared to a weighted sum of accumulator noise, stimulus variability, and drift-rate variability. An important case is when the observer believes there is no trial-to-trial variability in drift rate—neither from trial-to-trial variability in stimulus evidence strength nor from variable processing of constant evidence strength stimuli—and therefore they believe they know the difficulty of the task. In this situation, the rate of evidence accumulation conveys no information about difficulty by definition, time spent accumulating evidence is therefore irrelevant, γ = 0, the readout in Equation 1 no longer depends on time, and the time penalty for confidence does not apply. Instead, the readout becomes identical to a readout of the state of the accumulator (when confidence is treated as an ordinal variable; Bogacz et al., 2006; Moran, 2015).

There are some further details regarding the readout (Equation 1) that are helpful to note. First, the readout is the same regardless of whether observers set the time spent accumulating evidence through their response time (i.e., free response), or whether time spent accumulating evidence is set by the experimenter (i.e., interrogation; Introduction section; Calder-Travis et al., 2023; Moreno-Bote, 2010). Because the rate at which evidence has accumulated provides information about task difficulty, accumulation time is relevant for inferring task difficulty even when accumulation time is not determined by the observer (Introduction section). Second, the specific divisive form of the readout is prescribed by the observer’s beliefs about the statistics of the task and noise corrupting incoming evidence, together with Bayes rule (Appendix A and Calder-Travis et al., 2023). Heuristic readouts utilizing a different form for the time penalty—for example, a subtractive penalty—could be designed to give plausible confidence reports. In the specific case of a subtractive penalty, after a long time spent deliberating the penalty would grow to become greater than the accumulated evidence itself (at least in the free response condition where accumulated evidence cannot grow past the decision thresholds). In this case, the penalty would outweigh the evidence, leading to a strong negative evaluation of any choice made, and a paradoxical situation in which observers would make choices they believe to be wrong. Therefore, some limit to the size of a subtractive penalty would need to be built in. Note that such a limit is automatically built into a divisive penalty: After lengthy deliberation, time, *t*, in Equation 1 is very large, so accumulated evidence, *X*, is divided by a very large number. This produces a very small but positive number, implying negligible support for the choice made (i.e., a guess), rather than negative support for the choice (i.e., suggesting an error). Differentiating between a sophisticated heuristic that approximates an optimal readout and a Bayesian readout itself is beyond the scope of this work, as we acknowledge in General Discussion section.

We consider two variants on the Bayesian readout, a calibrated and a miscalibrated readout (Drugowitsch et al., 2014). For a calibrated Bayesian observer, γ reflects the true magnitude of the various different sources of variability mentioned. A miscalibrated observer is not assumed to have perfect knowledge of the statistics for these different sources of variability. As a result, γ differs from the value used by a calibrated Bayesian observer, and the dependence of the confidence readout on evidence and time is altered. In particular, the strength of the time penalty for confidence is altered. The idea of incorrect variability estimates is consistent with the finding that human observers are poor at learning and dealing with noise associated with stimulus variability (de Gardelle & Mamassian, 2015; Herce Castañón et al., 2019; Zylberberg et al., 2014, 2016) and with the fact that in the task we use, we do not provide trial-by-trial feedback, preventing observers from calibrating their estimates. In the case of Bayesian confidence, both calibrated and miscalibrated, we assume normally distributed metacognitive noise corrupts the observer’s readout (Appendix A).

Although miscalibrated, the confidence readout remains a “Bayesian” readout here in the crucial sense that the calculations performed by this observer are exactly those performed by the calibrated Bayesian observer. Hence, the algorithm and implementation used for both the calibrated and miscalibrated readout will be identical—the two differ only in whether their representation of the task context is perfectly accurate (calibrated) or not. In general, Bayesian observers need not be omniscient observers, in that they may not immediately know the statistics of all the tasks that they perform. Like any observer, the Bayesian observer requires the opportunity to learn the statistics of a new task, and in situations in which feedback is limited, they will necessarily have to rely on their estimates for task statistics, which may well deviate from the true values. In any case, we are not strongly committed to the view that confidence itself is normative. As discussed in Introduction section, there are principled reasons for expecting decisions to be produced through normative mechanisms, even if confidence reports are not. We are motivated by the theoretical arguments for normative decision mechanisms to explore DDM-based models of confidence, including plausible variants of Bayesian confidence.

We constructed a range of models by combining the core model with different combinations of these features. We considered all possible combinations of the features, which led to 2 (drift-rate variability yes or no) × 2 (flat or decreasing decision thresholds) × 3 (confidence reflects accumulator or calibrated Bayesian readout or miscalibrated Bayesian readout) = 12 models. A calibrated Bayesian observer correctly believes there is no drift-rate variability when it is absent. As discussed, in this case γ = 0, so the time penalty for confidence does not apply. Instead, confidence reflects a simple readout of the state of the evidence accumulator. Consequently, we removed the two Bayesian models that exactly duplicated the predictions of the corresponding non-Bayesian models. This left 10 models (Table 1).

The core model (Model 0) is a baseline DDM that we expect to struggle to account for confidence reports (Table 1). It is included for comparison. Model V is closely related to the 2DSD model (interrogation version; Pleskac & Busemeyer, 2010), which struggles to capture the strength of the relationship between confidence and response time (Pleskac & Busemeyer, 2010). For all models, we do not consider trial-to-trial variability in the start point of the accumulator, a source of variability considered by Pleskac and Busemeyer (2010). Start point variability is usually justified on the grounds that it permits models to account for certain patterns in response times (Ratcliff et al., 2016). Because our main focus was understanding confidence, and to keep the models as constrained as possible, we did not consider this additional complexity (when comparing Model V and 2DSD, note also the different operationalization of drift-rate variability discussed above). Models D–VDM (in Table 1) represent different combinations of possible extensions that may allow the DDM to account for confidence reports, and in particular, their relationship with response time.

### Explaining Key Findings

There are a range of phenomena that a satisfactory account of decisions and confidence would need to account for, many of which were set out as “empirical hurdles” by Pleskac and Busemeyer (2010). In this section, we explore key explanations for known phenomena relating to confidence, explicitly linking these explanations to the model features on which they rely (Tables 2 and 3; because our focus is confidence, we do not consider phenomena relating solely to responses and response times, but see Pleskac and Busemeyer, 2010, for explanations of how such effects can be accounted for within a DDM framework). Where a phenomenon applies to both free response and interrogation conditions, we only discuss the explanation for this phenomenon in the free response condition: It is often the presence of decision bounds that make observed phenomena difficult to account for. Hence, we will use explanations involving decision thresholds, and the idea that under free response conditions evidence is in sensory and motor processing pipelines at the time of response, which is a feature of all of the models we consider.

Pattern (A) “conf. with signal” in Table 2 is that confidence increases with the strength of signal provided by the stimulus. A stronger signal leads to a higher drift rate and hence, on average, a greater amount of evidence in the pipeline favoring the correct response, and increased confidence (Pleskac & Busemeyer, 2010). The time penalty for confidence will amplify the effect because higher drift leads to faster responses and, as a result, higher confidence. Similarly, decreasing decision thresholds may exacerbate the effect because faster responses are generated when the decision threshold is reached sooner, and hence, these decisions will be made with a greater balance of evidence in their favor.

Pattern (B) “acc. and conf.” in Table 2 is that choice accuracy and confidence are positively correlated within individuals, even when considering trials of a fixed difficulty. Pipeline evidence will, in general, support the correct option, adding further evidence to the accumulator if the correct option was chosen (Pleskac & Busemeyer, 2010). The reverse holds in the case of an incorrect choice. The result is greater confidence on correct trials, and hence, a correlation between confidence and accuracy.

Key relationships have been identified between confidence and time. For example, confidence increases with response time when comparing across conditions that differ in their emphasis on trading speed for accuracy, and in settings where stopping is enforced at a particular time such as in the interrogation condition, pattern (C) “conf. with time (speed-acc)” in Table 2. This pattern can be explained within any DDM account of confidence (Ratcliff & McKoon, 2008). In the free response condition when speed is emphasized, observers use lower decision thresholds such that less evidence is required to trigger a decision. This leads to faster, less accurate choices, but also to lower confidence. In the interrogation condition, the state of the accumulator simply grows with time as more evidence samples are received, generating an increase in confidence (provided the effect of the confidence time penalty is not too strong; Rosenbaum et al., 2022).

In contrast, in free response tasks, within a single speed-accuracy condition, confidence is higher for faster responses than slower responses, pattern (D) “conf. with time (free)” in Table 2. We can account for this pattern qualitatively using the idea of a processing pipeline together with drift-rate variability (Pleskac & Busemeyer, 2010; Introduction section): Trials with higher drift rates are associated with faster responses but also stronger evidence in favor of the chosen option in the processing pipeline, and hence increased confidence. As discussed above, Pleskac and Busemeyer (2010) noted that 2DSD—which contains a processing pipeline and drift-rate variability—struggles to explain the strength of the negative relationship between decision time and confidence. In contrast, models that include decreasing decision thresholds inherently create a strong dependency between confidence and decision time, because slower decisions are made with a smaller balance of evidence in their favor. The time penalty for confidence may also help us explain why the relationship between decision time and confidence is so strong: Observers who apply the time penalty take into account the time spent deliberating, in addition to the balance of evidence, in their confidence readout. We will see in computational modeling below, whether these features can account for the strength of the effect of response time on confidence.

Pattern (E) “conf. resolution” in Table 3 is that confidence is a stronger predictor of accuracy when task instructions and incentives emphasize speed over accuracy than vice versa. We can account for this effect if people take longer between decisions and confidence reports under speeded conditions (Pleskac & Busemeyer, 2010). The longer this interjudgment time, the greater the effect of pipeline evidence, which increases confidence on correct trials and decreases it on incorrect trials. However, we may not need to invoke an additional mechanism to explain this pattern in models featuring a time penalty for confidence. In a range of settings, the effect of the time penalty for confidence is to divide the accumulated evidence by a function of time (Drugowitsch et al., 2012; Moreno-Bote, 2010; Appendix A; Equation 1), decreasing the effect of evidence on confidence. At slow response times, the time penalty will be greater, decreasing the effect of pipeline evidence on confidence further, and therefore decreasing the difference in confidence between correct and error trials.

In addition to the patterns identified by Pleskac and Busemeyer (2010), with the idea of pipeline evidence, the time penalty for confidence, and a flat decision threshold, we can explain some results that have previously appeared difficult to reconcile. Sanders et al. (2016) observed that confidence in errors decreases as a task becomes easier and stimuli more discriminable. However, Kiani et al. (2014) found that, when confidence reports were collected simultaneously with decisions, confidence in errors increased with stimulus discriminability. These findings, pattern (F) “conf. in errors” in Table 3, can be reconciled by considering the timing of decision and confidence reports (Calder-Travis et al., 2023; Desender et al., 2020; Khalvati et al., 2020; Kiani et al., 2014). Sanders et al. (2016) used confidence reports that followed decisions, while Kiani et al. (2014) used confidence reports that were made simultaneously with decisions. If the latter setup eliminates the accumulation of pipeline evidence following the crossing of the decision threshold, then evidence accumulation will always end at the decision threshold, and the final accumulated difference in evidence will always be the same. On the other hand, decision time will vary between trials. Increased signal strength leads to faster correct responses, but crucially, it also leads to faster error responses (when the decision threshold is flat, and without internally generated variability in drift rate; Shadlen et al., 2006). Faster responses are associated with a reduced time penalty for confidence. Hence, in the absence of pipeline evidence, confidence in errors will be greater when a task is easier (Calder-Travis et al., 2023; Desender et al., 2020). When confidence reports follow a decision, and pipeline evidence is accumulated, pipeline evidence generally favors the correct option, reducing confidence in errors. When signal strength is greatest, this effect is strongest, leading to lower confidence in errors when a task is easier.

A further distinctive finding was presented by Yu et al. (2015). Yu et al. (2015) manipulated the time between the primary decision and confidence report, finding that confidence on correct trials was relatively unaffected by this interjudgment time, whereas confidence on error trials decreased, pattern (G) “conf. with IJT” in Table 3. Yu et al. (2015) explained this pattern by invoking the idea of a nonnormative leak in the evidence accumulation mechanism, such that there is a continuous decay or loss of previously accumulated evidence (Miletic et al., 2016; Ratcliff & Smith, 2004; Usher & McClelland, 2001). However, it may be possible to account for the effects observed by Yu et al. (2015) in a different way. Specifically, it may be possible using DDM-based models (implying no leak of accumulated evidence), coupled with a Bayesian time penalty for confidence. On correct trials, evidence gathered during the interjudgment time will generally support the choice made, meaning confidence will increase with interjudgment time. However this effect will be opposed by the time penalty for confidence, which decreases confidence with the time spent accumulating evidence. On error trials, evidence gathered during the interjudgment time will generally conflict with the choice made, decreasing confidence with interjudgment time. This effect will additionally be enhanced by the time penalty for confidence.

### Predicted Pattern

Although it is important to account for benchmark findings, and findings that have been difficult to reconcile, a stronger test is whether a model predicts qualitative patterns in new data. In this section, we note a new pattern predicted by models in which decisions and confidence both result from the same evidence accumulation, and where a decision threshold triggers a response.

The novel prediction we add to our list of confidence-related patterns applies specifically in the free response condition. It is that, once any effect of response time has been accounted for, evidence from different time periods during a decision will weigh differently on participants’ confidence judgments, Pattern (H) “evidence on conf.,” Table 3. Specifically, we predict that predecision evidence will have a much smaller effect on confidence than pipeline evidence. This key prediction is initially counterintuitive given that all of the models we consider treat overall accumulated evidence as the basis for confidence judgments. However, the prediction follows directly from the assumptions that decisions and confidence are the result of the same evidence accumulation process, and that in free response tasks a decision threshold is used. Under these simple assumptions, the state of the accumulator at the time of the decision is fully determined by the shape of the decision threshold. As such, evidence processed prior to a decision (i.e., all evidence apart from pipeline evidence) is irrelevant to confidence once the time of the decision is taken into account. By contrast, pipeline evidence should affect confidence independent of decision time. This distinction between evidence accumulated early versus late in the decision process does not hold in the interrogation condition, where we hypothesize that observers do not use decision thresholds. Instead observers make a decision after stimulus presentation ends and all evidence has been processed. In this case the decision time does not provide information about the state of the accumulator, and there is no pipeline evidence gathered following a decision. Therefore, in this case all evidence will be equally strongly related to confidence.

### Four Tests

Using our knowledge of the various patterns and explanations in Tables 2 and 3, we next aimed to identify quantitative tests of our core modeling assumptions. Our core modeling assumptions—that is, those assumptions shared by the core model and all its variants (Table 1)—were that (a) confidence reflects a (possibly Bayesian) readout of the evidence accumulated, (b) the same DDM evidence accumulator used for the decision is used to read out confidence, and (c) a decision threshold is used in the free response condition, but not in the interrogation condition. From Tables 2 and 3 we see that our core modeling assumptions alone can generate patterns (C) “conf. with time (speed-acc)” and (H) “evidence on conf.” Using this knowledge, we can specify specific tests that can be applied to new data that if verified would support our core modeling assumptions and if falsified would lead us to question those core assumptions.

Before setting out specific tests, we note that although not predicted by any single core modeling assumption, pattern (D) “conf. with time (free)” can be generated by each of the features used to build variants of the core model (drift-rate variability, time penalty for confidence, decreasing decision thresholds). Hence, this pattern can be used to build tests of the general set of extensions. An important detail is that the core model itself does not generate pattern (D) “conf. with time (free),” because none of the extensions are present in the core model.

Having noted specific patterns produced by models featuring our core computational assumptions, we use those patterns to predict the following specific results, which can be used as tests of the core modeling assumptions:

- Test I: In the free response condition, confidence will be negatively related to response time, pattern (D) “conf. with time (free)”.
- Test II: Confidence will be more negatively related to response time under free response, than in the interrogation condition, pattern (C) “conf. with time (speedacc)” and (D) “conf. with time (free).”
- Test III: Under free response, once the effect of response time has been accounted for, the effect of predecision evidence on confidence will be smaller than the effect of evidence gathered after a decision, pattern (H) “evidence on conf.”
- Test IV: Once the effect of response time has been accounted for, the effect of predecision evidence on confidence will be stronger in the interrogation condition than in the free response condition, pattern (H) “evidence on conf.”

As discussed above, all model variants are underpinned by the same core assumptions and therefore all predict these four specific results. The only exception is the core model (Model 0), which cannot explain pattern (D) “conf. with time (free)” (Table 2) and hence does not predict Test I. Nevertheless, the core model predicts that the relationship between time and confidence will be smaller (and hence “more negative”) in the free response condition, due to the presence of decision thresholds in this case, and their absence in the interrogation condition. Tests I and II provide basic proofs-of-concept for our computational modeling approach because, as discussed, the associated patterns have repeatedly been observed before and should follow naturally from our core modeling assumptions. Nevertheless, if these tests failed in a new data set, it would provide evidence against those assumptions. Tests III and IV thus provide the critical test for our modeling approach, in terms of a novel prediction about key qualitative features of the relationship between decision evidence and decision confidence. To anticipate our results, all four qualitative tests were satisfied in our data. As detailed in later sections, these affirmative qualitative results allowed us to proceed to an exploration of the precise quantitative predictions of the 10 model variants.

## Experimental Method

We have considered a core DDM, and nine variants of this model featuring combinations of drift-rate variability, decreasing decision thresholds, and a (possibly miscalibrated) Bayesian time penalty for confidence. We sought to apply all four qualitative tests of the nine variants of the core model (Tests I–IV, Models section) in a preregistered, adequately powered study. As the models make contrasting predictions for the interrogation and free response conditions (Models section), and patterns in human confidence data are also known to differ strongly (Rosenbaum et al., 2022), using both conditions in the same experiment can provide a particularly strong test of the models (Ratcliff, 2006). We will capitalize on this in later sections with computational modeling, and ask whether any model variant can simultaneously account for the precise quantitative patterns observed in the two conditions, in addition to the qualitative patterns.

### Participants

Forty-nine participants were recruited to take part in the study (36 female, 13 male; 39 aged 18–25, 10 aged 26–35). Consistent with a preregistered recruitment schedule (see below), only data from the first 48 participants were analyzed. The study was approved by the appropriate university ethical review body (Oxford University Medical Sciences Interdivisional Research Ethics Committee reference R56149/RE001), and all participants gave informed consent.

### Apparatus

A cathode ray tube monitor at 60 Hz refresh rate, a resolution of 1,600 × 1,200, and physical viewable size 36.4 × 27.3 cm was used to present stimuli. A windows PC running Psychtoolbox-3 on MATLAB was used for the experiment (Brainard, 1997; Kleiner et al., 2007; Pelli, 1997).

### Stimuli

Stimuli were two circular arrays of dots, one presented 2.9 degrees of visual angle (DVA) to the left and one 2.9 DVA to the right of the center of the screen (assuming stimuli were viewed at approximately 60 cm). The arrays had no outline but had an imaginary circular boundary at a radius of 2.0 DVA. Within this imaginary circle, 3,096 nonoverlapping dot locations were defined. Within a trial, dots appeared in a new subset of these locations every 50 ms “frame” (Ratcliff & Smith, 2010). The number of dots in each array varied around a fixed mean value such that either the left or right array had more dots on average. Fluctuations across frames in the number of dots presented provided a time-varying signal that could be correlated with participants’ decisions and reported confidence (Carlebach & Yeung, 2020; Charles & Yeung, 2019; Resulaj et al., 2009; Shadlen & Newsome, 2001; van den Berg, Anandalingam, et al., 2016). Each dot was square, 0.043 DVA in height, and all dots were separated from their neighbors by 0.022 DVA. Dots and the background were gray scale with RGB color value 200 and 80, respectively.

### Procedure

**Figure 3:**
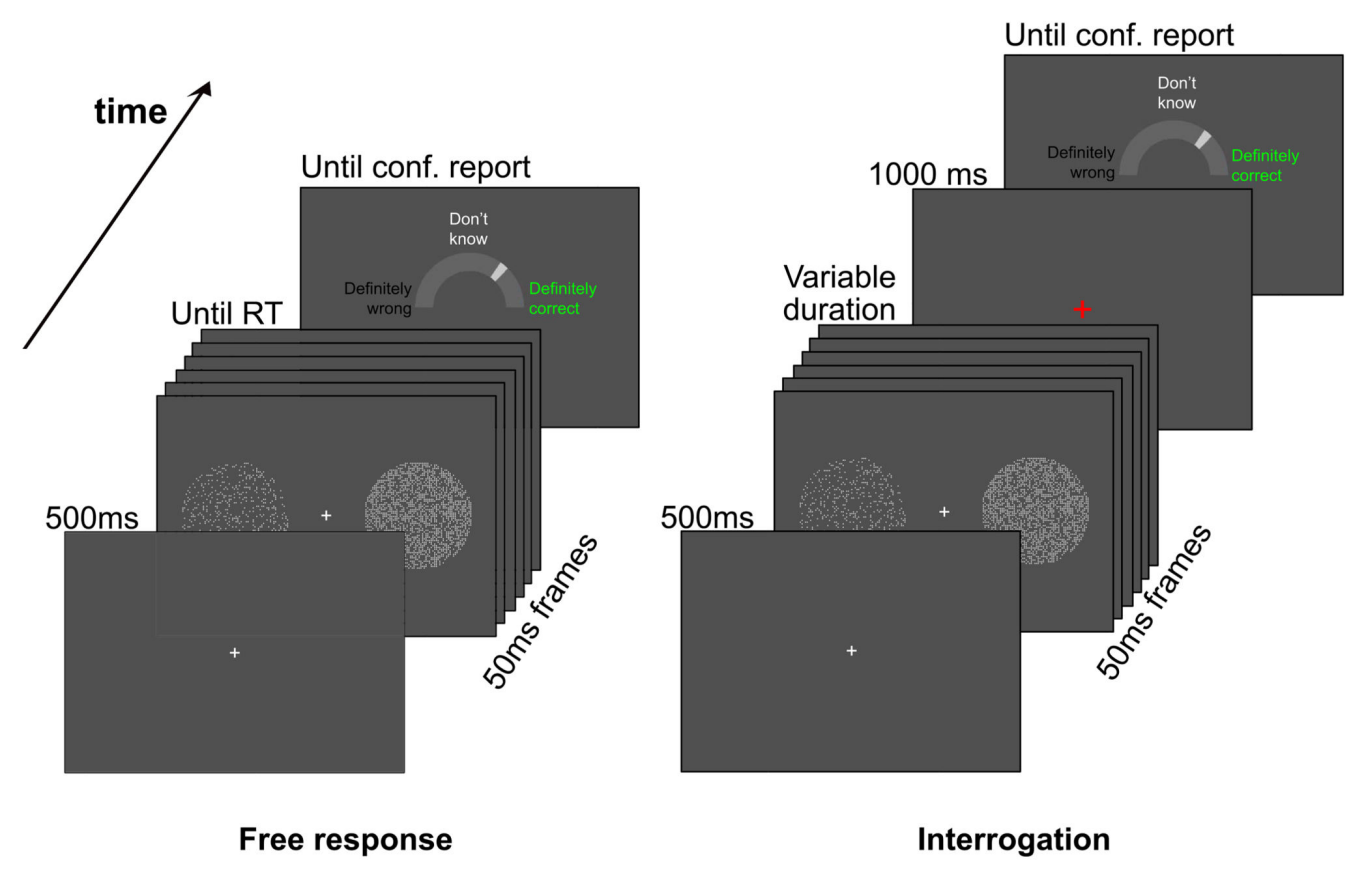
Participants’ Task Was to Determine Which Array Contained More Dots on Average *Note*. The number of dots changed every 50 ms, resampled from two independent truncated normal distributions, one for each array. (“Free response”) In the free response condition, participants could respond when they liked. (“Interrogation”) In the interrogation condition participants had to respond within 1 s of a red cross appearing that marked the disappearance of the dot arrays. conf. = confidence; RT = response time. See the online article for the color version of this figure.

On each trial, participants’ task was to determine which of two arrays contained the most dots on average. Participants used the left or right mouse button to indicate their response, before reporting their confidence (throughout “response” refers to the left vs. right choice, not the confidence judgment). The study used both free response trials (participants can respond when ready) and interrogation trials (participants cued when to respond; Models section; Bogacz et al., 2006), with a blocked within-participants design (Figure 3).

In the free response condition participants were asked to be “as accurate and fast as possible.” After a response, the stimulus cleared (unless the response was before the stimuli appeared, in which case a warning message was displayed for 6 s and the trial ended). In the interrogation condition, participants had to wait for the stimuli to disappear, at which time the fixation cross would turn from white to red, and they could respond. They had 1 s to respond, or they would receive a message, “Too slow” and the trial would end after a further 2 s. If they responded before the stimuli disappeared they would receive a message, “Too early,” and the trial would end after a further 2 s. The time at which the stimuli disappeared was random and drawn from a truncated normal distribution. This was designed to approximately match response times in the interrogation condition to those in the free response condition, minimizing the risk that participants would only monitor interrogation condition stimuli for a limited period of time (Balsdon et al., 2020; Ratcliff, 1988, 2006). Specifically, prior to truncation, the normal distribution had a mean and standard deviation matched to the mean and standard deviation in the most recent free response block (and *M* = 750 ms, *SD* = 400 ms on the very first block). The distribution was truncated at 200 ms and 4,000 ms. Each block of the experiment only contained trials from one of the two conditions, and participants were informed of the condition before the start of each block.

The number of dots in the two arrays was resampled every 50 ms from two independent truncated normal distributions, one for each array. On each trial, prior to truncation, one of the normal distributions had a mean 90 dots higher than the mean of the other distribution. Each distribution had a standard deviation of 220 dots. These distributions were then truncated to ensure that the number of dots was always positive and always below or equal to the maximum number of dots that could be displayed in an array at once (3,096). The half-way point between the means of the two distributions was itself randomly and independently set on a trial-by-trial basis, to reduce the risk that participants would focus on a single array of dots (Charles & Yeung, 2019). The half-way point was drawn from a truncated normal. Prior to truncation, it had a mean of 1,000 dots and standard deviation of 100 dots. The distribution was truncated to be between 500 and 1,500 dots. In each block, there was an equal number of trials in which the correct answer was left and right, and the order was randomly shuffled.

Note that some trials were more difficult than others because of frame-by-frame variability in evidence strength present in the stimulus used. However, aside from minor truncation effects, there was no trial-by-trial variability in evidence strength (trial-by-trial variability is a source of variability that has a consistent effect over the whole duration of a trial). As discussed in the introduction, when trial-by-trial variability in stimulus evidence strength is present, this generates trial-by-trial variability in drift rate, making some patterns in confidence data easier to account for. We focus throughout on the more challenging case of no trial-by-trial variability in stimulus evidence strength.

After each legitimate response (i.e., the response did not incur a too slow/fast error message), participants were asked to report their confidence. The cursor would appear at the center of the screen, along with an arc that formed a semicircle around the cursor. Participants reported their confidence by clicking on the arc. This setup was chosen so that participants did not have to move the mouse further to report some levels of confidence than others. The left of the arc was marked “Definitely wrong,” the center marked “Don’t know,” and the right marked “Definitely correct.” Participants were asked to use the full range of the scale. There was no time pressure when reporting confidence. Following a confidence report, the next trial would begin after a further 400 ms, starting with a 500 ms presentation of a central fixation cross.

The main experiment comprised 16 blocks of 40 trials each, and participants had two blocks of training prior to this. Each successive pair of blocks comprised one free response block and one interrogation block, with condition order randomized within this pairing. Participants did not receive feedback on a trial-by-trial basis, but at the end of each block participants were informed of the number of correct responses they had made in the block, along with their high scores (maximum number of correct responses in a block) in each condition.

All manipulations performed are described above (Simmons et al., 2011). The following provides a comprehensive list of measures recorded each trial: response, response time, confidence, a complete description of the stimulus, and trial duration.

### Analysis

Our central aim was to test for the predicted dependence of confidence on decision time and the evidence received at different time points (particularly predecision vs. pipeline evidence). Our analyses of evidence strength focused on fluctuations in the number of dots around the mean of the relevant distribution, for each circular array (Charles & Yeung, 2019; Resulaj et al., 2009; Zylberberg et al., 2012). As discussed, the number of dots in each array changed every 50 ms frame, each time being resampled from a specific distribution (Figure 3). For each frame and array, we looked at the number of dots presented, after subtracting the mean of the distribution from which this number was sampled (which was fixed across frames). Then, for each frame, we subtracted the resulting value for the unchosen array, from the resulting value for the chosen array. We refer to this measure of evidence as the “evidence fluctuations for choice.”

**Figure 4:**
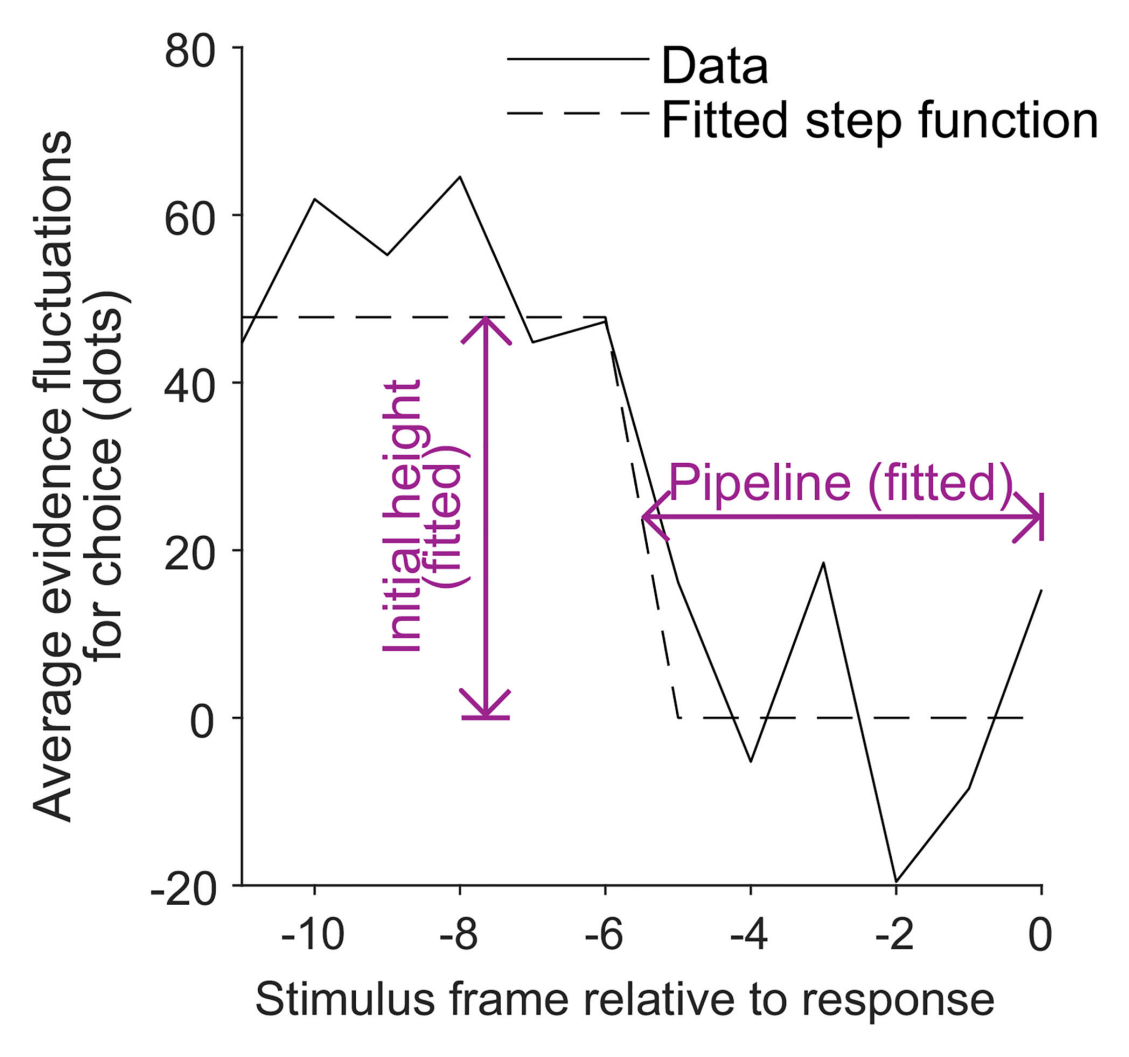
Duration of Sensory and Motor Processing Pipelines Was Estimated by Fitting a Step Function *Note*. The step function was fitted to average evidence fluctuations in the frames running up to choices in the free response condition. Approach adapted from van den Berg, Anandalingam, et al. (2016). This figure shows an example fitted step function for one participant. See the online article for the color version of this figure.

We divided evidence fluctuations into two quantities: predecision evidence, which is evidence processed before the participant made their decision (at the time of threshold crossing), and pipeline evidence, which is evidence that is still moving through sensory and motor processing pipelines at the time of the (physical) response (Resulaj et al., 2009). This definition implies that pipeline evidence will be presented close in time to the (physical) responses and will not affect the decision made (Resulaj et al., 2009; van den Berg, Anandalingam, et al., 2016). If evidence in a frame has an effect on the choice made, in general, evidence fluctuations in the frame will support that choice. Hence, average evidence fluctuations for the chosen option will be above zero. Separately for each participant, we looked at the average evidence fluctuations for the chosen option in the 12 frames preceding responses. Adapting the approach taken by van den Berg, Anandalingam, et al. (2016), we fit a step function to the average evidence fluctuations. We fit the initial value of the function (in the 12th frame prior to response), and the frame in which the function dropped down to zero (constrained to fall within 1–10 frames, inclusive, prior to response) by minimizing the root-mean-square error (Figure 4). The frame in which the step function drops provides an estimate of the number of stimulus frames immediately prior to a response that do not inform the decision but are in sensory and motor processing pipelines. Predecision evidence and pipeline evidence on each trial were calculated by summing the fluctuations in the relevant frames.

We only used data from the free response condition to estimate the number of frames in processing pipelines. In the interrogation condition, if the manipulation is successful, decisions are made after the stimulus has cleared from the screen. Hence, no extra evidence is gathered between the time of the decision and the response. Nevertheless, we can analyze the impact of evidence from the corresponding final period of stimulus presentation, using the pipeline duration estimate derived (on a participant-by-participant basis) from the free response condition. This allows us to compare across conditions and allows us to check for artifacts: In the interrogation condition, there should be no difference between effects of the predecision evidence and the “pipeline” evidence.

With measures of predecision and pipeline evidence, we next fit regression models to estimate the strength of the relationship between key variables and confidence. Precisely, we fit a probit ordinal regression model to the data (Long, 1997), with confidence reports binned into five categories as the outcome variable. We quantile binned confidence separately for each participant, and for each condition (interrogation vs. free response), with approximately equal numbers of cases in each bin. We ran the regression analysis separately for each participant and condition. Ordinal regression is very similar to logistic regression but can handle cases in which the outcome variable has several ordered categories, rather than only two categories as in logistic regression (Long, 1997). Predictors in the regression were response time, predecision evidence, pipeline evidence, and accuracy (coded “1”/“0” for correct/incorrect respectively), individually *z*-scored (intercept terms are also fit). (Specifically, the number of intercept terms is one less than the number of categories of the outcome variable, which in this case is binned confidence.) We applied the reverse transformation to the coefficients produced by the regression, to return them to the units they would have had if *z*-scoring was not applied (Appendix C). For all Tests I–IV (Models section), we verified the same results were obtained without any *z*-scoring.

We examined whether evidence fluctuations and response time predicted confidence, by performing statistical tests on the coefficients produced by the ordinal regression onto confidence. These coefficients reflect the strength of the relationship between the predictors and the outcome. As we performed an ordinal regression for each participant and condition, for each predictor this approach produced one coefficient per participant and condition. We used these coefficients in *t* tests to look for differences from zero, or other coefficients. We measured effect size using the one-sample variant of Cohen’s *d* (Cohen, 1988), computed by dividing the mean of the values being compared to zero, by the estimated population standard deviation.

We could not use trials in which no confidence report was obtained (because the response was too fast/slow). We excluded further trials and participants according to preregistered criteria: We excluded any trial in which the number of frames prior to response was less than, or equal to, the estimate of the pipeline duration for the participant (the pipeline duration estimate is the estimate of the number of frames that are in the processing pipeline at the time of responses). We excluded participants that met any of the following conditions: (a) fewer than 60% correct responses, where this is calculated using included trials; (b) prior to binning, any particular confidence value was reported on more than 30% of trials; (c) a confidence bin, after binning with MATLAB’s “quantile” function, contained <5% of included trials; (d) there were less than 70 included trials; and (e) after predictors were *z*-scored and combined into a single matrix (where each row was a case and each column a predictor), the two-norm condition number for inversion of this matrix (which provides a measure of sensitivity to errors in certain operations on the matrix) was greater than 1,000. All exclusion criteria were applied separately to both conditions, and if met in either condition, data from the participant were excluded. These criteria led to the data set from 1/48 participants being excluded from the regression analysis.

### Plotting Procedure

For data visualization in figures below that have continuous variables on the *x*-axis of plots (apart from plots of quantile probability functions and probability densities), we first binned trials according to the *x*-axis value separately for each participant and each plotted series, such that bins on the *x*-axis contained approximately equal numbers of trials. The *x*-position of a bin was determined by an average of the values falling in that bin. Specifically, the mean value of the *x*-variable in each bin, for each participant, was computed. The mean, across participants, of these means then determined the *x*-location.

The *y*-value of a bin represents the mean value of the *y*-variable across participants. Unless noted, error bars and error shading represent ±1 standard error of the mean (SEM) across participants or simulated participants. Unless noted, plots were based on the data from all participants, and all trials in which a confidence report was obtained. For all plots that refer to “binned confidence,” confidence reports were divided into four bins on a participant-by-participant basis.

In some plots, small panels (marked “P10,” “P20,” and “P30”) additionally present individual participant data for three random participants. In this case, fewer bins were used for the variables on the *x*-axis (to reduce noise), and of course no averaging was conducted across participants (plots showing across-participant averages are marked “Av” instead).

### Preregistration

The details of this analysis (excluding the computational modeling in later sections) were decided prior to data collection and recorded in a preregistration (Open Science Framework registration at https://osf.io/wzrhm). Preregistered sample size was justified with a power analysis (see preregistration; Faul et al., 2007). We preregistered four statistical tests on the coefficients produced by the ordinal regression analysis (preregistration of a test is indicated in the results section).^1^ As these tests were specified a priori we made no correction for multiple comparisons.

It should be noted that we originally made all preregistered predictions on the basis of Model D only (Table 1), but a range of models are consistent with the predictions (Models V–VDM in Table 1). We compare all models using computational modeling in later sections.

Anonymized data, experiment code, analysis code, and all modeling and simulation code written for the study will be made publicly available on publication at https://doi.org/10IO/QPSEM.

## Experimental Results

### Task Performance

Averaging across participants, 98% of trials ended with valid confidence reports, and accuracy on these trials was 77%. Average response time was 2.08 s in the free response condition, and 2.19 s (including stimulus presentation time) in the interrogation condition. Distributions over response times and confidence reports are provided in Appendix D. Using the method described in Experimental Method section, we estimated the number of 50 ms frames in sensory and motor processing pipelines at the time of response. Across participants, the median value was six frames (interquartile range, 1; 6 frames = 300 ms). We used Goodman and Kruskal’s gamma to assess the rank correlation between confidence and accuracy. Computing one value of gamma for each participant and comparing the resulting values to zero, we found that accuracy and confidence were reliably positively correlated, average Γ = 0.25, *t*(47) = 14, *p* = 3.0 × 10^−18^, *d* = 2.0. Taken together, these results suggest participants understood the task and reported a meaningful value for confidence.

### Response Time and Accuracy

**Figure 5:**
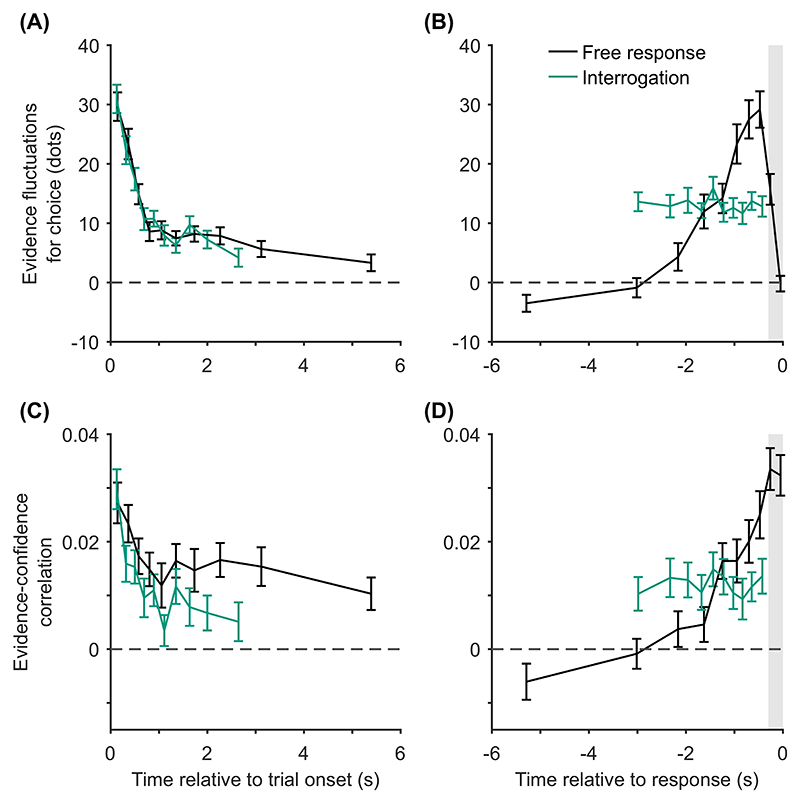
The Effect of Response Time on (A) Accuracy and (B) Confidence in the Main Study *Note*. “Response” refers to the left vs. right choice. (B) Consistent with model predictions and with previous findings (Pleskac & Busemeyer, 2010), confidence decreased with response time in the free response condition, and the relationship between time and confidence was more negative in the free response condition than in the interrogation condition. The rationale for using binned confidence is explained in Models section. Similar patterns were obtained when plotting raw confidence scores against response time. Error bars represent ±1 SEM of the mean. Plotting details in Subsection “Plotting Procedure.” SEM = standard error of the mean. See the online article for the color version of this figure.

Prior to looking at the relationship between confidence and response time, we look at the relationship between accuracy and response time. The latter will inform our interpretation of the former. Longer response times appeared to be associated with lower accuracy in the free response condition but greater accuracy in the interrogation condition (Figure 5A). To explore this pattern further, we compared mean response time in correct trials versus error trials, via *t* test on values across participants. In the interrogation condition, errors were faster than correct choices, *t*(47) = −6.0, *p* = 2.4 × 10^−7^, *d* = −0.87, whereas, in the free response condition, errors were slower than correct choices, *t*(47) = 3.6, *p* = .00078, *d* = 0.52. In the interrogation condition, if the observer pays attention throughout the course of the trial, longer response times for correct responses are expected. This is because observers receive more evidence when the stimulus is presented for a long time and will therefore be more accurate. The situation is very different in the free response condition, where the observer must set a decision threshold and determine response time themselves. Both drift-rate variability and decreasing decision thresholds can lead to errors that are slower than correct choices (Ratcliff & McKoon, 2008; Shadlen & Kiani, 2013) and hence are consistent with the observed pattern.

### Test I

Our first key prediction concerned the relationship between response time and confidence in the free response condition (Figure 5B; Models section for rationale for using binned confidence; a similar plot was obtained when plotting raw confidence instead). As predicted, in this condition confidence decreased with time (Figure 5B). For each participant, and separately for the two conditions, we ran a regression to predict confidence using predecision evidence, pipeline evidence, response time, and accuracy (Experimental Method section). To test the apparent effect of response time in the free response condition, we compared the regression coefficients for time to zero using a *t* test. Confidence was negatively related to response time, *t*(46) = −8.8, *p* = 9.7 × 10^−12^, one-tailed, *d* = −1.3, preregistered, Test I, consistent with the results from the preliminary study and previous findings (Pleskac & Busemeyer, 2010, Empirical Hurdle 4). This finding is consistent with all variants of the core model, but not the core model itself (Models section).

### Test II

In contrast, in the interrogation condition, confidence did not vary significantly as a function of response time, *t*(46) = −0.90, *p* = .37, *d* = −0.13; Figure 5B. The core model and all of its variants predict a more negative effect of response time in the free response than in the interrogation condition, because observers only use a decision threshold in the free response condition (Models section). Indeed, a paired *t* test comparing the relevant regression coefficients showed this was the case, *t*(46) = −8.1, *p* = 1.1 × 10^−10^, one-tailed, *d* = −1.2, preregistered, Test II. Nevertheless, we were surprised that confidence did not increase with response time in the interrogation condition. This result conflicts with previous findings (Pleskac & Busemeyer, 2010, Empirical Hurdle 5) and the intuition that if the stimulus is presented for longer, more evidence will be gathered, and confidence will be higher.

One explanation for this surprising result is that, in our interrogation condition, observers only monitor the stimulus for a short initial period of time, such that their decisions are largely independent of later evidence and, correspondingly, confidence is insensitive to the overall amount of information presented. This possibility is consistent with the idea that observers set “implicit” decision thresholds in interrogation tasks (Balsdon et al., 2020; Kiani et al., 2008; Ratcliff, 1988, 2006), which trigger decisions that are stored by the observer until the response cue, with evidence received following the decision ignored. In this case, evidence accumulation only continues for the full duration of the stimulus in a subset of trials. However, we carefully matched response times across the two conditions with reasonable success (Appendix D), which may have minimized the number of times implicit thresholds were reached even if they were present. Supporting this claim, there is evidence from previous research that such an experiment design can successfully ensure implicit thresholds are not used in the interrogation condition (Rosenbaum et al., 2022). Thus, other factors are likely at play in producing this surprising null relationship between confidence and response time: As we will discuss later, this qualitative pattern favors models featuring a miscalibrated Bayesian readout for confidence. In General Discussion section, we consider this lack of a significant relationship between response time and confidence in the interrogation condition in the context of previous findings.

### Effect of Evidence on Choice

**Figure 6:**
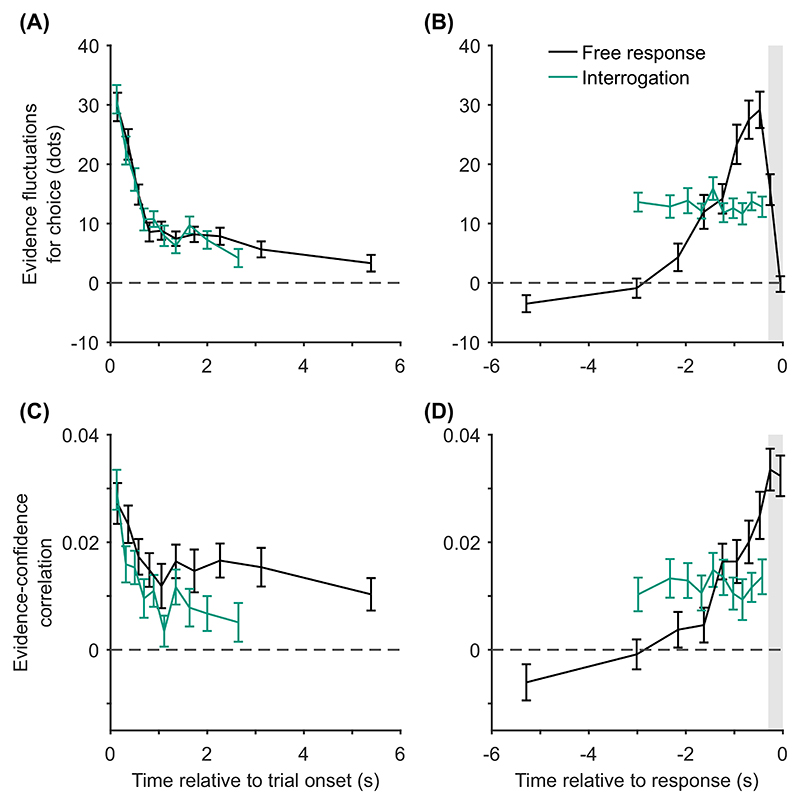
The Effect of Evidence Fluctuations on (A and B) Choices and (C and D) Confidence *Note*. “Response” time refers to the left vs. right choice. Panels A and B plot the average evidence fluctuations in the direction of the choice made. This serves as a measure of the effect of evidence on the choice made (Experimental Method section; Resulaj et al., 2009). Panels C and D plot the rank correlation (Kendall’s τ) between evidence fluctuations and confidence. The shaded region in Panels B and D has a width equal to the median estimate, across participants, of the duration of sensory and motor processing pipelines. At time lags relative to response, all evidence appeared to be weighted equally in the interrogation condition (B). However, there was evidence that frames occurring at the onset of the stimulus were especially strong predictors of responses in both conditions (A). Looking at the free response condition data in Panel D, we see that evidence that was probably gathered after a decision (i.e., evidence probably in processing pipelines at the time of response) appears to have a greater effect on confidence, than the evidence that was probably processed prior to decisions. Error bars represent ±1 SEM of the mean. Plotting details in Subsection “Plotting Procedure.” SEM = standard error of the mean. See the online article for the color version of this figure.

Analyses looking at the effect of evidence on choice allowed us to further investigate the possibility that observers used implicit decision thresholds in the interrogation condition. We looked at the average evidence fluctuations in the direction of the choice made, as a measure of the strength of the relationship between evidence presented at different time lags and the response made (Figure 6A and 6B; Experimental Method section; Resulaj et al., 2009). Looking at average evidence fluctuations at time lags relative to the onset of the trial (Figure 6A), it appears that evidence presented near to the start of the trial has a stronger effect on the decision. A number of mechanisms have been proposed to account for such primacy effects, including implicit decision thresholds (Kiani et al., 2008; Tsetsos et al., 2012). However, an implicit threshold seems inconsistent with the pattern observed in average fluctuations at time lags relative to response (Figure 6B). This pattern suggests all evidence in interrogation trials, whenever it is presented, is weighted equally. When looking at time lags relative to response (Figure 6B), any stimulus onset effects are presumably averaged out due to variability in trial duration. Model-free analyses of evidence weighting time courses must be interpreted cautiously because they are affected by both the weight given to sensory information received at different time points and the mechanics of the decisionmaking process itself (Okazawa et al., 2018). Accordingly, we return to these effects when we consider the fit of the models to the data (Modeling Results section). For the free response condition, a notable feature in Figure 6B is the apparent negative effect on choice, of evidence gathered a long time prior to response. Such long trials may only occur when observers initially receive misleading evidence, explaining the effect (see also Figure 5 of Charles & Yeung, 2019).

### Tests III and IV

**Figure 7:**
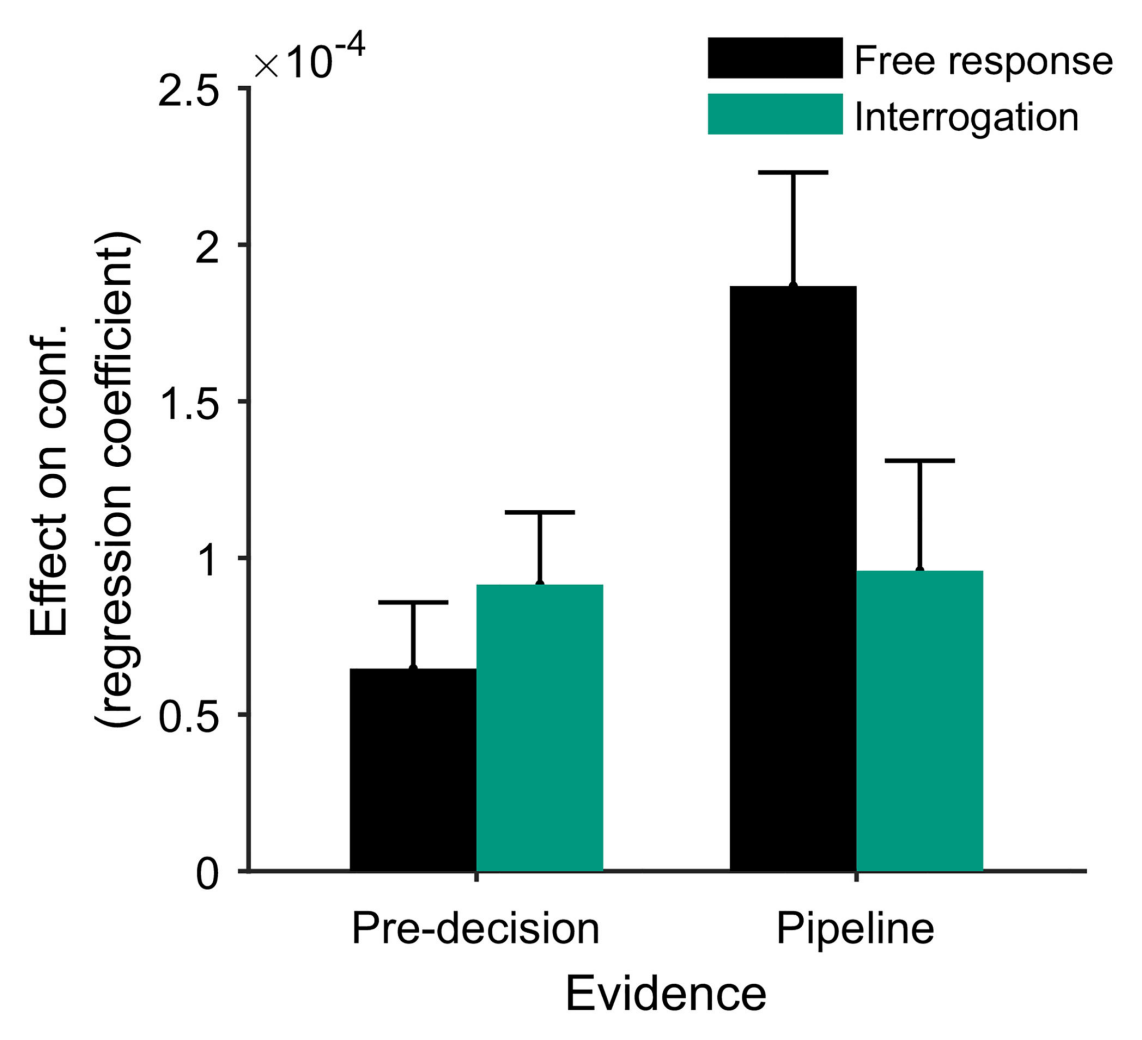
The Effect of Predecision and Pipeline Evidence on Confidence in the Two Conditions *Note*. The *y*-axis represents the values of coefficients produced by the ordinal regression onto confidence (Experimental Method section). We hypothesize that in the interrogation condition all evidence is processed prior to a decision, and therefore that there is no pipeline evidence. Nevertheless, as discussed in the main text, we artificially divide up the evidence presented, in the same manner as in the free condition, for the purpose of comparison. Unlike in other plots, error bars represent 95% confidence intervals. conf. = confidence. See the online article for the color version of this figure.

The core model and its variants make a critical prediction about the dependence of confidence on predecision versus pipeline evidence. We looked at the rank correlation between evidence fluctuations and confidence at different time lags (Figure 6C and 6D). We see a similar pattern in the effect of evidence on confidence, as in the effect of evidence on response. Again, all evidence appears to be weighted equally in the interrogation condition, when looking at time lags relative to response (Figure 6D), but a primacy effect becomes apparent when looking at the effect of evidence relative to trial onset (Figure 6C). On the basis of the core model and variants, we reasoned that in the free response condition, evidence presented prior to the time of a decision would have a reduced effect on confidence, once the effect of time had been accounted for (Models section). We can explore this possibility by returning to the coefficients produced by predicting confidence in an ordinal regression (Experimental Method section; Figure 7). The effect of time is taken into account in this regression because response time is included as a predictor. A *t* test on the coefficients showed that predecision evidence had a stronger effect in the interrogation condition than in the free response condition, *t*(46) = −2.4, *p* = .010, one-tailed, *d* = −0.35; Figure 7. This effect was even clearer when we baselined the predecision evidence coefficients relative to the pipeline evidence coefficients (separately for participant and condition), as we had preregistered, *t*(46) = −11, *p* = 3.9 × 10^−14^, one-tailed, *d* = −1.5, preregistered, Test IV. Comparing coefficients within the free response condition, we found that predecision evidence had a smaller effect than pipeline evidence, *t*(46) = −6.2, *p* = 7.3 × 10^−8^, one-tailed, *d* = −0.90, preregistered, Test III; Figure 7.

In summary, regarding the four qualitative predictions of the variants of the core model (Tests II–IV also hold for the core model itself), we find that all are confirmed in this preregistered study. As such, the results provide support for the general modeling approach taken. We therefore extended our approach to use detailed computational modeling to adjudicate among the DDM variants considered, based on their quantitative fit to these data. As part of this endeavor, we address some of the more surprising features of the empirical results, including the finding (Figure 5) that confidence varied little as a function of response time in our interrogation condition.

## Modeling Method

We next sought a stronger, quantitative test of the models: A key motivation of our study was to provide a DDM framework for confidence that could account for key relationships observed in confidence data, and the strength of these effects. In doing so, we further aimed to determine which DDM model variant provides the best account of the data.

As discussed in Introduction section and further in General Discussion section, we do not fit the models to response times and responses themselves. Instead we fit the models to participants’ confidence reports, given the stimuli they observed, the responses they made, and their response times. The focus of our investigation is confidence, and fitting to confidence reports makes it possible to use recently developed mathematical expressions that are computationally cheap enough to enable trial-by-trial modeling of dynamic stimuli (Introduction section; Calder-Travis et al., 2023). These expressions allow us to evaluate directly—that is, without the need for laborious and inherently noisy simulation—the likelihood of each empirically observed data point (i.e., each trial-wise confidence report), given assumptions about the computations underlying those data (i.e., theories about how confidence is determined) and given the stimulus presented, response observed, and associated response time on that trial. We can use these expressions to identify the parameters that allow a model variant to best fit empirically observed confidence data—that is, we identify model parameters that maximize the probability of observed confidence reports, according to the model variant’s specific account of how confidence is generated. To compare quantitative fits to the observed data across model variants, we can use a cross-validation approach. This involves assessing which model, when fit to a subset of each participant’s data, most accurately predicts their confidence reports in a different subset of the data. We thus identify, for each participant separately and in aggregate, which of the 10 model variants best predicts unseen confidence data.

The key strength of fitting confidence specifically, using computationally cheap expressions, is that we can fit the models to single-trial confidence reports, rather than aggregate features such as the mean and standard deviation of participants’ confidence reports within a condition. Conversely, the fitting we use requires the models to make tailored predictions given the key features of individual trials (i.e., the stimulus, response, and response time; Park et al., 2016). Moreover, we fit data from both the free response and interrogation condition simultaneously, and all parameters relevant to both conditions are shared between them. This provides a very strong test for the models because we are requiring the models to fit two very different sets of patterns using the same parameter values (Ratcliff, 2006). Finally, with this approach, response times and responses effectively become held out data that the models are not fit to. We can ask the models to predict these data—that is, ask whether model parameters that are chosen to optimize the fit to confidence reports nevertheless give reasonable fits to participants’ choices and response times—providing a very strong test (Kiani et al., 2014).

Following the approach described in Models section, we model confidence reports as ordinal data (see also Yu et al., 2015). For computational modeling, we divide confidence reports into four bins on a participant-by-participant basis. That is, models and parameters are evaluated according to the likelihood they ascribe to observing a confidence report in a particular quartile of each participant’s distribution of confidence reports across trials.

### Model Predictions for Confidence

In Calder-Travis et al. (2023), we have derived approximate expressions for the confidence of DDM observers under both interrogation and free response conditions, allowing for drift-rate variability, time-dependent thresholds and metacognitive noise. The approximations are computationally tractable and yet closely match detailed simulations (Calder-Travis et al., 2023). These expressions were derived according to the experimental design described above, in which participants choose between two equally probable options for which the stimulus provides two evidence signals, one for each option. The evidence signals vary over the course of a trial according to a normal distribution around a mean value, but are constant within short “frames.” Using the derivations in Calder-Travis et al. (2023), we capitalize on the dynamic nature of these stimuli—which introduces variability within and between trials (Figure 3)—to make trial-by-trial predictions for confidence.

A key reason for the low computational cost of these expressions, that makes trial-by-trial modeling of confidence in a fluctuating-stimulus task possible, is that a relatively simple probability distribution can be found for the final state of the accumulator in the free response condition. Namely, the final state of the accumulator follows a normal distribution, given a specific decision threshold crossing time and threshold crossing location. Ratcliff (1980) showed that in the absence of decision thresholds, even with within-trial time-varying drift rate, the probability distribution over the state of the evidence accumulator would remain normal. This is the situation in the free response condition following the crossing of the decision threshold: There are no longer active decision thresholds and we have a time-varying drift determined by the particular stimulus shown on each trial. Even additionally under conditions of trial-to-trial drift-rate variability, the posterior distribution over the final state of the evidence accumulator remains approximately normal (Calder-Travis et al., 2023).

The present work builds on Calder-Travis et al. (2023) in several ways. Notably, Calder-Travis et al. (2023) only considered a calibrated Bayesian readout of confidence, not a miscalibrated Bayesian readout or, as in the core model, a readout of the final state of the accumulator. We provide the necessary extension in Appendix A. Here, we model decision thresholds as symmetric and flat, or symmetric and linearly decreasing, in the case of decreasing decision thresholds, reflecting key model variants we aim to evaluate. Finally, we add a free parameter to allow for the possibility of lapses in confidence reports where confidence does not follow from the usual process and instead is randomly generated to fall within one of the four confidence bins with equal probability (Appendix A). We assume that lapses occur with equal probability regardless of response, response time, and evidence presented. In addition to this, on free response trials where the response time is faster than the estimated duration of sensory and motor processing pipelines (a free parameter in all models), we model confidence reports as certainly the result of a lapse. Following Calder-Travis et al. (2023), we assume that observers ignore the fact that evidence is constant within each 50 ms frame and that variability present in the stimulus will have a bigger effect on the evidence accumulation during trials in which the stimulus is being processed better (i.e., trials with high drift rate). To expand on this point, when information in the stimulus is being processed better, both the mean stimulus signal and the frame-by-frame stimulus variability exert a greater effect on the evidence accumulation, increasing both the rate of evidence accumulation, and variability in the evidence accumulation process. We assume that participants ignore this subtle effect on variability in the evidence accumulation. Calder-Travis et al. (2023) tested the effects of both assumptions and found that objective accuracy and subjective confidence remain closely related. Further details on the implementation of the model predictions are provided in Appendix A.

### Model Parameters

The models described in the Models section have between eight and 11 free parameters (Table 4). All models had a parameter for the standard deviation of accumulator noise, σ_acc_. Accumulator noise corrupts the evidence samples that drive changes in the accumulator, and it affects each evidence measurement independently. All models also had a parameter for the standard deviation of metacognitive noise, σ_*m*_ (Models section). We fit the height of the decision threshold, *a*, (with one decision threshold at *a* and the other at −*a*) and the duration of sensory and motor processing pipelines, *I*. Finally, all models had a lapse rate parameter, λ, and three parameters describing the bounds between the four confidence bins, *d*_*i*_. In the model, confidence reports are determined by the location of a continuous variable, *x*_*c*_ or −*x*_*c*_, with the sign determined by the response given such that a greater number indicates more support for the choice made. If *x*_*c*_ (appropriately signed) falls between the boundaries *d*_*i*_ and *d*_*i*+1_, then a confidence report falling in bin *i* is given (Yu et al., 2015). Further details of the roles of the parameters in the computational model are provided in Appendix A.

In addition to these shared parameters, specific model variants had additional parameters. All models that included drift-rate variability had a parameter describing its standard deviation, σ_φ_. All models with decreasing decision thresholds included a parameter describing the slope of the decision threshold as a function of time, *b* (upper and lower thresholds were then given by *a* − *bt* and −*a* + *bt* where *t* is the time spent accumulating evidence). Models using a miscalibrated Bayesian readout of confidence included one additional parameter, Γ, accounting for the incorrect beliefs of the observer. Specifically, Γ is a transformed version of the variable γ (discussed in Models section), that reflects the observer’s belief about the balance between drift-rate variability and a weighted sum of other sources of variability (Appendix A). The two other sources of variability are accumulator noise and the variability in evidence presented in different frames of the stimulus. Incorrect estimation of any of these sources of variability can lead to a value for Γ that differs from the value used by a calibrated Bayesian. For details of the exact role of the parameters see Appendix A.

### Fitting

For any given set of parameters, **ξ**, we can compute the probability of an observed confidence report for a given participant on a given trial. Assuming the confidence report given on a trial is conditionally independent of the confidence report given on any other trial, the likelihood of the parameters is then given by,

(2) $$L(\xi )=p\left(C^{\left(1\right)},C^{\left(2\right)},\ldots |\xi ,E^{\left(1\right)},R^{\left(1\right)},t_{r}^{\left(1\right)},E^{\left(2\right)},R^{\left(2\right)},t_{r}^{\left(2\right)},\ldots \right),$$

(3) $$=\underset{i}{\prod \text{​}}p\left(C^{\left(i\right)}|\xi ,E^{\left(i\right)},R^{\left(i\right)},t_{r}^{\left(i\right)}\right),$$

where *C*^(*i*)^, **E**^(*i*)^, *R*^(*i*)^, and $t_{r}^{\left(i\right)}$ are the confidence report, stimulus, response, and response time on trial *i*. When fitting to participants’ confidence reports, we use the stimuli that were actually shown to participants, along with the obtained responses and response times. We fit the models to the data by maximizing the log likelihood. All data were used in either the fitting or fit evaluation; all participants and every trial in which a confidence report was obtained were included. The confidence lapse rate parameter was included to account for any random responses, so exclusions are not necessary.

A single fit began with the evaluation of the likelihood function at 200 randomly drawn sets of parameter values. The parameter set with the highest log likelihood was used as the start point for MATLAB’s fmincon optimizer (Matlab Optimization Toolbox, 2017). For details of the limits applied to parameters during optimization, and the way the 200 candidate sets of parameters were drawn, see Appendix F.

We repeated this entire process 40 times for every fit (i.e., for each participant, model, and cross-validation fold; see below). Rerunning fitting many times improves the chance of finding the true maximum likelihood, rather than getting stuck in local maxima, and allows one to perform heuristic assessments of the optimizer’s performance (Acerbi et al., 2018, Supplemental Methods, Section 4.2).

### Model Comparison

We compared the fit of the models using five-fold cross-validation, which automatically penalizes flexibility (Lewandowsky & Farrell, 2011) and does not require explicit estimation of a penalty as in the Akaike information criterion (AIC) and the Bayesian information criterion (BIC). Every fold, 4/5 of the trials were used for training and 1/5 used as test trials. We fit the models to the training trials, searching for the values of model parameters that maximize the likelihood, that is, the values that maximize the probability of a participant’s trial-wise confidence reports given the models’ underlying computation of confidence. We then evaluated the models using the average negative log likelihood in test trials (Honig et al., 2020)—that is, we assess the degree to which each model (mis-)predicted the empirically observed confidence reports on those trials. We computed this value for each fold and averaged across folds to provide a measure of the performance of each model for each participant. We refer to this measure as the negative cross-validated log likelihood (−LLcv). A smaller −LLcv value indicates that the model was better at predicting the test data.

To determine the overall best fitting model, we computed the mean −LLcv across participants. To assess the reliability of the difference in fit between this and other models, we computed participant-by-participant, the difference between the −LLcv of all models and that of the best fitting model. We then computed the mean −LLcv difference, along with 95% confidence intervals found by bootstrapping 10,000 times.

Supplementing the model comparison in which we looked at cross-validated log likelihoods averaged across participants—which makes most sense under the assumption that all participants are described by the same model (fixed effects)—we also performed an analysis that assumes different participants may be described by different models (random effects; Stephan et al., 2009). In this further analysis, we assume that, for each model (i.e., each DDM variant), there is a specific proportion of the population that are described by this model. This assumption can be formalized in a generative model (which is separate from the DDM variants themselves and describes the frequency of the DDM variants in the population). We can invert this higher level generative model to infer the frequency of each of the lower-level DDM models in the population (Daunizeau et al., 2014; Stephan et al., 2009).

For the implementation of the higher level generative model and its inversion we used the Variational Bayesian Analysis toolbox, which utilizes a variational Bayesian inference scheme (Daunizeau et al., 2014). For such an analysis, we require estimates of the model evidence for each participant and model, that is, the probability of the data for that participant given the model (Stephan et al., 2009). Depending on the precise definition of the BIC, the BIC can either be viewed directly as an estimate of model evidence or can be viewed as an estimate after multiplication by a factor of −1/2 (as was the case here; Bishop, 2006; Lewandowsky & Farrell, 2011; Penny et al., 2004). We used BIC values calculated from a separate set of fits that were identical to the procedure described above, except that fitting was performed using all the data from a participant (with no held out “test” data). In addition to reporting estimated model frequencies in the population, we also report exceedance probabilities, which give the probability that a model describes a greater proportion of the population than any other model considered (Stephan et al., 2009).

Because these random effects model comparison analyses aim to infer model frequencies working from a prior that all models are equally likely, for this analysis, we included the two Bayesian models that we ignore elsewhere (Models section). These models are named “C” and “DC” under our model naming scheme; Table 5. As discussed, these models are ignored elsewhere because they make exactly the same predictions as two of the models in which confidence is a non-Bayesian readout of the evidence accumulated.

Using this random effects approach, we also performed various family-wise comparisons (Penny et al., 2010), again as implemented by the Variational Bayesian Analysis toolbox, to compare groups of models (e.g., non-Bayesian confidence models vs. calibrated Bayesian confidence models vs. miscalibrated Bayesian confidence models). In addition to estimates for individual model frequencies, these analyses give us estimates of the proportion of the population best described by each group of models.

To evaluate the likely performance of the model-fitting procedures, we ran a model recovery analysis, simulating data from each of the 10 models, fitting each set of simulated data with all 10 models, and then evaluating the AIC and BIC to confirm the correct model was recovered. This analysis suggested that the AIC and BIC could be too conservative in the current context, but otherwise the results were as expected (Appendix E). Importantly, apart from one tied best fit (as evaluated by the AIC that included the true data generating model in the tie), the analysis never incorrectly inferred the presence of a feature (V/D/C/M) that was not in fact present in the underlying generative model.

To plot the behavior of the fitted models, for comparison with detailed features of our empirical data, we ran a simulation for each participant, using the fitted parameters. We use the parameters resulting from the fits to the entire data set (with no training-test split applied; further details in Appendix B).

## Modeling Results

Using computational modeling, we aimed to determine which of the 10 DDM models of confidence fit the data from the experiment best (Table 1; Experimental Method section) and whether any DDM model could provide an adequate quantitative account of confidence. As set out in the Modeling Method section, we fit specifically to confidence reports—the focus of our investigation—not to responses and response times. We will return later to consideration of responses and response times.

### Model Comparison

**Figure 8:**
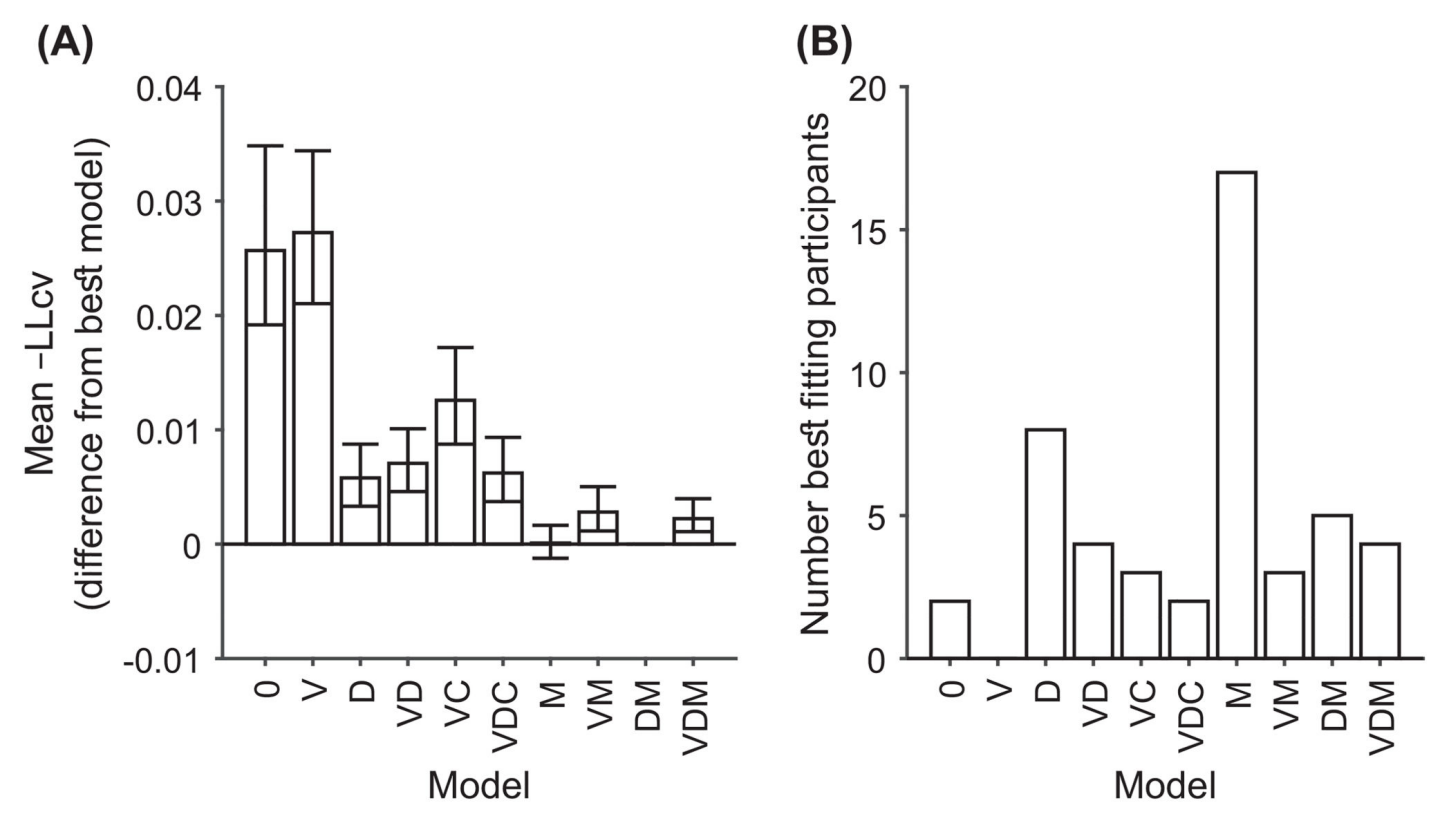
Model Comparison Results *Note*. (A) Negative cross-validated log likelihood (−LLcv) relative to the model with the lowest mean −LLcv (Model DM with a mean −LLcv of 1.338) and (B) number of participants for which each model provided the best fit. A lower value of –LLcv in Panel A indicates better fit. Models in which confidence reflects a miscalibrated Bayesian readout fit best (Models M, VM, DM, VDM). Unlike in other plots, error bars represent 95% bootstrapped confidence intervals. V = drift-rate variability; D = decreasing thresholds; C = calibrated; M = miscalibrated.

We fit the models and evaluated their performance using cross-validation. Model M, in which the observer uses a miscalibrated Bayesian readout of confidence, and Model DM, which also includes decreasing decision thresholds, provided the best fit to the data on average (Table 1 and Figure 8A). The fits for Models VM and VDM were nearly as good. These models, in addition to having the features in M and DM, respectively, include drift-rate variability. All these four models share a miscalibrated Bayesian readout for confidence. Models in which confidence reflects the final state of the accumulator, or in which confidence reflects a calibrated Bayesian readout, performed worse (Models 0–VDC in Table 1). These results suggest that confidence reflects a miscalibrated Bayesian readout, but it is not clear whether observers used time-dependent thresholds or whether drift-rate variability is present.

Considering fits to individual participants (Figure 8B), Model M was the best fitting model for the greatest number of participants. We conducted a random effects model comparison analysis to explore the distribution of models over participants in a more principled manner. This analysis directly estimates the proportion of the underlying population that are best described by each model (Modeling Method section). Model M again performed the best (estimated model frequency: 0.766; exceedance probability: 1.000). Model D (Table 1) was the next best model according to this analysis, although its performance was clearly worse (estimated model frequency: 0.083; exceedance probability: 0.000). To be precise, Models D and DC were joint second best (Table 5): Both models make exactly the same predictions and have the same associated statistics.

Using the random effects analysis, we could also compare families of models (Penny et al., 2010). This analysis reinforced our conclusion that, in general, participants use a miscalibrated Bayesian readout for confidence (estimated family frequency: 0.824; exceedance probability: 1.000). Regarding drift-rate variability and time-dependent thresholds, this analysis provided more definitive answers than when comparing individual models. An absence of drift-rate variability was estimated to be most common (estimated family frequency: 0.990; exceedance probability: 1.000), as were flat decision thresholds (estimated family frequency: 0.775; exceedance probability: 1.000). Nevertheless, it is worth noting that because this random effects analysis was based on BIC values, it will inherit the tendency of the BIC to favor simpler models (Appendix E; Lewandowsky & Farrell, 2011).

### Fits of the Best Model

**Figure 9:**
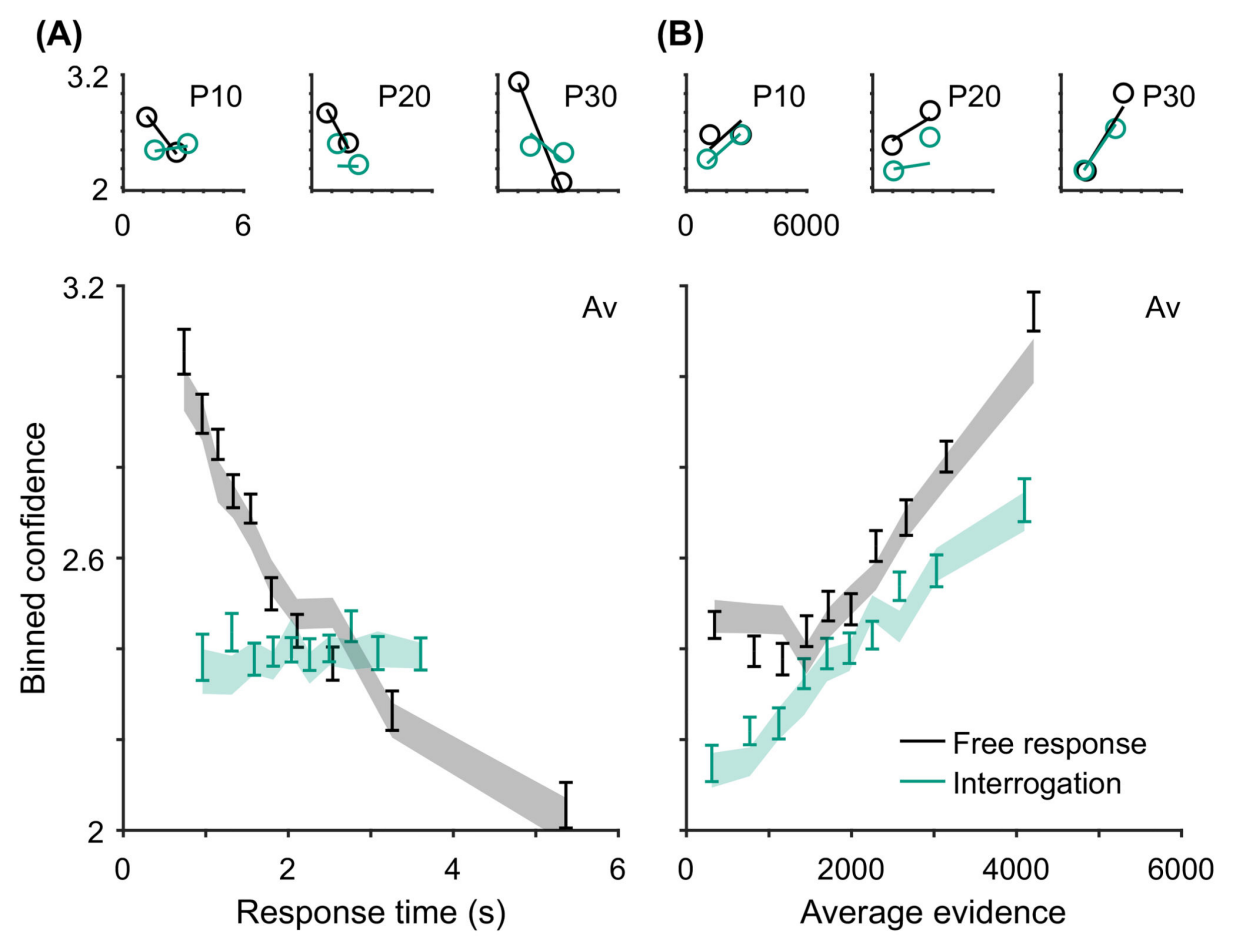
(A-Av) Effect of Response Time and (B-Av) Average Evidence on Confidence in the Data (Error Bars) and in the Best Fitting Model, Model M (Shading) *Note*. Model M accounted well for quantitative patterns in the effects of both response time and average evidence, and in differences between the two conditions. In Panel B average evidence is computed by summing, over all frames, the difference in dots presented in the two arrays, before taking the absolute value and dividing by the time the stimulus was presented for. In both A-Av and B-Av error bars and shading represent ±1 SEM. A-P10, A-P20, A-P30, B-P10, B-P20, B-P30 show corresponding data (circles) and model fits (lines) for three individual participants. Plotting details in Subsection “Plotting procedure.” Parameter values for key fitted models are given in Appendix F. Av = average; SEM = standard error of the mean; M = miscalibrated. See the online article for the color version of this figure.

Having seen that Model M performed the best over a range of measures, we next asked whether this model could account for detailed patterns in our empirical confidence data. As shown in Figure 9A-Av, Model M is capable of simultaneously capturing the effects of response time in the two conditions, with confidence decreasing as a function of response time in the free response condition while remaining relatively constant in the interrogation condition. Not only does Model M capture these effect qualitatively but also quantitatively, with a very high degree of overlap between the error bars and error shading representing ±1 SEM of the data and model fits. The performance of the model can be explained by considering the main feature of the miscalibrated Bayesian observer model: It includes a parameter to capture the observer’s estimate of the relative magnitude of different sources of variability, which is no longer assumed to match the true ratio. As discussed, this estimate determines the strength of the time penalty for confidence that the observer applies (Models section). Participants appear to apply a bigger time penalty for confidence than a calibrated observer should. This conclusion follows most clearly from Model M, in which there is no trial-to-trial variability in drift rate. In this case, for a calibrated observer who correctly believes there is no trial-to-trial variability in drift rate, the Bayesian confidence readout does not use time, only the final state of the accumulator, and there should be no time penalty for confidence at all (given the stimuli we studied; Introduction section; Models section; Moreno-Bote, 2010). Only if the observer erroneously believes trial-to-trial variability in drift rate is present, will the Bayesian readout of confidence take into account both the final state of the accumulator and the time spent accumulating evidence. In this case the time penalty for confidence will apply, and by definition be too strong. A time penalty for confidence that is too strong explains the surprisingly slow change in confidence with response time in the interrogation condition that we observed empirically, despite the fact that in this condition the evidence accumulation is not bounded by a decision threshold and will accumulate rapidly: The effect of the time penalty cancels out the effect of accumulating more evidence.

Model M likewise accounts qualitatively and quantitatively for the effect of (unsigned) average evidence on confidence (apart from a slight quantitative deviation of the data and model at the very highest values of average evidence in the free response condition, where ±1 SEM windows for data and model no longer overlap; Figure 9B-Av). The model captures how this relationship differs in the free response and interrogation conditions: Confidence is higher in the free response condition for a fixed level of average evidence, and this effect is greatest at high and low average evidence. High and low average evidence is associated with shorter stimulus presentation; as stimulus duration increases, variability in average evidence decreases. One possible explanation for the difference between free response and interrogation trials at high and low average evidence is that, in the free response condition, short trials occur when noise in the evidence accumulation happens to quickly drive the accumulator to the decision threshold. In contrast, in the interrogation condition, on average little evidence is accumulated in a short trial. The overall quantitative pattern will be a complex result of the various evidence accumulation mechanisms, and how they are affected by the presence or absence of decision thresholds. Therefore the success of the model supports the idea that the mechanisms and assumptions built into the model match the real evidence accumulation and confidence mechanism.

Regarding the associated plots of individual participant data (Figure 9-P10, A-P20, A-P30, B-P10, B-P20, B-P30), we see that fits are reasonable but fairly noisy. This is a situation that we will see repeated in other plots showing fits of the best model to individual participants and can be anticipated from the shape of the data set collected: We collected modest amounts of data from a large number of participants (48 participants; 640 trials per participant; Experimental Method section).

**Figure 10:**
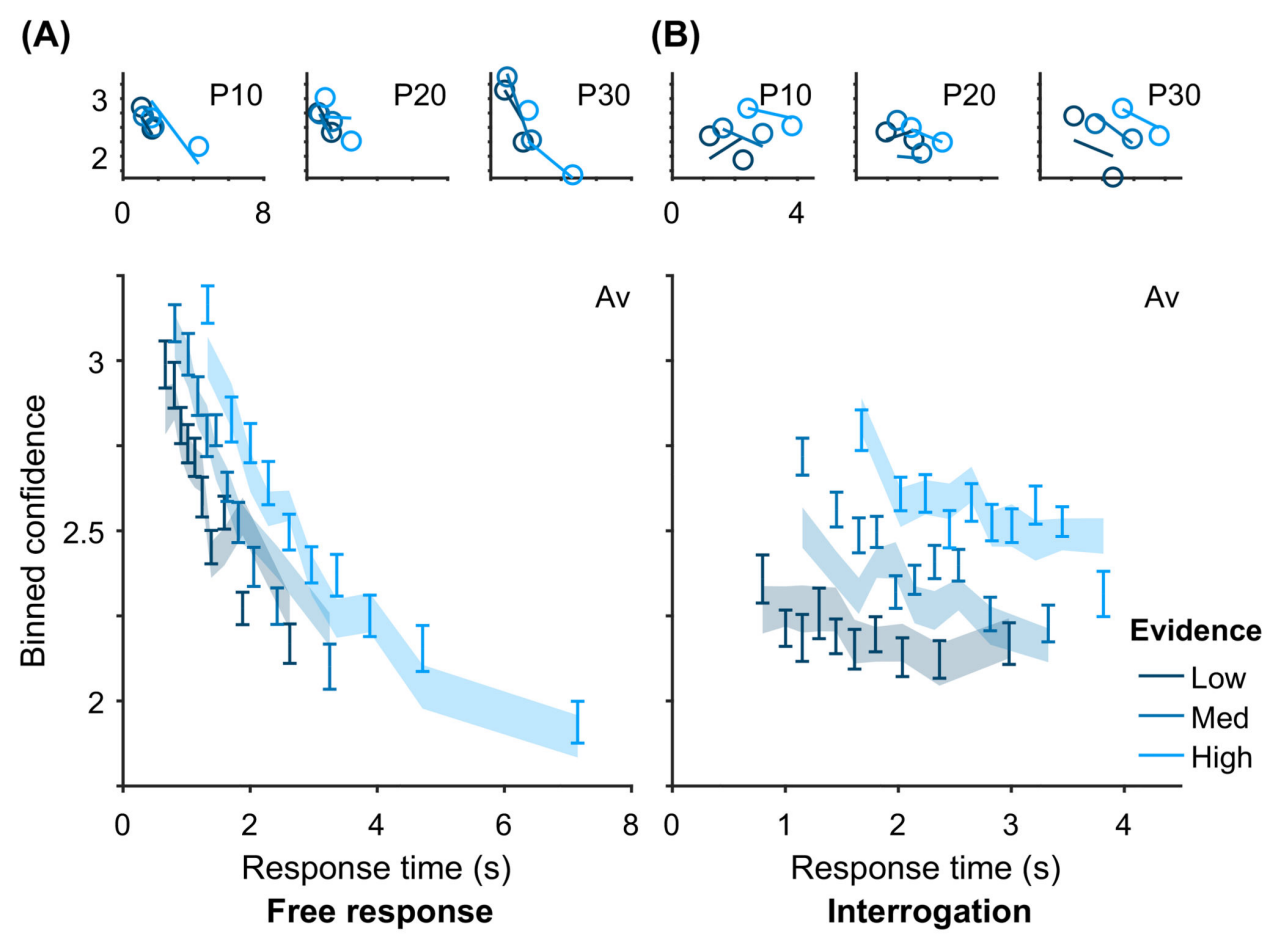
Effect of Response Time and Evidence, Considered Simultaneously, in the (A-Av) Free Response and (B-Av) Interrogation Conditions *Note*. Effect on confidence shown for the data (error bars), and in the best fitting model, Model M (shading). Except at the longest and shortest response times, Model M accounted well for the simultaneous effect of time and evidence in both conditions (A and B). Evidence is computed by summing, over all frames, the difference in dots presented in the two arrays, before taking the absolute value. We separated trials into tercile bins according to this value, separately for the two conditions and each participant. In both A-Av and B-Av, error bars and shading represent ±1 SEM. A-P10, A-P20, A-P30, B-P10, B-P20, B-P30 show corresponding data (circles) and model fits (lines) for three individual participants. Plotting details in Subsection “Plotting Procedure.” Av = average; SEM = standard error of the mean; M = miscalibrated. See the online article for the color version of this figure.

The ability of Model M to account for the separate effects of both response time and evidence strength does not depend on the correlation between these factors: The model predicts the independent effects of both. In Figure 10, we plot for the free response (Figure 10A-Av) and interrogation conditions (Figure 10B-Av), confidence as a function of response time, separately for low, medium and high total evidence trials. Model M captures qualitatively the effect of response time at all levels of evidence and also accounts for increased confidence with greater evidence at a fixed response time. The model also captures qualitative differences between the two conditions, such as the stronger effect of evidence at fixed response time in the interrogation condition. Two mechanisms allow the model to account for the independent effects of response time and evidence in the free response condition. First, the time penalty for confidence leads to an effect of time independent of the effect of evidence. Second, the accumulation of pipeline evidence allows for an effect of evidence that is independent of any effect of response time. Although in general the model provides a close quantitative fit to the empirical data, there appear to be some systematic quantitative deviations for the fastest and slowest response times within an evidence bin, as indicated by the lack of overlap in a limited number of cases between ±1 SEM for the data and model fits. For example, in the interrogation condition for medium evidence and fast response times, the model appears to slightly underestimate confidence (Figure 10B-Av). This effect is difficult to explain because at both low and high evidence the model accurately fits the level of confidence at fast response times. Possibly the small deviation at medium evidence is the result of random fluctuations in the data.

**Figure 11:**
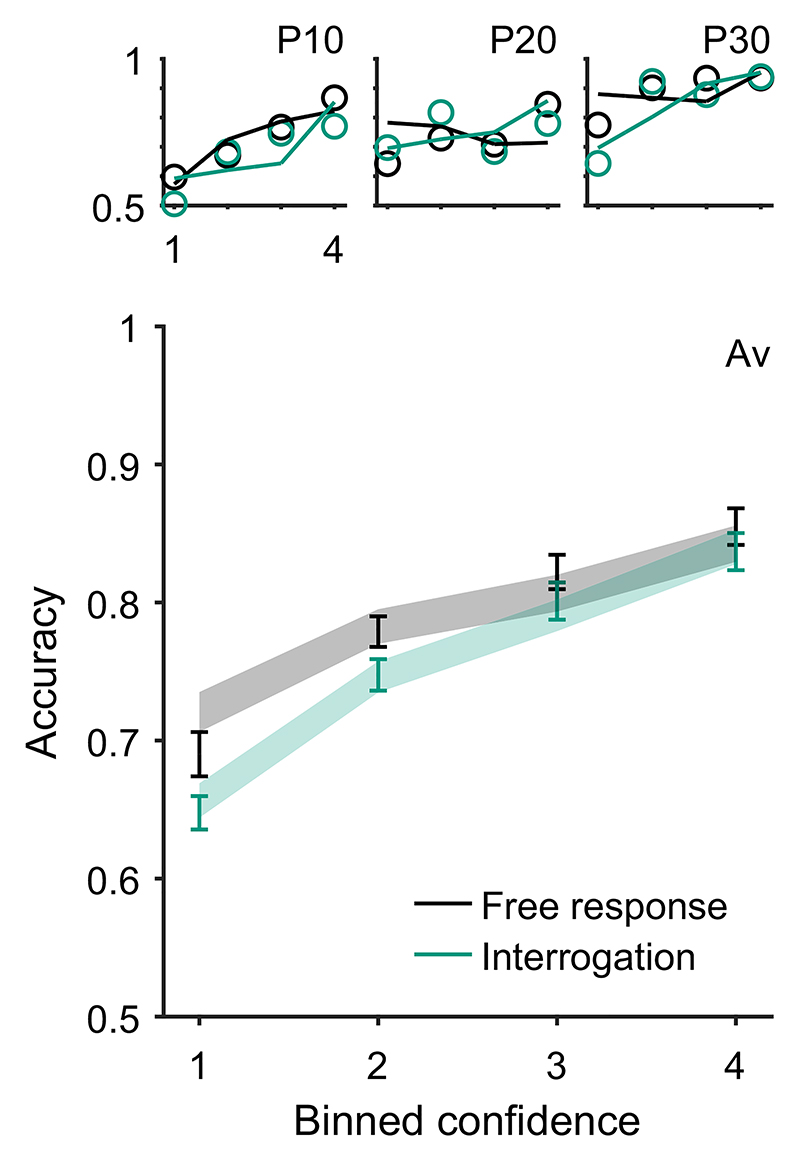
(Av) The Relationship Between Confidence and Accuracy in the Data (Error Bars) and in the Best Fitting Model, Model M (Shading) *Note*. Generally, the model captured quantitative and qualitative patterns well. In Panel “Av” error bars and shading represent ±1 SEM. Panels “P10,” “P20,” and “P30” show corresponding data (circles) and model fits (lines) for three individual participants. Plotting details in subsection “Plotting Procedure.” Av = average; SEM = standard error of the mean; M = miscalibrated. See the online article for the color version of this figure.

In addition to the effects of response time and evidence, another key pattern for any model of confidence is the relationship between accuracy and confidence. The model provided an excellent fit to the accuracy at different confidence levels in both conditions both qualitatively and quantitatively (Figure 11-Av). The qualitative and quantitative model predictions result from the combined effect of all hypothesized evidence accumulation and confidence readout processes. Hence, the close fits to the data support the claim that the miscalibrated Bayesian readout describes well the way humans relate the evidence they have received to a subjective sense of confidence. There was one exception to the excellent quantitative fits: The model overestimated accuracy at the lowest level of confidence in the free response condition (the model predicted 72% accuracy but the true value was 69%). A possible explanation for this result is that participants make response lapses in the free response condition, of which they are aware, and correspondingly indicate on the confidence reporting scale. The models include lapses in confidence reports, but not response lapses, and hence could not capture such an effect. Nevertheless, even here the model correctly fitted the qualitative pattern of greater accuracy in the free response condition, than the interrogation condition, for this lowest level of confidence.

**Figure 12:**
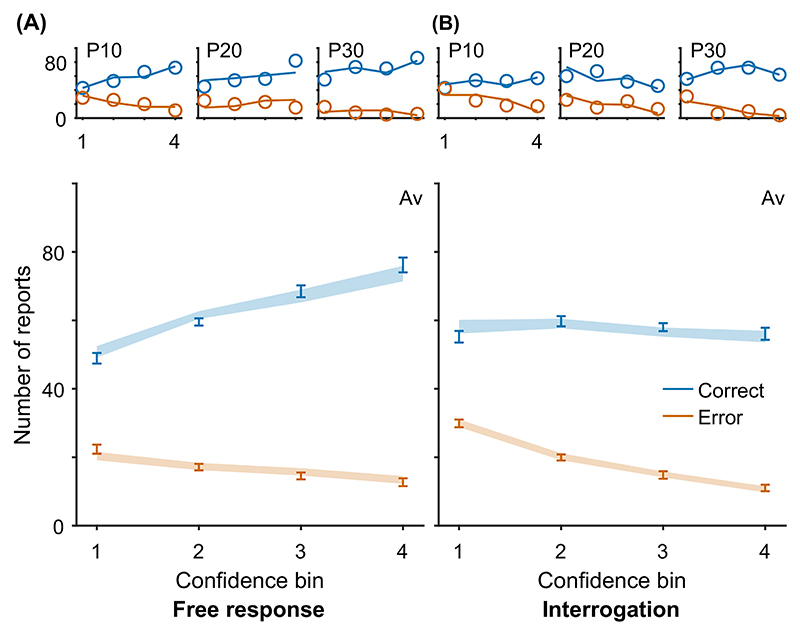
Fit of the Best Model (Model M) to the Number of Confidence Reports in Each Confidence Bin in (A-Av) the Free Response Condition and in (B-Av) the Interrogation Condition *Note*. The model fit is shown in shading and the data with error bars. The model captured quantitative and qualitative patterns very well. In both A-Av and B-Av error bars and shading represent ±1 SEM. A-P10, A-P20, A-P30, B-P10, B-P20, B-P30 show corresponding data (circles) and model fits (lines) for three individual participants. Plotting details in subsection “Plotting Procedure.” Av = average; SEM = standard error of the mean; M = miscalibrated. See the online article for the color version of this figure.

Complementing these findings regarding the success of the model at fitting accuracy as a function of confidence, the model additionally does an excellent job of accounting qualitatively and quantitatively for the number of reports in each confidence bin, separately for free response (Figure 12A-Av) and interrogation trials (Figure 12B-Av). Furthermore, this is the case following both correct and error responses. The model captures both the qualitative and quantitative patterns: ±1 SEM windows for the data and model are highly overlapping. Fits to individual participants are a little noisier, but in general the model does well at capturing the qualitative patterns and how these differ across participants, and across the two conditions for individual participants (Figure 12). It is difficult to attribute this success of the model to one mechanism: All components of the model are centrally involved in generating these distributions, and hence supported by these results.

**Figure 13:**
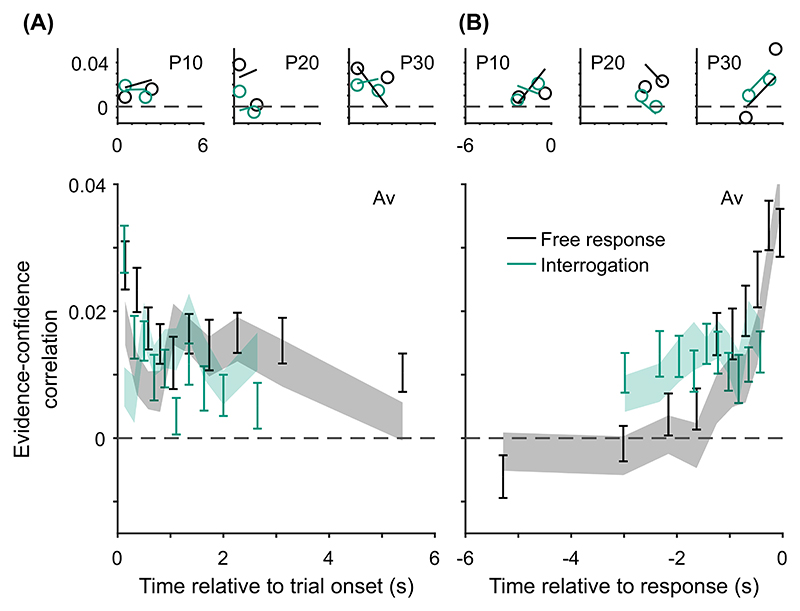
(A-Av and B-Av) The Effect of Evidence Fluctuations on Confidence in the Data (Error Bars) and in the Best Fitting Model, Model M (Shading) *Note*. Effects shown (A) at times relative to trial onset and (B) at times relative to the response. To measure this effect, we computed the rank correlation (Kendall’s τ) between evidence fluctuations and confidence. The model accounted well for the effect of evidence at time lags relative to response (B-Av), in both conditions. However, the model failed to capture the strength of the effect of evidence presented at the onset of the stimulus (A-Av). In both A-Av and B-Av, error bars and shading represent ±1 SEM. A-P10, A-P20, A-P30, B-P10, B-P20, B-P30 show corresponding data (circles) and model fits (lines) for three individual participants. Plotting details in subsection “Plotting Procedure.” Av = average; SEM = standard error of the mean; M = miscalibrated. See the online article for the color version of this figure.

Figure 13 plots the degree to which trial-wise confidence ratings are predicted by evidence at different time points relative to trial onset (Figure 13A) and response (Figure 13B). Quality of the model fits differ depending on whether we look at data plotted relative to trial onset or relative to response time. It is when plotting data relative to trial onset that the model does not perform as well as elsewhere: While the model correctly predicts evidence–confidence correlations that on average are close to the true values, the model fails to capture qualitatively the effect of evidence presented at the beginning of trials (Figure 13A-Av). Specifically, both in the free response and interrogation conditions, Model M underestimates the effect of very early evidence. The model assumes all evidence presented over the course of a trial is given equal weight as it is accumulated. Therefore, the deviation of the model from the data provides further support for the idea that there may be an overweighting of evidence presented early in a trial (Experimental Results section).

In contrast, when looking at data relative to response time (Figure 13-Av), the model captures the qualitative patterns in both conditions (relatively constant evidence–confidence correlation in the interrogation condition and a sharply increasing correlation in the free response condition). Furthermore, the model accurately captures these patterns in a quantitative sense too, with a large degree of overlap between ±1 SEM windows for the data and model in both conditions. In particular, the model accurately captures the critical pattern in the free response condition of a weak correlation between confidence and evidence presented well before a response. It also accounts quantitatively for the strong effect of evidence presented immediately prior to a response, which will be in sensory and motor processing pipelines at this time (Introduction section; Resulaj et al., 2009). Unlike predecision evidence, which only affects confidence by changing the time at which the decision threshold is crossed, in the model pipeline evidence can directly affect the final balance of evidence (Models section; Pleskac & Busemeyer, 2010), explaining its far stronger effect on confidence. Although the pattern in the free response condition is largely quantitatively captured, there are some small deviations of the model from the data: For evidence presented around 1.5 s prior to response, the model appears to underestimate the effect of this evidence on confidence. One possibility is that these differences arise because there is significant between trial variation in the duration of the processing pipeline, a commonly made assumption (e.g., Ratcliff & McKoon, 2008; van den Berg, Anandalingam, et al., 2016).

Still considering data relative to response, the model again quantitatively captures the effects observed in the interrogation condition, with evidence at all time points contributing approximately equally to confidence, and the ±1 SEM windows of the model showing good overlap with the data (Figure 13B-Av). On very close inspection, the model slightly overestimates the effect of evidence received close to a response. In the model, evidence from all time points is weighted equally in the observer’s decision. However, on short trials, the effect of individual frames on confidence is likely to be greater because fewer frames are used to determine confidence. Note that in Figure 13B-Av, short trials will only contribute to plotted time points close to the response. Finding that the model accounts well for the effect of evidence in the run up to a decision supports the model’s assumptions, including the assumption that participants continue accumulating evidence until the end of each trial and that participants do not use an implicit decision threshold (Experimental Results section). Considered from the other direction, these aspects of the model offer us with an explanation for the patterns observed in the data.

### Alternative Models

**Figure 14:**
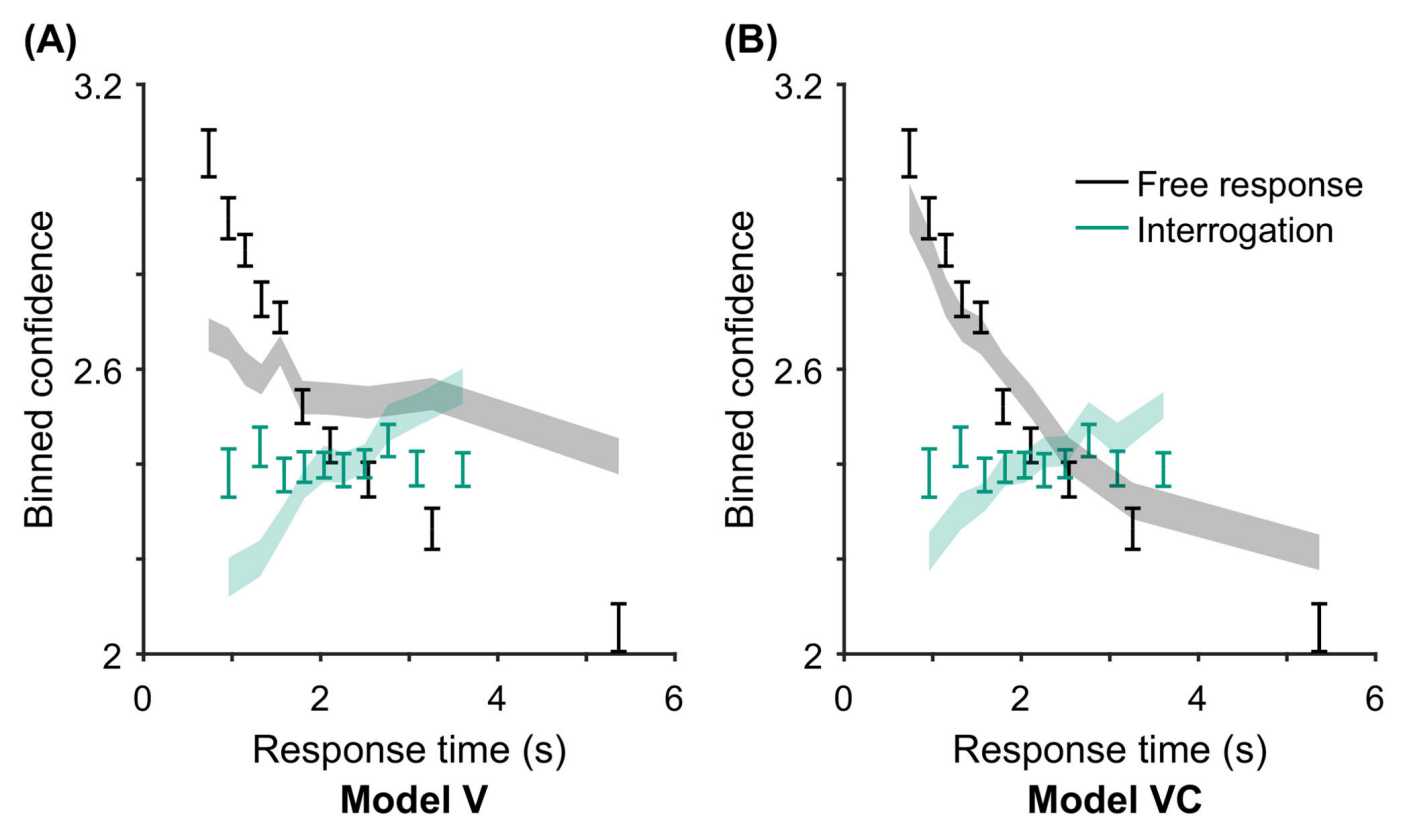
Model Fits for Two of the Losing Models *Note*. Specifically, the effect of response time on confidence in the data (error bars), and in Models V and VC (shading). (A) Model V did not capture the strength of the effect of response time on confidence in the free response condition, (B) while Model VC slightly underestimated this effect. Error bars and shading represent ±1 SEM. Plotting details in subsection “Plotting Procedure.” Parameter values for key fitted models are given in Appendix F. SEM = standard error of the mean; V = drift-rate variability; C = calibrated. See the online article for the color version of this figure.

We next sought to understand why Model M fit better than others. 2DSD theory struggles to account for the strength of the relationship between confidence and time (Introduction section; Pleskac & Busemeyer, 2010), and Model V is very similar to 2DSD. While Model V can account for some decrease in confidence with time in the free response condition, because it contains drift-rate variability (Introduction section; Pleskac and Busemeyer, 2010), it clearly struggles to capture the strength of this relationship (Figure 14A), with data and model many standard errors apart for short and long trials. This is very similar to the conclusion drawn by Pleskac and Busemeyer (2010) in relation to 2DSD. In the interrogation condition, Model V does not even match the qualitative pattern: Model V predicts increasing confidence with response time but this pattern is not apparent in the data.

It may be more surprising that models using a calibrated Bayesian readout for confidence (Models VC and VDC) also struggle to account for the relationship between confidence and time. A Bayesian readout model can predict decreasing confidence with response time due to the time penalty for confidence (Introduction section), so we might expect these models to perform well. Model VC, which uses a calibrated Bayesian readout, only slightly underestimates the decrease in confidence with response time in the free response condition, but it struggles to account for the finding that confidence changes little with response time in the interrogation condition (Figure 14B). Specifically, ±1 SEM windows for the data and model fits do not overlap for short and long trials. The mismatch between the observer’s model of the environment and the true generative model that is present in Model M, but not in Model VC, affects the strength of the time penalty for confidence. This mismatch appears to be crucial in accounting for the pattern in the interrogation condition, with the time penalty for confidence in Model VC not being strong enough.

### Simulating Responses and Response Times

We have fit the models to confidence reports given the response, response time, and evidence presented on each trial, finding that models with a miscalibrated Bayesian readout of confidence can provide a very good to excellent fit to most aspects of the confidence data. While we have not fit responses and response times, a core hypothesis motivating all models used is the idea that the same process that generates decisions also generates confidence (Figure 2A). Given that we now have estimates for all the parameters of the evidence accumulation process that leads to confidence (Table 4), if our hypothesis is correct, we should have estimates of all the parameters required to simulate responses and response times (see Kiani et al., 2014 for related approach). It may be that some parameters have a big effect on accuracy, but a small effect on confidence, and so are relatively poorly estimated from fits to confidence data alone. A good example of such a parameter is decision threshold height. It appears to be weakly constrained by confidence data as, in the parameter fits for Model M, there is a large spread in the fitted values across participants (Appendix F). On the other hand, bound height likely has a very strong effect on response times and accuracy and hence would be well constrained if we had directly fit to these quantities instead of aiming to predict them. Nevertheless, we expect simulations using parameters from confidence fits to at least approximate properties of responses and response times.

**Figure 15:**
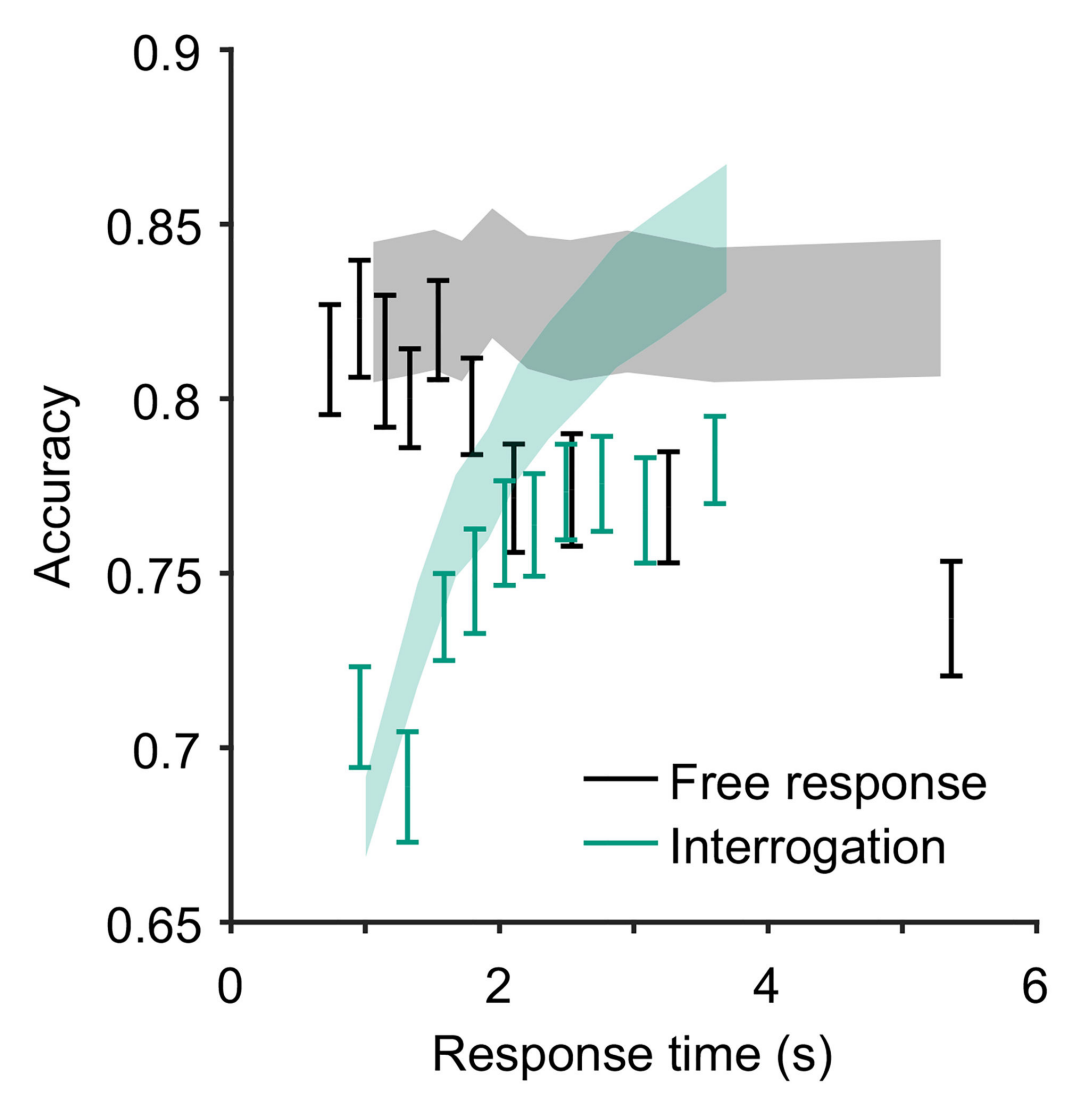
The Effect of Response Time on Accuracy in the Data (Error Bars) and in Simulations Using the Best Fitting Model for Confidence, Model M (Shading) *Note*. The simulated behavior of the model was sensible, although there were clear differences to the data. The accuracy of model-simulated responses was too high at long response times. Error bars and shading represent ±1 SEM of the mean. Plotting details in subsection “Plotting Procedure.” SEM = standard error of the mean; M = miscalibrated. See the online article for the color version of this figure.

Without any additional fitting, we simulated new stimuli and trials using the model that best fit the confidence data (Model M) and the parameters from the fits to confidence. We simulated entire diffusion processes, from onset, through decision, to confidence report (Appendix B). The accuracy of simulated responses, and how accuracy changed with response time was sensible—in particular the range of simulated accuracy values largely overlapped with the range of accuracy values in the data—although there were clear differences between the model simulations and the data (Figure 15). In the interrogation condition model simulations qualitatively matched the data, with an increase in accuracy with response time. Furthermore, there was good match between the simulations and the data for shorter trials in this condition in terms of overlapping ±1 SEM windows for data and model simulations. For longer trials the model simulations exhibited a higher level of accuracy than the data. In the free response condition, model simulations showed no change in accuracy with response time, which differs qualitatively from the decrease in accuracy at longer response times observed in the data. Similar to the interrogation condition results, model fits closely matched the data quantitatively for short trials, but produced higher levels of accuracy than observed in the data for longer trials.

We did not include in our models the possibility of lapse responses (i.e., evidence-independent guesses), which would reduce accuracy (there was only the possibility for lapse confidence reports). However, the observation that simulated accuracy is greater than the accuracy in the data specifically at late response times (Figure 15) suggests a subtler explanation. In particular, given the ubiquity of variations in attention and motivation in human performance (Macdonald et al., 2011; Robertson et al., 1997; Smallwood & Schooler, 2006), it may be that drift-rate variability—which causes slow errors (Ratcliff & McKoon, 2008) and limits accuracy for slow interrogation task responses (Ratcliff, 1978)—is important in accounting for decisions and response times, even if it has relatively little effect on confidence. This line of reasoning suggests that if we fit to responses, response times and confidence simultaneously, we may find more evidence for models featuring drift-rate variability.

**Figure 16:**
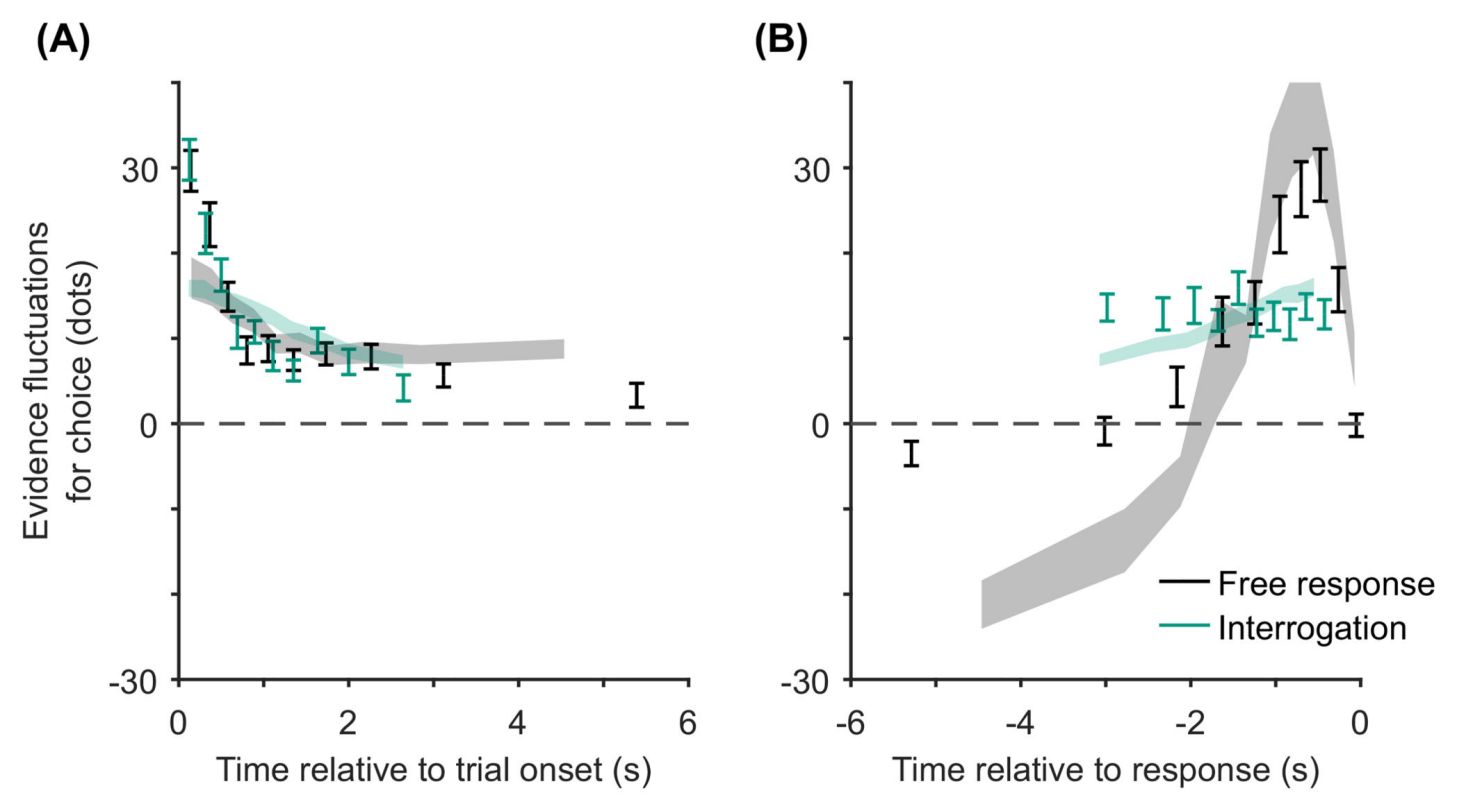
The Effect of Evidence Fluctuations on Choices, in the Data (Error Bars), and in Simulations From the Best Fitting Model for Confidence, Model M (Shading) *Note*. Effects shown (A) at times relative to trial onset and (B) at times relative to the response. The model simulations were generally reasonable, and captured some key qualitative effects. The model simulations did not capture the strength of the effect of evidence presented at the onset of the stimulus (A). Error bars and shading represent ±1 SEM of the mean. Plotting details in subsection “Plotting Procedure.” SEM = standard error of the mean; M = miscalibrated. See the online article for the color version of this figure.

We can also look at whether the diffusion mechanism assumed by the model, and fitted using confidence data, can account for the relationship between evidence and participants’ responses at different time lags. Looking at the data presented relative to trial onset (Figure 16A), the model simulations recreated in both conditions the qualitative pattern in the data of a decreasing effect of evidence fluctuations on choice over time. As was the case when looking at the relationship between evidence and confidence in the model fits, the model simulations produced a smaller effect of evidence on choice close to trial onset than the effect observed in the data, consistent with the conclusion that early evidence may be overweighted. At other times relative to trial onset, the effect of evidence on choice in the data and the model simulations was similar, although ±1 SEM windows for data and model did not always overlap. The model accounted reasonably well for the effect of evidence at time lags relative to response including, in the free response condition, capturing the distinctive pattern of an increase and decrease in the effect of evidence in the run up to a response (Figure 16B). The performance of the model simulation in this regard suggests the duration of the processing pipeline—which generates the decrease in the effect of evidence presented just prior to a response—was well estimated in the fits to confidence data alone. In the model-simulated data for the free response condition, evidence fluctuations at lags far before response favored the unchosen option, presumably because initial evidence fluctuations favoring the alternative is the only situation in which very long trials occur. However, data from participants did not clearly demonstrate this effect. This may be because in reality very long trials are the result of lapses (note that there was a trend in the data for the very longest responses; see also Figure 5 of Charles & Yeung, 2019). In the interrogation condition, model simulations produced an effect of evidence that matched on average the effect of evidence in the real data, but at times well before the response ±1 SEM windows for data and model did not overlap, and model simulations produced a smaller effect of evidence than observed in the data. This pattern is again consistent with the idea that in reality observers overweight evidence that they receive at the very beginning of trials.

These results demonstrate that, by using parameters estimated by fitting to confidence reports, and without fitting any additional parameters, we can generate reasonable response and response time data from the model. The results of these simulations are therefore consistent with the view, and can be explained by the idea, that the same underlying model that explains confidence reports also explains response times and decisions.

### Focusing on Response Time Distributions

The main goal of our article was to explore how far DDM-based models can account for confidence data. We have seen that such models can capture qualitative and precise quantitative patterns in confidence. In the Introduction section, we noted that even though the DDM has proved an extremely successful model of decision and response times, and even though our focus is on the open question of whether DDM models can also account for confidence, it remains important to assess whether good DDM-fits to confidence come at the cost of poor correspondences to decisions and response times: Our aim is to determine whether DDM-based models can account for confidence in addition to decisions and response times, not instead of them. We have already seen that fitting to confidence reports alone can produce a model that demonstrates reasonable correspondence to patterns in response and response time data. Nevertheless, we wanted to provide further evidence that fits to confidence do not compromise the ability of the DDM to account for decisions and response times.

We took decisions and response times further into account by performing fitting not just to confidence data alone. Instead we fit Model M to the confidence data while simultaneously minimizing an additional penalty. The penalty term was built from various sources and encouraged the model to also account for certain aspects of response and response time data. Details of the approach are provided in Appendix G. The approach is not systematic and in many ways limited, but the resulting performance of the model can be thought of as a lower bound. Specifically, the performance of the model resulting from this approach is a lower bound on the possible performance of the model when conducting a rigorous fitting exercise—that may become possible in the future—using the dynamic stimuli presented to model responses, response times, and confidence simultaneously and on a trial-by-trial basis.

**Figure 17:**
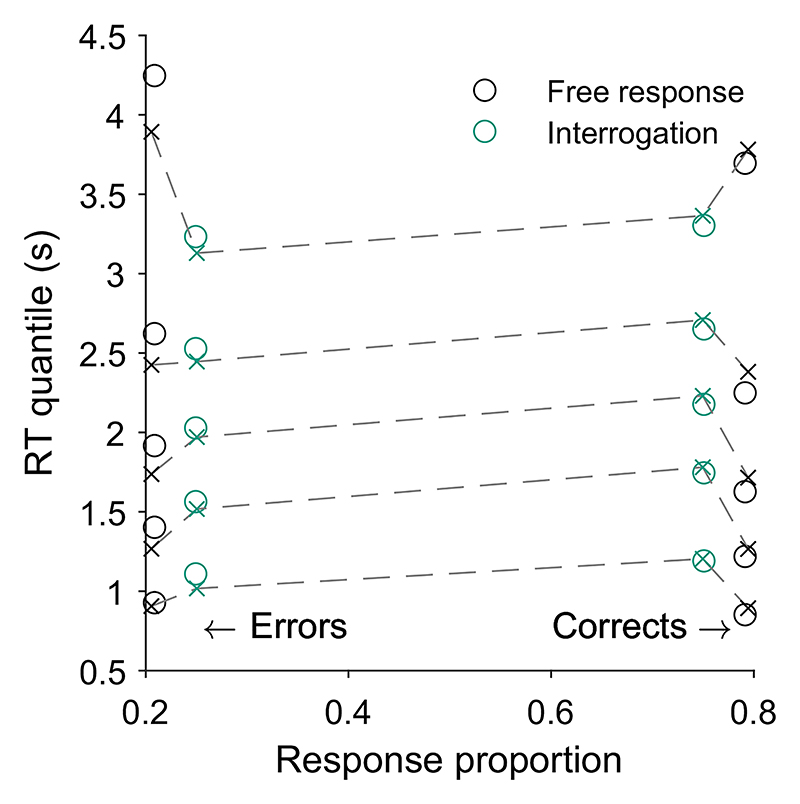
The Effect of Predecision and Pipeline Evidence on Confidence in the Two Conditions *Note*. Real data are shown with circles, while data simulated from the model are shown with crosses and connected by dashed lines. Following Ratcliff and McKoon (2008), for each participant and each unique combination of accuracy and condition (free response vs. interrogation), the 0.1, 0.3, 0.5, 0.7 and 0.9 quantiles of the response time distributions were calculated. The mean over participants is plotted on the *y*-axis. Data from a unique combination of accuracy and condition are plotted at the same *x*-value. This *x*-value represents the proportion of responses in that condition (free response or interrogation) that have the corresponding accuracy. Specifically, it is the mean of this value across participants. Note that this plotting procedure deviates from that described in subsection “Plotting Procedure.” Just as for other plots, the plot is based on trials in which a confidence report was obtained (meaning trials without a valid response in the interrogation condition are not included). RT = response time. M = miscalibrated. See the online article for the color version of this figure.

In Figure 17, we plot the correspondence between simulations from the resulting model and the real response and response time data. Specifically, we plot quantile probability functions (Ratcliff & McKoon, 2008), which simultaneously represent the quantiles of the response time distributions (*y*-axis) for each unique combination of accuracy and condition (in our case, free response and interrogation) and the proportion of correct and error responses in each condition (*x*-axis). We see that the model does a reasonable job of capturing the response time distributions and the response proportions: The qualitative pattern of greater accuracy in the free response condition than the interrogation condition is reflected in the model, and the model largely captures quantitatively the shape of the response time distributions in both conditions, both for error and correct trials. One exception is that the model slightly underestimates the speed of the slowest error responses in the free response condition. We reiterate that these correspondences can be viewed as a lower bound on the performance of the model that could be achieved with simultaneous trial-by-trial fitting of responses, response times, and confidence. Crucially, the fits of the model to confidence continued to capture both qualitatively and quantitatively the features of the data, even when the model was also encouraged to fit aspects of responses and response times (Appendix G; Figures G1 and G2). Hence, even with a suboptimal fitting approach, we can simultaneously achieve reasonable correspondences to response and response time data, and very good to excellent fits to confidence data. We discuss the future for this line of methodological and empirical research in General Discussion section.

## General Discussion

The motivation for the present research was a divergence between theories of how decisions are made and theories of how decisions are evaluated: Normative models of decision making prescribe that all available evidence should be considered when opting for a particular choice, a principle incorporated in influential frameworks such as the DDM that characterize the decision process in terms of tracking of the difference in evidence between two alternatives (Bogacz et al., 2006; Tajima et al., 2019). However, previous DDM-based accounts of confidence struggle with certain aspects of confidence data, and theories of confidence have often assumed that decisions are based on suboptimal mechanisms such as the race model or its variants. These theories provide intuitive accounts of confidence, but share the counterintuitive property that metacognitive evaluations of confidence are based on different and perhaps richer evidence than the decision itself (De Martino et al., 2013; Kepecs et al., 2008; Kiani et al., 2014; Moreno-Bote, 2010; van den Berg, Anandalingam, et al., 2016; Zylberberg et al., 2012; but see Moran et al., 2015; Pleskac & Busemeyer, 2010). For example, in race models with two evidence accumulators, a single accumulator may be used to trigger a response, whereas both evidence accumulators may be used to determine confidence.

In so far as we believe the foundational cognitive ability of perceptual decision making will be underpinned by a mechanism that is normative for typical perceptual decisions, and in so far as we are convinced of the empirical support for the ability of the DDM to account for decisions and response times (Introduction section), we have a strong interest in exploring ways in which we can improve upon the modeling of confidence within the DDM framework. An open question of special importance is whether a DDM can capture the strong relationship between confidence and response times (Pleskac & Busemeyer, 2010; Vickers & Packer, 1982).

We considered a core DDM and nine variants of this model that were motivated by theory and previous empirical research. They featured combinations of drift-rate variability (whereby different decisions vary in difficulty even when based on identical objective evidence), decreasing thresholds (whereby slower decisions are made based on a smaller balance of evidence), and Bayesian time penalties for confidence (whereby slower decisions are assumed to indicate more difficult decisions). Collectively, these features can explain benchmark findings regarding the relationship between confidence and trial difficulty, accuracy, speed emphasis, and response time, suggesting the viability of explaining confidence within a DDM framework. Four qualitative predictions shared by all nine variants of the core model were borne out in a novel experiment. This experiment assessed the relationship between confidence and stimulus evidence as a function of whether the choice over when to respond lies with the decision maker (and, hence, depends on a decision threshold) or is externally imposed: If decisions depend on a threshold, the possible balance of evidence at the time of decision is constrained (and, in principle, perfectly knowable) such that predecision evidence is substantially less predictive of confidence in free response tasks than in interrogation tasks. Developing this approach, we compared the DDM variants according to their detailed quantitative trial-by-trial predictions for confidence, capitalizing on the variability of the dynamic stochastic stimuli used in our study. To be precise, we modeled binned confidence data (see Models section for the rationale for treating confidence as an ordinal variable by binning, along with associated limitations). Model variants that featured a miscalibrated Bayesian readout of confidence fit the data best and provided an excellent account of the relationships between confidence and response time, and confidence and evidence, observed in both the free response and interrogation conditions.

There are wide implications of the finding that the DDM—which accounts well for decisions and response times (Ratcliff et al., 2016; Ratcliff & McKoon, 2008)—can precisely capture so many key patterns in confidence data. In particular, these results suggest that there is no need to abandon the claim that the mechanism responsible for one of the most basic cognitive functions, perceptual decision making, will reflect normative properties. On the contrary, these results strengthen the viewpoint that confidence reports are also generated by the same mechanism, normative for decision making, in which the difference in evidence is accumulated (Introduction section). The findings also support the idea that we can account for patterns in confidence data while maintaining that decisions and confidence result from the same evidence accumulation, instead of hypothesizing one accumulator for decisions and a second accumulator for confidence (Introduction section; Balsdon et al., 2020; Fleming & Daw, 2017; Ganupuru et al., 2019; Jang et al., 2012). This is attractive from a theoretical point of view because it means we are not committed to the idea that the brain accumulates noisy versions of exactly the same information twice. As a result, the brain only needs a single population of neurons to track evidence, reducing energy consumption (Lennie, 2003; see Ganupuru et al., 2019, for results in a different context that may be more difficult to account for using a single accumulator).

The key feature included in our best-fitting models was a miscalibrated Bayesian readout: Such observers look not just at the amount of evidence accumulated but also at the amount of time it takes to accumulate that evidence. If the same amount of evidence is gathered in two trials, but in one trial it takes longer to gather that evidence, the miscalibrated Bayesian observer interprets this trial as being more difficult, thereby reducing confidence. This time penalty applies regardless of whether the observer determines the time spent accumulating evidence through their response time (free response condition) or whether the experimenter determines the accumulation time (interrogation). For such observers, the time penalty applied does not perfectly match one based on the true variances associated with the various sources of variability that contribute to task difficulty. There are specific implications of the success of models with a miscalibrated Bayesian readout. The idea that confidence reflects a Bayesian readout has proved successful in the context of models of perceptual decisions based on two partially anticorrelated evidence accumulators (Kiani et al., 2014; van den Berg, Anandalingam, et al., 2016). We have seen that the idea of a Bayesian readout is also successful when using the framework of the DDM, building on work looking at the theoretical implications of such a model (Moreno-Bote, 2010), and complementing recent findings (Desender et al., 2020; Khalvati et al., 2020). That a DDM with a miscalibrated Bayesian readout can account for a wide range of patterns observed in previous research, and in this study, supports the view that confidence is Bayesian in the ongoing debate about this claim (Adler & Ma, 2018; Bertana et al., 2021; Caziot & Mamassian, 2021; Geurts et al., 2022; Kvam & Pleskac, 2016; Li & Ma, 2020; Meyniel et al., 2015; Navajas et al., 2017; Peters et al., 2017; Sanders et al., 2016).

Our results build on the 2DSD theory of Pleskac and Busemeyer (2010) by showing that DDM variants can not only explain decreasing confidence with response time but can successfully account for the strength of this relationship. 2DSD can predict some relationship between confidence and response time due to the presence of drift-rate variability, but struggles to account for the strength of this relationship (Introduction section; Pleskac & Busemeyer, 2010). It is surprising that Model V, which is closely related to 2DSD and contains drift-rate variability, did not outperform Model 0, which lacks drift-rate variability (Table 1; Figure 8). However, the models were not just fitting the decrease in confidence with response time that was observed in the free response condition. With the same parameter values for those parameters relevant to both conditions, the models needed to simultaneously account for the very different relationship between confidence and response time in the interrogation condition. In attempting to also fit data from the interrogation condition, parameters in Model V may have been driven to values at which any advantage over Model 0 in the free response condition was lost.

Although model comparison results were clear regarding the type of readout used for confidence (models with a miscalibrated Bayesian readout outperformed all others), it was unclear whether decreasing decision thresholds or drift-rate variability are also important: For example, Models M and DM, the latter of which includes decreasing decision thresholds, did not significantly differ in overall fit to our empirical data, when assessed using cross-validated log likelihoods. A random effects model comparison suggested that flat decision thresholds are more common, but it should be borne in mind that this latter analysis was based on BIC values, which tend to favor simpler models (Lewandowsky & Farrell, 2011). Similarly, models VM and VDM, which both feature drift-rate variability, performed fairly well when assessed on the basis of cross-validated log likelihoods, but the random effects model comparison pointed against this feature.

One explanation for why we did not find a clear result regarding decreasing decision thresholds is that humans use a decision threshold that only slightly deviates from flat, making time-dependence difficult to detect (Voskuilen et al., 2016). Consistent with this idea, studies investigating whether humans and animals use time-dependent thresholds have provided mixed results (Evans et al., 2020; Hawkins et al., 2015; Malhotra et al., 2017; Palestro et al., 2018; Pardo-Vazquez et al., 2019; Voskuilen et al., 2016). Another possible explanation is that we considered a straightforward implementation of decreasing decision thresholds, using linearly decreasing thresholds. Other options with additional parameters are possible, such as using a Weibull cumulative distribution function where the boundary collapses from an initial value, either toward an asymptotic value that may also be a free parameter (Glickman et al., 2019; Glickman & Usher, 2019; Hawkins et al., 2015) or alternatively simply collapses to zero (i.e., the starting point of the evidence accumulation; Evans et al., 2020). Other parameterizations have also been used (Hanks et al., 2011; Voskuilen et al., 2016). A more complex parameterization could be considered, although if strongly collapsing boundaries had been present it seems likely that a collapsing linear function would fit them better than static boundaries, even if their precise functional form was more complicated than linear. Furthermore, when more complicated functional forms have been used, the resulting boundaries are often such that a linear approximation could be reasonable, especially within the region of time containing most threshold crossings (Figure 2 in Evans et al., 2020; Figure5 in Voskuilen et al., 2016).

One further explanation is that our parameterization of the decision thresholds did not have too few parameters, but rather too many. One motivation for considering decreasing decision thresholds was that such thresholds can be optimal within the DDM framework when the difficulty of the task is unknown to the observer (Drugowitsch et al., 2012; Malhotra et al., 2018), such as is the case under the models considered here that feature drift-rate variability. However, we did not compare flat decision thresholds to the optimal decision thresholds. Instead we compared flat thresholds to decreasing thresholds, where we fit the slope of the threshold as a free parameter. Removing this free parameter by computing the optimal decision thresholds might allow us to draw stronger conclusions. Another promising approach to using nonlinear thresholds without adding model-fitting parameters would be to first perform model-free estimation of the shape of the decision thresholds. Glickman et al. (2022) presented a model-free approach to estimating time-varying decision thresholds from choice and response time data, and the shape of the thresholds are not constrained to take on a particular form. Thresholds inferred using this approach could be combined with the methods we presented to perform trial-by-trial modeling of confidence with arbitrarily complex decision thresholds.

Drift-rate variability is a central component of many DDM models (Ratcliff et al., 1999, 2016; Ratcliff & McKoon, 2008) and can be generated either because stimulus evidence strength varies on a trial-by-trial basis or because of internal fluctuations in the processing of constant evidence strength stimuli (Introduction section; Moran, 2015; Ratcliff et al., 2016). Throughout, we considered the more challenging case where no trial-by-trial variability is provided by the stimulus (only frame-by-frame variability; see Experimental Method section). Nevertheless, it may be surprising that we did not find evidence in favor of models featuring drift-rate variability. The explanation for this difference may be that we fit models only to confidence data. The inclusion of drift-rate variability in DDM accounts has been justified on the grounds that it explains key empirical phenomena regarding the speed of correct and error responses (Ratcliff et al., 1999, 2016; Ratcliff & McKoon, 2008). Thus, drift-rate variability may be an important part of explanations of patterns in responses and response times, but a less important part of understanding patterns in confidence.

This conclusion points toward a promising direction for future research: Performing trial-by-trial modeling by fitting simultaneously to responses, response times, and confidence reports, given the fluctuating stimulus shown on a particular trial. Although trial-by-trial modeling allows us to capitalize on the full richness and detail of the fluctuating-stimuli data sets we collect, by demanding that models take into account the unique properties of each trial (Park et al., 2016), it greatly increases the computational costs of model fitting. This is because the evaluation of a candidate set of parameters requires us to make unique predictions for every single trial, rather than simply for a few conditions. Here we made trial-by-trial fitting in a fluctuating-stimulus task feasible by using recently derived computationally cheap expressions for confidence (Calder-Travis et al., 2023) and did not fit to responses and response times, which in general—under free response conditions—demand far greater computational resources to model (Introduction section; Calder-Travis et al., 2023; Shinn et al., 2020; Smith, 2000; Tuerlinckx et al., 2001). Specifically, we made trial-by-trial predictions for, and fit to, confidence given the response, response time, and stimulus on each trial.

This advance in modeling of confidence is not in tension with the ultimate goal of generally applicable simultaneous trial-by-trial modeling of responses, response times, and confidence but rather is a step toward this important ultimate goal. In particular, two lines of theoretical and empirical work must reach a sufficient stage of development, and then be combined, to make such simultaneous trial-by-trial modeling feasible. One line is research looking into the predictions for confidence given responses, response times, and specific stimuli, such as those expressions used here. We have demonstrated that such expressions can be successfully used to fit, evaluate, and compare DDM-based models of confidence. The second line is research into reducing the computational cost of computing predictions for responses and response times, given the specific stimuli presented (e.g., Ratcliff, 1978, 1980; Shan et al., 2019; Smith, 2000; Smith & Ratcliff, 2022). Fast predictions currently only exist for limited cases (e.g., Navarro & Fuss, 2009). If faster broadly applicable predictions for responses and response times can be developed, we will be able to further push the limits of what DDM-based models can achieve in terms of simultaneously capturing precise quantitative patterns in responses, response times, and confidence. Previous work using condition-by-condition modeling (e.g., Desender et al., 2020; Kiani et al., 2014; Moran et al., 2015; Pleskac & Busemeyer, 2010; Ratcliff & Starns, 2009, 2013; van den Berg, Anandalingam, et al., 2016; Zylberberg et al., 2016), and the model fitting to confidence with an additional penalty term in Modeling Results section, already hint at the possible power of such an approach.

The model comparison we performed provided clear support for a miscalibrated Bayesian readout (Model M), but could this conclusion change to support other models, such as the calibrated Bayesian readout (Model VC), if trial-by-trial simultaneous modeling of choices, response times, and confidence becomes possible in the future? Several pieces of evidence support the robustness of the modeling conclusions reached on the basis of the trial-by-trial modeling of confidence conducted here. First, visual inspection of the fits in Figure 14B reveals that Model VC already fails to capture qualitatively and quantitatively key patterns in the confidence data, even without applying extra fitting constraints in the form of fitting to responses and response times. These fits to patterns in confidence data can only worsen when fitting to confidence, response times, and responses simultaneously. Hence, based on our results Model VC can be eliminated as a candidate for a DDM-based model of confidence. In contrast, even with extra penalty terms in the fitting to encourage fitting to response times and responses, Model M clearly outperforms Model VC (Figure G2A-Av) and achieves a good fit to patterns in the data. Second, the robustness of the modeling conclusions is supported through quantification of the goodness-of-fit for the models. Imposing additional constraints to fit choices and response times (as described in Appendix G) slightly increased the average −LLcv of confidence reports for Model M from 1.3384 to 1.3392, but this increased −LLcv was still lower than for all other models not featuring a miscalibrated Bayesian readout even before those additional constraints were used (e.g., Model VC without additional constraints has average −LLcv of 1.3509). This points strongly to the robustness of the modeling conclusions reached.

It is important to note that some features of the data remained unexplained by the best fitting model. The model could not explain why evidence presented at the onset of the stimulus had a stronger effect on responses and confidence than evidence presented later. We speculate that this effect arises because initial sensory samples are overweighted but acknowledge that changes to the decision mechanism itself might be able to account for this effect (Okazawa et al., 2018; Tsetsos et al., 2012). There are presumably numerous perceptual and cognitive phenomena that could be found in the data, but which we have not modeled, such as Weber’s law (Cobb, 1932), adaptive gain control (Cheadle et al., 2014), and confidence leak (Rahnev et al., 2015). From the perspective of models that do not incorporate these features, the data may look more noisy, with more unexplained variance, but the presence of other phenomena does not immediately invalidate an approach.

As described in Models section, we focused on exploring whether the DDM could be expanded to account for confidence reports, and we have not addressed the question of whether the DDM can be further extended to account for confidence response times. For example, we did not consider the possibility of a confidence threshold, following the decision threshold, which determines the time of the confidence report (Moran et al., 2015; Pleskac & Busemeyer, 2010). There were several motivations for this focus. First, using the parsimonious assumption that observers process and use all information presented in the stimulus to determine their confidence report, under our experimental design, we can make precise predictions for confidence, without needing to account for the wide range of possible mechanisms for terminating confidence computations (Models section; Bogacz et al., 2006; Moran et al., 2015). In this manner, we can separate out two difficult questions. By focusing on one of them, we can address this question in more detail than would be otherwise possible. Furthermore, this simplifying focus on confidence reports can be viewed as a strength of the models: We can explain rich patterns in confidence data without needing to postulate additional mechanisms.

Second, there is uncertainty about exactly what confidence response times reflect and evidence that they may be generated in a different way to decision response times. Pleskac and Busemeyer (2010) and Moran et al. (2015) successfully modeled the interval between the two-alternative perceptual decision and the confidence report and accounted for patterns that have been observed in such data. Notwithstanding these results, we note that in naturalistic situations there is often a clear cost to deliberating too long during perceptual decision making (e.g., crossing the road or determining if an animal is a predator), whereas confidence reports are often cued by external events, such as when our confidence is explicitly queried by someone else or when confidence is used to inform future information gathering choices (Bahrami et al., 2010; Desender et al., 2018). In addition, the finding that the speed of confidence reports is largely determined by the frequency with which each confidence report is made supports the idea that a separate mechanism from the evidence accumulation itself—such as some aspect of motor preparation—may be responsible for variability in the timing of confidence reports (Chen & Rahnev, 2023). If this is the case, the fastest progress to better joint models of confidence and confidence response times may come from first understanding the computations responsible for confidence reports, and then exploring how these lead to confidence response times, when the results of the confidence computations are reported on specific confidence scales.

It is also important to note that we have focused on a specific type of decision-making context throughout. Namely, we have explored confidence reports that follow two-alternative decisions. Confidence has been studied in a wide range of contexts, such as confidence following a multiple-alternative decision (Li & Ma, 2020), confidence in continuous judgments (e.g., orientation estimation; Bertana et al., 2021; Geurts et al., 2022), and confidence judgments made simultaneously with a response. This latter situation has been extensively studied in memory research (Heathcote, 2003; Murdock & Dufty, 1972; Ratcliff et al., 1992; Ratcliff & Starns, 2009, 2013), but also to some extent in the context of perception (Aitchison et al., 2015; Kiani et al., 2014). It may be that context-specific models are to some extent required, but the general principle “Bayesian confidence in optimal decisions” could be applied in all cases to guide future model development (although see Aitchison et al., 2015).

A comprehensive account of confidence in all contexts falls outside the scope of this work, nevertheless it is straightforward to start relating the models studied here to responses and confidence judgments given simultaneously. Findings from such studies present an important a priori challenge to any theory of confidence within a DDM framework, because if confidence is based on exactly the same evidence as the decision, it is not obvious why confidence could vary at the point of decision (as discussed above in the context of the 2DSD model). The idea that confidence reflects a Bayesian readout of evidence accumulated up to the time of the decision (Desender et al., 2020; Kiani et al., 2014) provides an important answer to this question, predicting reduced confidence for slower decisions given the same level of accumulated evidence. Hence, without any substantial modification, DDM-based models with a Bayesian readout predict higher confidence for faster decisions (under free response conditions), even when responses and confidence reports are made simultaneously. This is a pattern that has been observed (Murdock & Dufty, 1972). Whether such models can capture the entire range of patterns observed in the context of simultaneous responses and confidence reports remains an open question (Ratcliff & Starns, 2013).

A subtle but important detail is that, as implemented here, the models studied (including the winning Model M) cannot account for an effect of evidence that is independent of the effect of time, when there is no accumulation of pipeline evidence. Kiani et al. (2014) reported exactly such a finding: They used simultaneous responses and confidence reports and found that response time and evidence were both related to confidence, even when the other variable (evidence or response time) was held constant. However, this finding can be accommodated within our models if we make the common assumption that the duration of the sensory and motor processing pipeline varies from trial to trial (Luce, 1986; Ratcliff et al., 2004, 2016). In this case, the amount of time spent accumulating evidence up to the decision is no longer known by the researcher. Evidence becomes important (from the perspective of the researcher) and predicts confidence because it provides information about this timing and therefore about the strength of the time penalty for confidence. For example, lots of evidence presented for the chosen option suggests a short amount of time spent accumulating evidence up to a decision, and hence a reduced time penalty for confidence.

Given the successes of a relatively simple model in explaining varied features of confidence reports as a simple function of the accumulated evidence, penalized by the time taken to reach a decision, it is important to consider the concern that these models are too flexible and overpowerful in fitting the data. In particular, because by definition we do not constrain the observer’s generative model to match the true generative model, we might worry that Model M and its variants could fit any data set through flexibility in the specific form taken by the Bayesian readout for confidence (Bowers & Davis, 2012; Jones & Love, 2011; Rahnev & Denison, 2018). In this regard, we note first that, empirically, Model M did not seem to overfit training data: Model comparison using cross-validation showed that the miscalibrated Bayesian observer models were best at predicting data not used for training, suggesting that the extra flexibility of these models is warranted in this sense. Moreover, in a model recovery exercise (Appendix E), a miscalibrated Bayesian readout was never part of the winning model, unless the data had actually been generated with a miscalibrated Bayesian readout for confidence. More broadly, we would argue that our implementation of the miscalibration is a reasonable one, which held that observers in our experiment misestimated a key value (γ; Models section) that reflects the balance of various difference sources of variability. This idea is consistent with previous findings that humans deal poorly with noise introduced by stimulus variability (de Gardelle & Mamassian, 2015; Herce Castañón et al., 2019; Zylberberg et al., 2014, 2016). It seems plausible that observers misestimate the various sources of variability in our experiment specifically, given that our stimuli provided different amounts of evidence on each trial, due to frame-by-frame variability in evidence. Observers may have confused the frame-by-frame variability that is indeed present in the stimuli used, for trial-by-trial variability in the stimuli, and this seems especially plausible given the lack of trial-by-trial feedback (the distinguishing feature of trial-by-trial variability being that its effect is constant throughout a trial). This line of argument supports the view that a miscalibrated Bayesian observer model, of the kind considered here, is a sensible model to consider.

Although the modeling results consistently point toward the best fit of the miscalibrated Bayesian readout in the context we studied, it is plausible that in other contexts human observers achieve good calibration to the statistics of the task and hence use a calibrated Bayesian readout. In particular, those features that make a miscalibrated readout plausible for our task—namely, a lack of trial-by-trial feedback and the presence of additional sources of variability (frame-by-frame variability)—are clearly not present in all tasks and contexts. Where these features are not present and, for example, observers receive extensive trial-by-trial feedback, observers may be able to develop accurate representations of the statistics of the task, or improve their confidence reports over time, and therefore perform a calibrated Bayesian readout for confidence (Kiani et al., 2014; Ma & Jazayeri, 2014). There may also be task-specific or idiosyncratic biases that generate incorrect estimates of the magnitude of different sources of variability (de Gardelle & Mamassian, 2015). Such considerations may help explain why we did not find a significant positive relationship between response time and confidence in the interrogation condition, although this pattern has been found before (Pleskac & Busemeyer, 2010, Empirical Hurdle 5; Irwin et al., 1956; Vickers et al., 1985). If the estimation of task statistics by observers is task-dependent, the strength of the time penalty for confidence used and the relationship between time and confidence in the interrogation condition will also be task-dependent as a consequence. Therefore, in some contexts that differ from the context that we studied, we may expect human confidence to be best explained by a calibrated Bayesian readout. This in no way invalidates the proposition that human observers are performing a possibly miscalibrated Bayesian readout for confidence, from a DDM-based evidence accumulation.

In our model comparison, we did not compare a miscalibrated Bayesian observer with a range of non-Bayesian alternatives (Bowers & Davis, 2012). As a result, we accept that we have not provided strong evidence that confidence is based on a truly Bayesian computation. It is possible that the effects we observe arise from various mechanisms, which might include heuristic strategies that approximate the normatively prescribed computation via different mechanisms, but which might of course include computations with very different purposes and implementations that, for currently unclear reasons, nevertheless result in similar predictions for our data set. It was beyond the scope of our aims to rule out non-Bayesian alternatives. Our goal was more limited: We wanted to see if the DDM, coupled with plausible extensions such as a Bayesian readout, could provide an adequate account of confidence.

Notwithstanding the limitations discussed, we have seen that models in which decisions and confidence are generated by the same evidence accumulator, an accumulator that tracks the difference in evidence between two alternatives, can account for a wide range of qualitative and quantitative patterns in confidence. This provides a positive answer to the question we posed at the outset, of whether the normative and empirically successful DDM can be extended to account for confidence reports. Hence, we do not need to abandon the idea that the mechanism responsible for perceptual decisions will feature normative properties, or the idea that the brain will save on neural hardware when it can. Throughout, we have seen that one idea in particular, the idea of a miscalibrated Bayesian readout—that is, a Bayesian readout based on an imperfect internal model of the statistics of the world—provides a powerful framework for understanding and predicting confidence. Alongside the main empirical results, we have presented a methodological advance: We conducted model fitting to confidence data on a trial-by-trial basis, requiring the models to fit unique features of each trial.

## Supplementary Material

Supplementary Materials

## Figures and Tables

**Table 1:** Ten Model Variants Were Constructed Using the Core Model and Combinations of the Model Features

Model name	0	V	D	VD	VC	VDC	M	VM	DM	VDM
Drift-rate variability (V)	-	✓	-	✓	✓	✓	-	✓	-	✓
Decreasing thresholds (D)	-	-	✓	✓	-	✓	-	-	✓	✓
Bayesian confidence										
Calibrated (C)	-	-	-	-	✓	✓	-	-	-	-
Miscalibrated (M)	-	-	-	-	-	-	✓	✓	✓	✓

*Note*. Models are named using abbreviations of the features that they contain. ✓ = contains feature; - = does not contain feature.

**Table 2:** Observed and Predicted Patterns in Confidence Data (Part 1), Together With Explanations for These Patterns

Observed pattern	Features that can explain
(A) “conf. with signal”: Confidence increases as the strength of signal provided by the stimulus increases (Pleskac & Busemeyer, 2010, Hurdle 2; for example, Baranski & Petrusic, 1998, and Vickers & Packer, 1982)	Pipeline evidence (Pleskac & Busemeyer, 2010) Time penalty for conf. Decreasing thresholds
(B) “acc. and conf.”: Choice accuracy and confidence are positively correlated within individuals, even when considering trials of a fixed difficulty (Pleskac & Busemeyer, 2010, Hurdle 3; for example, Baranski & Petrusic, 1998, and Sanders et al., 2016)	Pipeline evidence (Pleskac & Busemeyer, 2010)
(C) “conf. with time (speed-acc)”: Confidence increases with response time when comparing different conditions in which there is different emphasis on trading speed for accuracy, and in settings where stopping is enforced at a particular time, such as in the interrogation condition (Pleskac & Busemeyer, 2010, Hurdle 5; for example, Rosenbaum et al., 2022, and Vickers & Packer, 1982)	DDM accounts of conf. (Ratcliff & McKoon, 2008)
(D) “conf. with time (free)”: For free response tasks, within a single speed-accuracy condition, confidence decreases with response time (Pleskac & Busemeyer, 2010, Hurdle 4; for example, Rosenbaum et al., 2022, and Vickers & Packer, 1982)	Pipeline evidence and drift variability (partial explanation only; Pleskac & Busemeyer, 2010) Time penalty for conf. Decreasing thresholds

*Note*. The explanations are discussed in detail in the main text. See Table 3 for Part 2. conf. = confidence; acc. = accuracy; DDM = drift diffusion model.

**Table 3:** Observed and Predicted Patterns in Confidence Data (Part 2), Together With Explanations

Observed pattern	Features that can explain
(E) “conf. resolution”: Confidence is a stronger predictor of accuracy following free responses that are speeded, compared to free responses where participants are asked to emphasize accuracy (Pleskac & Busemeyer, 2010, Hurdle 8; Baranski & Petrusic, 1994)	Variable interjudgment times (Pleskac & Busemeyer, 2010) Time penalty for conf.
(F) “conf. in errors”: In some cases confidence in errors decreases as a task becomes easier, in other cases it increases (Kiani et al., 2014, and Sanders et al., 2016)	Pipeline evidence and time penalty for conf. and flat decision threshold (Calder-Travis et al., 2023; Desender et al., 2020; Khalvati et al., 2020; Kiani et al., 2014)
(G) “conf. with IJT”: Time between decision and confidence (when controlled by the experimenter) decreases confidence on error trials, but this IJT has a relatively small effect on correct trials (Yu et al., 2015)	Evidence leak (Yu et al., 2015) Time penalty for conf.
Predicted pattern	Feature that can explain
(H) “evidence on conf.”: In free response tasks, once the effect of time has been accounted for, predecision evidence will have a smaller effect on confidence than pipeline evidence. There will be no analogous effect in interrogation tasks	Decisions and conf. from same thresholded accumulation

*Note*. See Table 2 for Part 1. conf. = confidence; IJT = interjudgment time.

**Table 4:** Parameters in the Models

Model	0	V	D	VD	VC	VDC	M	VM	DM	VDM	All
Accumulator noise (σ_acc_)											1
Metacognitive noise (σ_*m*_)											1
Decision threshold height (*a*)											1
Pipeline duration (*I*)											1
Confidence lapse rate (λ)											1
Confidence bin bounds (*d*_*i*_)											3
Drift-rate variability (σ_φ_)		1		1	1	1		1		1	
Decision threshold slope (*b*)			1	1		1			1	1	
Estimated variability ratio (Γ)							1	1	1	1	
Total	8	9	9	10	9	10	9	10	10	11	

*Note.* All models shared the eight parameters of the core model. Model variants with additional features contained additional parameters. Drift-rate variability introduced a parameter (standard deviation of distribution over the drift-rate scaling), decreasing decision thresholds introduced a parameter (threshold slope), and a miscalibrated Bayesian readout for confidence also introduced one additional parameter (estimated variability ratio). Models VC and VDC have the same parameters as Models V and VD, respectively, but are not the same model. Models VC and VDC feature a Bayesian readout of confidence (which introduces no additional parameters; Table 1). Details of the role of the parameters in the computational model are provided in Appendix A. V = drift-rate variability; D = decreasing thresholds; C = calibrated; M = miscalibrated.

**Table 5:** Model Variants Added Only for the Random Effects Model Comparison

Model name	C	DC
Drift-rate variability (V)	-	-
Decreasing thresholds (D)	-	✓
Bayesian confidence		
Calibrated (C)	✓	✓
Miscalibrated (M)	-	-

*Note*. These models are not included in most analyses because they exactly duplicate the predictions of other models (see Table 1 and Models section). ✓ = includes this feature; - = does not include this feature.

## Footnotes

1 There is a typographical error in the preregistration. In the preregistration document, the predictions are lettered differently to here. The first time “prediction B” (as lettered in that document) is mentioned, it does not make sense. Our intent is clarified by looking at the stated statistical test for Prediction B. We made one small deviation from the preregistration. For the ordinal regression analysis, we excluded any trial in which the number of frames prior to response was less than, or equal to, the estimate of the pipeline duration for the participant. We preregistered “less than,” not “less than or equal to.” We did not run the analysis in the “less than” case.
