# Supplementary material for: Bayesian Confidence in Optimal Decisions
Source: Psychol Rev. Author manuscript; Available in PMC 2025 Feb 24. (PMC7617410; doi:10.1037/rev0000472)
Supplement: Supplementary Materials [file EMS202997-supplement-Supplementary_Materials.pdf]

Appendix A

Mathematical Details of the Models

Here, we describe the mathematical framing of the models. We largely use the framework of Calder-Travis et al. (2023), but with some extensions. We follow the convention that log indicates the natural logarithm.

Evidence Accumulation

Changes in the state of the accumulator are determined by the number of dots in the two stimuli arrays of the frame currently being processed. If a frame is presented for 50 ms, then it is processed for 50 ms, and it correspondingly determines the average rate of evidence accumulation for 50 ms. If E_{ij} is the number of dots presented in array i in stimulus frame j, then over a small time step δt, during which frame j is being processed, the probability distribution over the increment in the accumulator, δx, is given by (Drugowitsch et al., 2012; Moreno-Bote, 2010; Ratcliff, 1978),

$$p(\delta x | E_{1j}, E_{2j}, \varphi) = N\left(\delta x; \varphi(E_{2j} - E_{1j})\frac{\delta t}{t_f}, \sigma_{\mathrm{acc}}^2 \delta t\right). \tag{A1}$$

$N(y;\, a,\, b)$ indicates a normal distribution over y with mean, a, and variance, b. φ is the drift-rate scaling. t_f is the duration of one stimulus frame and is included as a convenience to give the resulting evidence accumulation an intuitive scale (the average change in the accumulator due to a single stimulus frame will just be the difference in the number of dots in the two arrays). σ_{acc}, one of the free parameters, determines the level of noise in accumulator increments.

In models in which the drift-rate scaling is constant, $\varphi = 1$ on all trials. In models where the drift-rate scaling varies, it follows a normal distribution (Ratcliff, 1978, 1980; Ratcliff & McKoon, 2008), with the level of variability set by the free parameter σ_φ:

$$p(\varphi) = N(\varphi; 1, \sigma_\varphi^2). \tag{A2}$$

The accumulator begins at 0 and a response is made when it reaches one of the two decision thresholds. The position of the upper and lower thresholds are given by $a - bt$ and $-a + bt$ where t is the time spent accumulating evidence and a and b are free parameters. For models with flat decision thresholds, $b = 0$. The duration of sensory and motor processing pipelines is also a free parameter, I. Hence the response time is given by I plus the time spent accumulating evidence up to one of the decision thresholds.

Confidence Readouts

Calder-Travis et al. (2023) found expressions for the probability of a confidence report $C = i$, given a response R, time of response t_r, and evidence stream $\boldsymbol{E}$, in both the free response and interrogation conditions. The derivations covered variability in drift-rate scaling, time-dependent thresholds, and metacognitive noise, but they only considered one form of readout for confidence. Specifically, they assumed a noisy but otherwise Bayesian readout of confidence, based on a correct generative model of the environment. Here, we consider how to extend the derivations to cover confidence that reflects a readout of the final state of the accumulator, and confidence that reflects a miscalibrated Bayesian readout.

In the existing expressions (Calder-Travis et al., 2023), the observer maps the DDM accumulator state, x, to a (scaled) readout of the log-posterior ratio between the two options, x_{lp}. The mapping can be summarized by a single function, $\theta(t_e)$, of the time spent accumulating evidence, t_e,

$$x_{lp} = \frac{x}{\theta(t_e)}. \tag{A3}$$

The division of accumulated evidence, x, by $\theta(t_e)$ is the effect that we refer to as the time penalty for confidence. Substituting in all abbreviations used in Calder-Travis et al. (2023),

$$\theta(t_e) = \frac{t_f^2\sigma_{\mathrm{acc}}^2 + t_f\sigma_E^2 + t_e\Delta\mu^2\sigma_\varphi^2}{t_f^2\sigma_{\mathrm{acc}}^2 + t_f\sigma_E^2 + \Delta\mu^2\sigma_\varphi^2}, \tag{A4}$$

where, t_f is the duration of a "frame," over which evidence presented in the stimulus is constant (for the experiment above this was 50 ms). Evidence presented in the stimulus for the two options is sampled from two distributions each frame. $\Delta\mu$ is the absolute value of the difference between the means of these two distributions. $\Delta\mu$ squared indicates $(\Delta\mu)$ squared. $\sigma_E/\sqrt{2}$ is the standard deviation of each of these two distributions. σ_{acc} is the standard deviation of noise that corrupts the incoming information as it is added to the accumulator, and σ_φ is the standard deviation in the drift-rate scaling across trials. We can write this expression more simply as,

$$\theta(t_e) = 1 - \gamma + \gamma t_e, \tag{A5}$$

where

$$\gamma = \frac{\Delta\mu^2\sigma_\varphi^2}{t_f^2\sigma_{\mathrm{acc}}^2 + t_f\sigma_E^2 + \Delta\mu^2\sigma_\varphi^2}. \tag{A6}$$

Depending on the response, either the (scaled) log-posterior ratio, x_{lp}, or its negative, $-x_{lp}$, are monotonically related to the probability of being correct (because log is a monotonic function and the posterior ratio gives the ratio of the probabilities that each of the two options are correct). Hence, $1/\theta(t_e)$ converts the final state of the accumulator into a readout that determines the confidence of a calibrated Bayesian observer. We can change $\theta(t_e)$ to model observers who do not use a calibrated Bayesian readout. For an observer whose confidence reflects a noisy readout of the final state of the accumulator itself, we can just set $\theta(t_e) = 1$, so that $x_{lp} = x$.

In the case of an observer who uses a miscalibrated Bayesian readout of confidence, we allow the possibility that the observer incorrectly estimates the magnitude of difference sources of variability. The observer's readout will have the same form as that in Equation A5, except that any variability terms will be replaced by their estimated value, rather than their true value. One important complication is that if the observer incorrectly estimates the magnitude of several sources of variability, these effects can cancel out leading to identical behavior. For this reason, we cannot uniquely identify the observer's estimate of all the different sources of variability. Instead, we simply model the observer's estimate of

(*Appendices continue*)

the relative magnitude of different sources of variability. In particular, we simply fit the observer's estimate of γ,

$$\hat{\gamma} = \frac{\Delta\mu^2 \hat{\sigma_\varphi}^2}{t_f^2 \hat{\sigma_{\text{acc}}}^2 + t_f \hat{\sigma_E}^2 + \Delta\mu^2 \hat{\sigma_\varphi}^2}, \quad \text{(A7)}$$

where, a hat on a variable indicates that it is the observer's estimated value for that variable, not the true value. The equivalent of Equation A5 for the miscalibrated Bayesian observer then becomes,

$$\theta(t_e) = 1 - \hat{\gamma} + \hat{\gamma} t_e. \quad \text{(A8)}$$

We do not fit $\hat{\gamma}$ directly, but a transformed version:

$$\Gamma = -\log\left(\frac{1}{\hat{\gamma}} - 1\right). \quad \text{(A9)}$$

Γ is not bounded at 0 and 1, but can take any value.

For all readouts, we allow the possibility that the readout is corrupted by metacognitive noise (De Martino et al., 2013; Maniscalco & Lau, 2012). Hence, x_{lp} does not determine confidence directly, but a noisy version of it, x_c,

$$p(x_c|x_{lp}) = N(x_c; x_{lp}, \sigma_m^2). \quad \text{(A10)}$$

σ_m is a free parameter for the level of metacognitive noise. The value of x_c or $-x_c$ (signed according to the response such that a greater number indicates more support for the choice made) relative to confidence bin boundaries then determines the confidence report given. If x_c (or $-x_c$) falls between d_i and d_{i+1}, then a confidence report in bin i is given.

Lapses

Calder-Travis et al. (2023) did not consider lapses. Here, we include the possibility that on a certain proportion of trials observers give a random confidence report.

We assume that the probability of a lapse in the confidence report given does not depend on the response, response time, or evidence stream, with some exceptions. That is, generally,

$$p(L = 0|R, t_r, \boldsymbol{E}) = 1 - \lambda, \quad \text{(A11)}$$

$$p(L = 1|R, t_r, \boldsymbol{E}) = \lambda, \quad \text{(A12)}$$

where $L = 1$ and $L = 0$ denote the presence or absence of a lapse. There is one exception in the free response condition and one in the interrogation condition. In the free response condition, a free parameter of the model describes the total delay caused by sensory and motor processing pipelines (I). If any response in the free condition is faster than this estimated delay, then the corresponding trial is treated as a lapse. In the interrogation condition, to avoid numerical problems, we treat trials as certainly the result of a lapse when the following is true of the probability of the response given: $p(R|\boldsymbol{E}) < 10^{-15}$.

When a lapse occurs we assume it leads to all possible confidence reports with equal probability,

$$p(C = i|L = 1, R, t_r, \boldsymbol{E}) = M, \quad \text{(A13)}$$

where, M is a constant. Marginalizing over whether or not a lapse has occurred provides us with our final predictions for confidence,

$$p(C|R, t_r, \boldsymbol{E}) = p(L = 0|R, t_r, \boldsymbol{E}) p(C|L = 0, R, t_r, \boldsymbol{E}) + p(L = 1|R, t_r, \boldsymbol{E}) p(C|L = 1, R, t_r, \boldsymbol{E}), \quad \text{(A14)}$$

$$= (1 - \lambda) p(C|L = 0, R, t_r, \boldsymbol{E}) + \lambda p(C|L = 1, R, t_r, \boldsymbol{E}). \quad \text{(A15)}$$

The probability of a confidence report in the absence of a lapse, $p(C = i|L = 0, R, t_r, \boldsymbol{E})$, is given by the results of Calder-Travis et al. (2023). These results apply directly in the case of a calibrated Bayesian readout, and they apply once adjusted according to the considerations in the previous subsection in the case of a direct readout of the state of the accumulator or in the case of a miscalibrated Bayesian readout.

Changes for Implementation

In the implementation of the derivations, we made a number of low-level changes from the expressions in Calder-Travis et al. (2023). First, in the experiment the stimulus only cleared at the end of each 50 ms frame, not immediately at the time of a response. We accounted for this additional <50 ms of evidence presented to the participants. On a trial-by-trial basis, we treated this additional evidence in the same way as evidence from the processing pipeline.

Second, we precomputed values on a trial-by-trial basis for total pipeline evidence, and for mean predecision evidence. We precomputed these values assuming that the processing pipeline contained evidence presented in the 0 ms, 50 ms, or 100 ms, … prior to the end of the stimulus, and assuming that the initial 0 ms, 50 ms, or 100 ms, … of evidence presented in the stimulus was processed before a decision. We then linearly interpolated between these values to determine the pipeline or predecision evidence, given a hypothesized time at which a decision threshold was reached.

Third, it turned out that the log likelihood of the model varied in a discontinuous way as the parameter corresponding to the duration of the processing pipeline, I, changed. This was due to the fact that trials with response times shorter than the pipeline duration were classed as certain lapses. To avoid the difficulties of finding the maximum of a nonsmooth function, we evaluated the likelihood function at the two closest values to I that were multiples of 50 ms and linearly interpolated between these to estimate the likelihood function at I (when simulating confidence reports from fitted models, no such interpolation was necessary, and we simply rounded the processing pipeline to the nearest multiple of the duration of a frame, i.e., the nearest multiple of 50 ms).

(*Appendices continue*)

Appendix B

Simulation Details

Confidence Simulations

To plot data against model fits (e.g., to plot figures in the "Model Comparison" and "Fits of the Best Model" subsections of Modeling Results section), we simulated one confidence report for each real trial containing a valid confidence report, on the basis of the response, response time, and evidence presented on that trial, and in accordance with the model predictions for $p(C|R, t_r, \boldsymbol{E})$.

Diffusion Simulations

For other simulations we took a different approach and simulated completely new synthetic trials and stimuli, and then simulated the full diffusion process from the onset of the trial, to the end of all stimulus processing, including the decision threshold crossing in the free response case.

We simulated stimuli with properties that matched those used in the main experiment (Experimental Method section). The evidence accumulation, decision mechanism, and confidence reporting followed the models (Models section). In the interrogation condition, response time was simulated to be the time that the stimulus cleared, plus the duration of sensory and motor processing pipelines, I, consistent with the idea that in this condition participants process all the available information—with evidence accumulation lasting precisely as long as the duration of stimulus presentation—and then make a response (Models section). We simulated the internal evidence accumulation using time steps of size 0.1 ms, comparing the accumulator to the decision thresholds (in the free response condition) after each time step. In accordance with the models, at each time step the drift rate was determined by the stimulus frame currently being processed. Specifically, the drift rate was determined by the difference between the number of dots in the two arrays, multiplied by the drift-rate scaling.

Simulating Responses and Response Times and Focusing on Response Time Distributions

For the plots of model-simulated response times and accuracy (in "Simulating Responses and Response Times" and "Focusing on Response Time Distributions" subsections of Modeling Results), we ran the diffusion simulations as described, with some small differences. On each simulated trial, a random trial from the corresponding participant in the main experiment was selected. The distributions that were used on this trial to determine the number of dots in the high and low evidence arrays were then used in the simulated trial. Trial durations in the interrogation condition were sampled, on a participant-by-participant basis, from the trial durations used in the interrogation condition in the real experiment. We simulated 6,400 trials per participant for the subsection "Simulating Responses and Response Times" and 3,200 for the subsection "Focusing on response time distributions."

Model Recovery

We simulated data sets for model recovery in almost the same way as we simulated data for the "Simulating Responses and Response Times" subsection of Modeling Results section. For model recovery, we simulated the same number of trials as there are in the real data set (640), because we wanted to test whether the analysis was successful with the number of trials available. For these simulations, we also applied a time limit of 60 s to free response trials, to prevent the simulated data set taking up too much memory on disk. After this time limit the simulated decision thresholds collapsed to almost zero.

Appendix C

Ordinal Regression Details

We repeatedly used a probit ordinal regression onto binned confidence. Predictors in these regressions were individually z-scored. Following the fitting of the probit regression model to the data, we applied a transformation to the resulting coefficients. Namely, we applied the reverse transformation to that which was generated by z-scoring. We did this so that coefficients associated with evidence predictors conveyed effects per unit of evidence (i.e., stimulus dots), rather than per standard deviation of predictor. For example, a greater coefficient for predecision evidence than pipeline evidence means that, if the evidence in one predecision frame was boosted by 10 dots, this would have a stronger effect on confidence than boosting one pipeline frame by 10 dots.

The transformation that, when applied to the coefficients, reverses the effect of z-scoring the predictors, requires some thought. Denote one of the predictors for a single trial x, and the z-scored version $\tilde{x}$. Then,

$$\tilde{x} = \frac{x - \mu}{\sigma}, \tag{C1}$$

where μ and σ are the mean and standard deviation used for z-scoring.

In an ordinal regression, a coefficient, β, and the corresponding predictor for a single trial, $\tilde{x}$, are multiplied (Long, 1997), $\beta\tilde{x}$. Substituting in our expression for $\tilde{x}$,

$$\beta\tilde{x} = \beta\frac{x - \mu}{\sigma} = \frac{\beta}{\sigma}x - \frac{\beta\mu}{\sigma}. \tag{C2}$$

We see that the coefficient associated with the raw evidence predictor (i.e., evidence in units of dots) is given by β/σ. Hence, we divided the coefficients resulting from the regression by the standard deviation used for z-scoring of the corresponding predictors.

(*Appendices continue*)

Appendix D

Response Distributions

We combined all trials from all participants and, by dividing the data into 200 equally spaced bins, looked at the distribution over response times and confidence reports (Figure D1). Efforts to match response times in the two conditions achieved reasonable results. Note that in the interrogation condition, there is some time between the end of the stimulus and the response that the end of the stimulus triggers. Hence, in the interrogation condition the stimulus will be presented for a shorter amount of time than the response time, unlike in the free response condition. Confidence reports were recorded on a continuous scale from −90 to 90, with 0 corresponding to a report of "Don't know" (Experimental Method section).

Figure D1

The Distributions Over (A) Response Times and (B) Confidence Reports in the Two Conditions

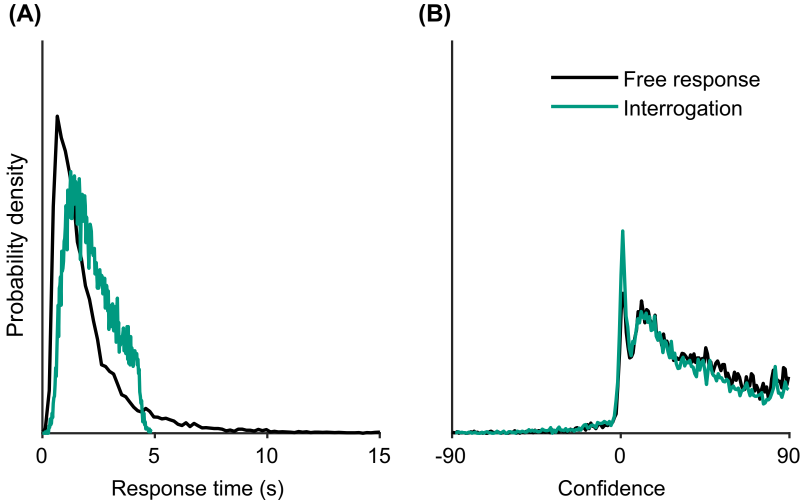

Note. All trials were included, including trials in which a confidence report was not obtained. See the online article for the color version of this figure.

Appendix E

Assessment of Model-Fitting Performance

We performed a model recovery analysis. We simulated data sets from each of the 10 model variants and then fit all 10 models to these 10 simulated data sets. We looked to see if the true data generating model was recovered when the models were compared. To provide a particularly stringent test, we simulated data sets of the same size as the real data set, using fitted parameters for each participant (from fits to the entire data set without division into training and test trials). Using the fitted parameters generates a particularly strong test because these parameters have been tuned so that all models make similar predictions to the behavioral data, and hence also to each other. On each simulated trial, we simulated the full diffusion process from the onset of evidence accumulation, through decision, to confidence report (Appendix B).

We fit all simulated data (without partitioning into training and test data) and used AIC and BIC to compare models. We looked at AIC and BIC instead of the cross-validated log likelihoods used on the real data in Modeling Results section because we did not have access to sufficient computational resources to run cross-validation on 10 simulated data sets. Ideally, we would use identical model comparison procedures for the model recovery and the analysis of the real data. Therefore, we also reran the main model comparison for the real data by fitting to the real data set (without partitioning into training and test data), and then assessing the models using AIC and BIC. This approach produced similar results to the cross-validation—specifically, models with a miscalibrated Bayesian readout performed best—and it uses an identical model comparison procedure to the procedure used in the model recovery.

In the model recovery analysis with BIC, the true generating model was often recovered (Figure E1). The BIC was sometimes conservative regarding the presence of drift-rate variability and decreasing decision thresholds: Sometimes the best fitting model was a simpler version of the data generating model. In one case (when the data generating model was VDC) the BIC was also conservative

(*Appendices continue*)

about the presence of a Bayesian readout for confidence, with the best fitting model not having this feature. Note that the random-effects model comparison analysis in the main text was based on BIC values.

Performance of the AIC was similar (Figure E2), although it was less conservative than the BIC, as expected (Lewandowsky & Farrell, 2011). Under data generating Model V, Model V fit very well, but Model VC fit slightly better. Using 95% bootstrapped confidence intervals, we verified that this difference was not significant, and therefore that no false positive would have been reached under these circumstances.

Figure E1

Mean BIC Obtained After Fitting Data Simulated Using One of the 10 Models

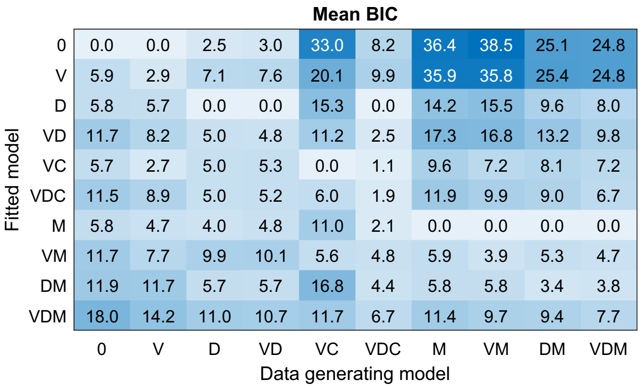

Note. BIC = Bayesian information criterion; V = drift-rate variability; D = decreasing thresholds; C = calibrated; M = miscalibrated. See the online article for the color version of this figure.

Figure E2

Mean AIC Obtained After Fitting Data Simulated Using One of the 10 Models

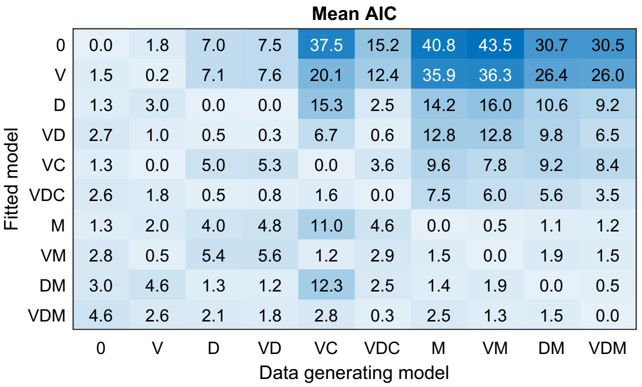

Note. AIC = Akaike information criterion; V = drift-rate variability; D = decreasing thresholds; C = calibrated; M = miscalibrated. See the online article for the color version of this figure.

Appendix F

Parameter Bounds, Start Points, and Fitted Values

During optimization using MATLAB's fmincon optimizer (Matlab Optimization Toolbox, 2017), we specified bounds on the values the parameters could take (Table F1). At the start of each fit candidate parameter sets were drawn from uniform distributions between the "Lower initial" and "Upper initial" values in Table F1. To support efficient fitting we offset and scaled some parameters, so that all parameters covered a similar range. The offset and scaling values used are also provided in the table. Details of the roles of the parameters in the computational model are provided in Appendix A.

Fitted parameter values for the three models on which we focused in Modeling Results section are provided below. Values are from the fits performed on the entire data set (i.e., where the data set was not divided into training and test data).

Table F1

Parameter Bounds, Offset, and Scaling Used During Fitting, and Limits for Initial Parameter Values

Parameter	Lower bound	Lower initial	Upper bound	Upper initial	Offset	Scaling
Drift-rate variability (σ_φ)	0	0	6	2	0	1/6
Accumulator noise (σ_{acc})	0	200	24,000	8,000	0	1/24000
Confidence bin bounds (d_i)	−36,000	−12,000	72,000	24,000	36,000	1/108000
Decision threshold height (a)	10	100	72,000	24,000	−10	1/72000
Decision threshold slope (b)	0	0	72,000	24,000	0	1/72000
Pipeline duration (s) (I)	0.001	0.05	1	0.8	0	1
Lapse rate (λ)	1/640	0.01	1	0.4	0	1
Metacognitive noise (σ_m)	1	50	72,000	24,000	−1	1/72000
Estimated variability ratio (Γ)	−20	−3	20	3	0	1/20

(*Appendices continue*)

Table F2
Fitted Parameter Values

Model	Parameter	*Mdn*	25th percentile	75th percentile
V	σ_φ	0.16	0.14	0.54
	σ_{acc}	860	170	1,400
	d_i	980	−560	1,700
		4,000	2,900	7,200
		6,900	4,800	12,000
	a	3,000	1,600	5,600
	I (s)	0.60	0.50	0.80
	λ	0.50	0.0018	0.70
	σ_m	1900	140	6,900
VC	σ_φ	0.89	0.55	1.8
	σ_{acc}	1800	920	3,000
	d_i	1,100	110	1900
		4,400	3,100	7,700
		8,300	5,900	15,000
	a	5,200	3,200	9,400
	I (s)	0.38	0.15	0.67
	λ	0.13	0.0016	0.44
	σ_m	3,700	1,100	7,400
M	σ_{acc}	2,100	1,400	3,800
	d_i	1,200	180	1,600
		3,000	2,400	4,600
		5,300	4,500	7,900
	a	3,600	2,600	6,500
	I (s)	0.50	0.28	0.80
	λ	0.0016	0.0016	0.35
	σ_m	2,400	1,000	3,600
	Γ	0.32	−0.91	1.8

Note. V = drift-rate variability; C = calibrated; M = miscalibrated.

Appendix G

Toward Simultaneous Fitting of Responses, Response Times, and Confidence

Fitting With an Additional Penalty Term

As outlined in Modeling Results section in the subsection "Focusing on Response Time Distributions," we took a first step toward simultaneous trial-by-trial fitting of responses, response-times, and confidence, by fitting to confidence data but also including an additional penalty term that was to be minimized. Specifically, instead of fitting by maximizing the likelihood in Equation 2, we fit Model M to the data by maximizing the following quantity:

$$\prod_i \{p(C^{(i)}|\boldsymbol{\xi}, \mathbf{E}^{(i)}, R^{(i)}, t_r^{(i)})e^{-P^{(i)}}\}, \quad \text{(G1)}$$

which is the likelihood multiplied by a penalty term. $P^{(i)}$ quantifies the penalty for the ith trial. The penalty, $P^{(i)}$, for free response trials was the same for all such trials and was calculated as follows,

$$P^{(i)} = 50P_1 + 20P_2. \quad \text{(G2)}$$

The terms in the penalty were each motivated in different ways but each was expected to encourage the model to achieve a better correspondence to response and response time data. The scaling of each term was set to ensure, in a very approximate manner, no individual term dominated.

P_1 was given by,

$$P_1 = \frac{1}{N_f}\left|\frac{1}{N_f}\sum_{i=1}^{N_f}\left\{t_d^{(i)} - \frac{A(x_d^{(i)})}{\frac{1}{t_f}\Delta\mu}\right\}\right|. \quad \text{(G3)}$$

The outer vertical lines indicate the absolute value. $\Delta\mu$ and t_f are as defined in Appendix A. N_f is the number of free response condition trials being analyzed and the summation is taken over all such trials. $t_d^{(i)}$ indicates the time from onset of evidence accumulation to decision threshold crossing for the ith trial and can be inferred by subtracting the duration of sensory and motor processing pipelines, I, from the time taken to respond on the ith trial, $t_r^{(i)}$. $x_d^{(i)}$ indicates the state of the DDM evidence accumulation at the time of threshold crossing (i.e., this is the height of the decision threshold). $A()$ is a function that signs its input such that $A(x_d^{(i)})$ is positive if the threshold corresponding to the correct option was reached and negative if the threshold for the incorrect option was reached.

This term was motivated by the fact that, under certain conditions for a discrete-time diffusion process (i.e., the system evolves in discrete time steps), certain relations are known to hold. One is that the average number of steps taken before reaching a decision threshold equals the average state of the evidence accumulator at the time of threshold crossing, divided by the average change in the

(*Appendices continue*)

evidence accumulator per time step (Equation 10.28 in Shadlen et al., 2006). This only holds if the drift rate is the same on every trial. For model M no drift-rate variability was present, and we can always consider the diffusion process from a perspective such that the sign of the drift rate does not depend on the correct response: Instead of considering the diffusion process from a perspective in which positive values correspond to evidence for one response, and negative values the other response, we simply consider the process from a perspective in which positive values correspond to evidence for the correct choice, and negative values correspond to evidence for the incorrect choice. Hence, P_1 is expected to be approximately zero (in the absence of drift-rate variability), and it can therefore be used as an extra goal in our fitting procedure.

P_2 has a very similar motivation: Again there is a relation known to hold under certain conditions (Equation 10.21 in Shadlen et al., 2006), meaning that the following term should be close to zero,

$$P_2 = \frac{1}{N_f}\left|\frac{1}{N_f}\sum_{i=1}^{N_f}\left\{\exp\left(-\frac{2\Delta\mu}{t_f\sigma_{\text{acc}}^2+\sigma_{\text{E}}^2}A(x_d^{(i)})\right)\right\}-1\right|. \quad \text{(G4)}$$

The summation is again over the free response trials being analyzed. σ_{acc} and σ_E are defined in Appendix A.

While P_1 and P_2 provide further fitting goals for the free response condition, different goals were used to compute the penalty, $P^{(i)}$, for interrogation condition trials. The penalty for interrogation condition trials was constructed out of one term that was the same for all trials, and one term that varied on a trial-by-trial basis. Specifically, it was calculated as follows,

$$P^{(i)} = 50P_3 + P_4^{(i)}. \quad \text{(G5)}$$

P_3 is rather straightforward and simply reflects the fact that we expect the average response time in the interrogation condition to reflect the average duration of stimulus presentation plus the duration of the processing pipeline, I.

$$P_3 = \frac{1}{N_I}\left|\frac{1}{N_I}\sum_{i=1}^{N_I}\left\{t_r^{(i)} - t_e^{(i)} - I\right\}\right|. \quad \text{(G6)}$$

Here N_I is the number of interrogation condition trials being analyzed, and the summation was taken over these trials. $t_e^{(i)}$ is the duration of evidence presentation on the ith trial (and by assumption also the total time spent accumulating evidence; Models section). $t_r^{(i)}$ is the response time on the ith trial.

For $P_4^{(i)}$, we used the fact that it is relatively simple to calculate the probability of a response on a trial-by-trial basis in the interrogation

Figure G1

Fit of the Best Model (Model M) to the Number of Confidence Reports in Each Confidence Bin in (A) the Free Response Condition and in (B) the Interrogation Condition, When Also Using a Penalty Term During Fitting

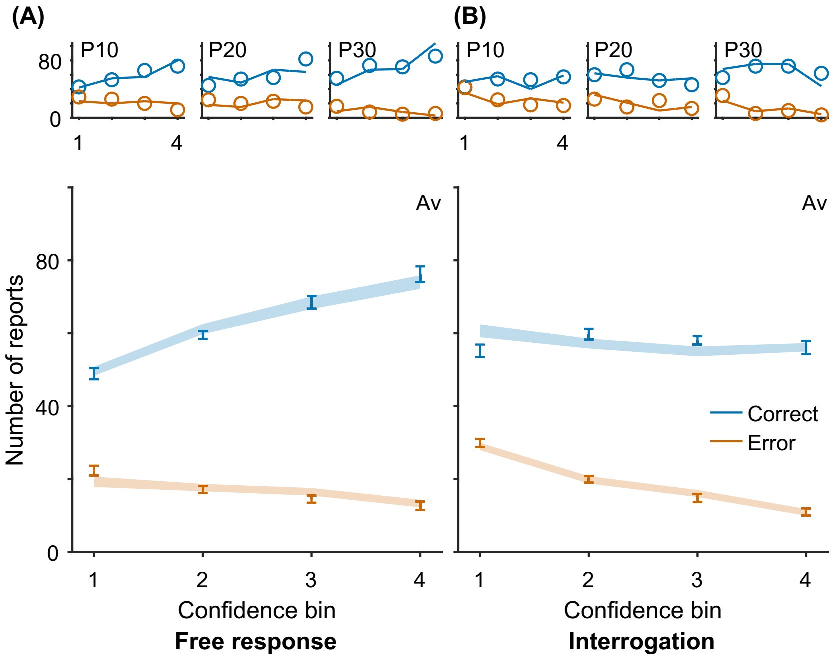

Note. (A-Av and B-Av) Model fit is shown in shading and the data with error bars. For these panels error bars and shading represent ±1 SEM. A-P10, A-P20, A-P30, B-P10, B-P20, B-P30 show corresponding data (circles) and model fits (lines) for three individual participants. Plotting details in subsection "Plotting Procedure." M = miscalibrated; Av = average; SEM = standard error of the mean. See the online article for the color version of this figure.

(*Appendices continue*)

Figure G2

(A-Av) Effect of Response Time and (B-Av) Average Evidence on Confidence in the Data (Error Bars) and in the Best Fitting Model, Model M (Shading), When Also Using a Penalty Term for Fitting

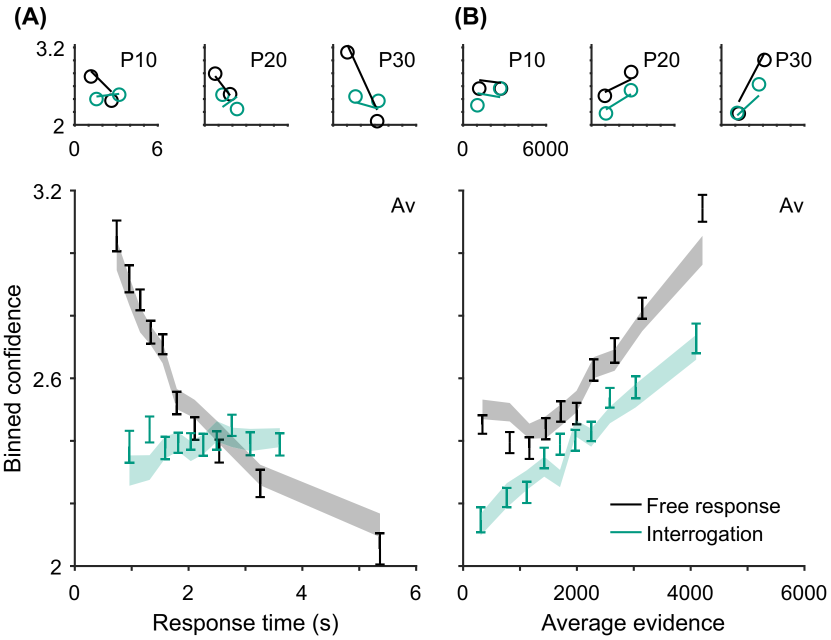

Note. In Panel B average evidence is computed by summing, over all frames, the difference in dots presented in the two arrays, before taking the absolute value and dividing by the time the stimulus was presented for. In both A-Av and B-Av error bars and shading represent ±1 SEM. A-P10, A-P20, A-P30, B-P10, B-P20, B-P30 show corresponding data (circles) and model fits (lines) for three individual participants. Plotting details in subsection "Plotting Procedure." Av = average; SEM = standard error of the mean; M = miscalibrated. See the online article for the color version of this figure.

condition. We computed the probability of a given response for such trials using the expression provided in Calder-Travis et al. (2023) for $p(R^{(i)}|\boldsymbol{E}^{(i)})$. Here $R^{(i)}$ and $\boldsymbol{E}^{(i)}$ indicate the response and stimulus on the ith trial. From this we constructed the following penalty for the ith interrogation condition trial,

$$P_4^{(i)} = -\log\left(\frac{39}{40}p(R^{(i)}|\boldsymbol{E}^{(i)}) + \frac{1}{80}\right). \tag{G7}$$

In addition to the penalty term, there was one further difference between the model fitting conducted for this analysis and the main model-fitting procedure described in Modeling Method section: Instead of rerunning each fit 40 times, we only reran each fit 10 times.

Results for Confidence

We saw in the main text that Model M, when fitting using the additional penalty term, did a reasonable job of capturing patterns in response and response time data. Of course, if the penalty term helps improve the correspondence to responses and response times, but this comes at the cost of poor fits to confidence data, then we have made no progress toward simultaneously modeling all features of the data. We therefore looked at the fits to confidence that resulted from this supplemented model-fitting procedure. As shown in Figure G1 and Figure G2, fits remained excellent.

Received September 23, 2021
Revision received December 19, 2023
Accepted December 30, 2023 ■
